# Will gut, oral, and vaginal microbiota influence the outcome of FET or be influenced by FET? A pilot study

**DOI:** 10.1128/mbio.00509-25

**Published:** 2025-06-17

**Authors:** Zhao Zhang, Yiwen Zhang, Aihua Wu, Chengfang Xu, Zhe Li

**Affiliations:** 1 Department of Reproduction, Southern Medical University Affiliate Dongguan People’s Hospital 594334 , Dongguan, Guangdong, China; 2 Department of Obstetrics, The Third Affiliated Hospital of Sun Yat-sen University, Guangzhou, Guangdong, China; Louis Stokes Cleveland VA Medical Center, Cleveland, Ohio, USA

**Keywords:** frozen embryo transfer, gut microbiota, oral microbiota, vaginal microbiota, metabolism

## Abstract

This study aims to examine the specific relationships between gut, oral, and vaginal microbiota and the frozen embryo transfer (FET) process. Patients undergoing fertility treatment who met the inclusion criteria were included in this study. After sampling at three time points, participants were then divided into two groups: the failure group and the success group, based on whether a viable intrauterine pregnancy was confirmed. In this pilot study, we systematically examined changes in the gut, oral, and vaginal microbiota at various stages of the FET process using 16S rDNA high-throughput sequencing and investigated their respective associations with FET outcomes. Metabolomics and random forest were used for evaluating the relationship between gut microbiota and metabolites during FET. Our findings indicate that while the gut microbiota underwent the least change throughout FET, it exhibited the greatest differences between success and failure groups. The oral and vaginal microbiota exhibited significant fluctuations. However, the differences in oral microbiota between the success and failure groups changed with the FET process, while the vaginal microbiota did not show any differences. Notably, two key gut genera, *Anaerococcus* and *Negativicoccus*, were identified as genera significantly associated with FET outcomes. Additionally, specific gut microbiota and metabolite profiles displayed significant correlations with FET success, particularly highlighting the potential relevance of cystamine before FET. These findings suggest that targeting microbiota-associated metabolic pathways may serve as a potential strategy to enhance FET success rates and provide new biomarkers for clinical prediction and intervention.

**IMPORTANCE:**

This study explores the potential role of microbiota in influencing FET outcomes. Through an analysis of gut, oral, and vaginal microbiota, we observed notable differences between success and failure groups, particularly in gut microbiota. Genera such as *Anaerococcus* and *Negativicoccus*, along with associated metabolic profiles, may offer insights into underlying mechanisms. These findings contribute to a growing understanding of the interplay between microbiota and reproductive outcomes and suggest that targeting microbiota-associated metabolic pathways could be a promising direction for enhancing FET success rates. This research highlights potential biomarkers and therapeutic avenues for further exploration in fertility treatments.

## INTRODUCTION

With the widespread use of assisted reproductive technologies (ARTs), the number of *in vitro* fertilization (IVF) births continues to increase, and the live birth rate for infertility patients has significantly improved. Some of the most commonly used techniques in ARTs include IVF, intracytoplasmic sperm injection, biphasic *in vitro* maturation (IVM), embryo freezing and frozen embryo transfer (FET), preimplantation genetic testing, and non-invasive preimplantation genetic testing (1). The role of microbiota during IVF remains controversial. Some studies have shown that vaginal microbiota does not change with the use of vaginal progesterone (2) or during IVF treatment (3). However, other studies suggest that the vaginal microbiome varies during IVF (4). Most research indicates that genital tract microbiota have been found to be associated with pregnancy outcomes in IVF patients (5, 6). For example, Moreno et al. found that endometrial microbiota are associated with poor reproductive outcomes in IVF patients (7). Additionally, previous studies have reported associations between the composition of the vaginal and cervical microbiota and IVF outcomes (8).

The oral cavity hosts the second-largest microbial community in the human body (9). Elevated levels of sex hormones increase oral vascular permeability and place a significant burden on the host’s immune system, which can alter the balance of the oral microbiome (10). As a result, oral microbiota changes during pregnancy. Oral microbiota has also been linked to adverse pregnancy outcomes, including low birth weight, pre-eclampsia, inevitable abortion, and preterm birth (11). In some cases, prevalent oral microbiota has been isolated from the vagina or placenta of pregnant women with adverse outcomes (12–16). There are two ways in which the oral microbiota may influence the reproductive tract. First, periodontal bacteria may translocate from an unhealthy oral cavity, cross the placenta, and enter the intra-amniotic fluid and fetal circulation, directly affecting the fetoplacental unit and leading to bacteremia (17). Second, endotoxins and/or inflammatory mediators from periodontal plaque and subgingival inflammation may disseminate systemically, ultimately reaching the fetoplacental unit (18, 19). However, no research has specifically examined the relationship between oral microbiota and IVF outcomes.

Our previous research demonstrated that gut microbiota can change during pregnancy (20). Moreover, vaginal and gut microbiota have a complex relationship (21–23). Some studies suggest that gut microbiota is related to infertility (24). A previous study found that gut microbiota-derived metabolites may influence IVF outcomes (25). In our study, we aim to explore the correlation among oral, gut, and vaginal microbiota at different stages of FET and the outcomes of FET. Additionally, we will investigate whether these correlations are related to microbial metabolites. Our goal is to explore significant biomarkers that could improve the clinical outcomes of FET.

## MATERIALS AND METHODS

### Study design and participants

A total of 59 patients undergoing fertility treatment at the outpatient clinic of Southern Medical University Affiliate Dongguan People’s Hospital were included in this study. The exclusion criteria for participants were as follows: (i) gastrointestinal disease or a family history of such conditions; (ii) antibiotic use within the past 30 days; (iii) hypertension, diabetes mellitus, hyperthyroidism, hypothyroidism, autoimmune diseases, or other endocrine and metabolic disorders; (iv) a history of blood transfusions, organ transplantation, or immunotherapy; (v) bacterial vaginosis, vulvovaginal candidiasis,trichomonal vaginitis, *Chlamydia trachomatis*, *Ureaplasma urealyticum*, *Neisseria gonorrhoeae* infections, or other vaginal symptoms such as itching or abnormal discharge; (vi) oral disease or having undergone oral care within the past 30 days; and (vii) endometriosis and/or adenomyosis.

**Fig 1:**
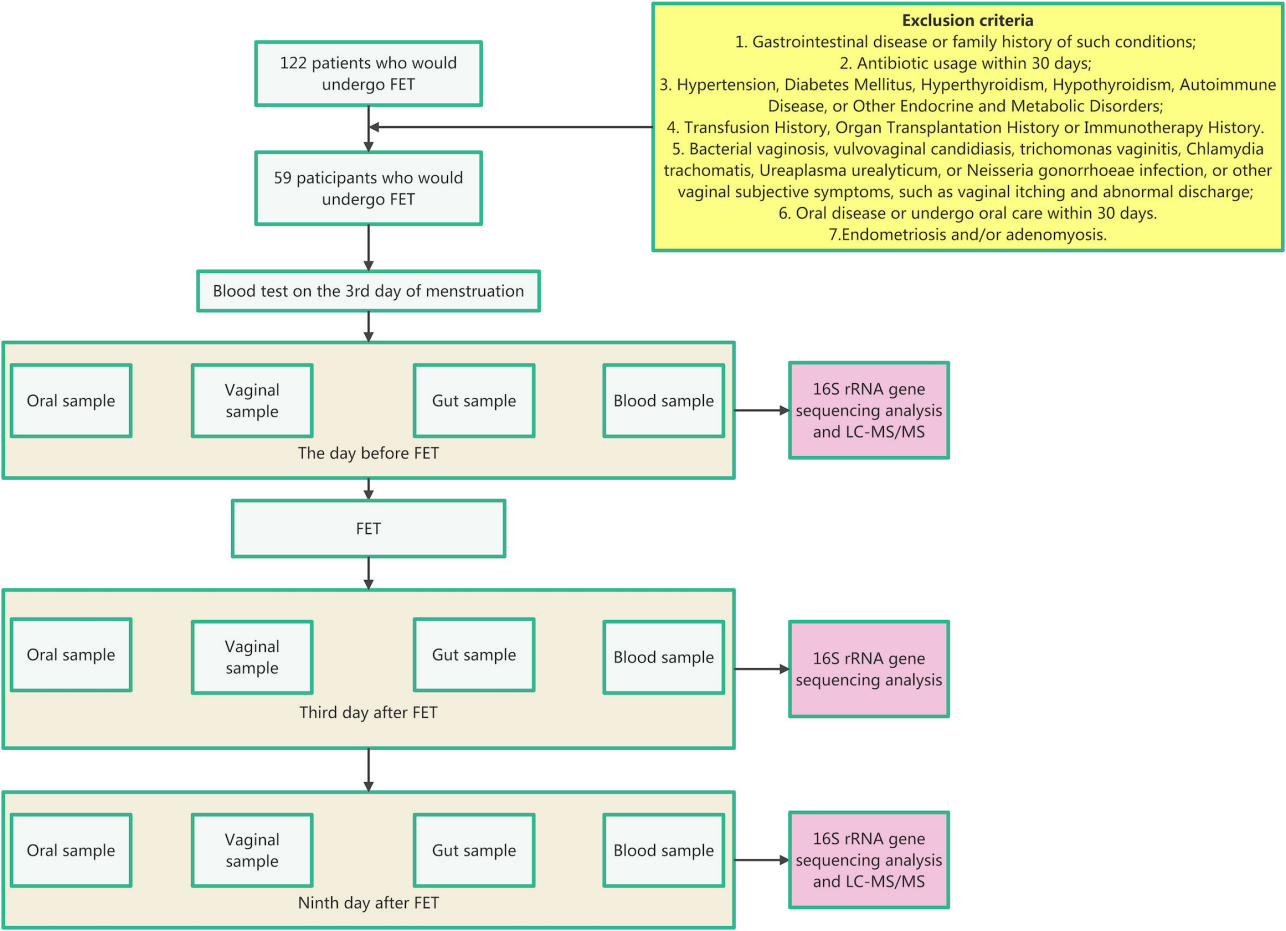
Flowchart of our study. LC-MS/MS, liquid chromatography–tandem mass spectrometry.

The flowchart of our study is presented in Fig. 1. To better understand the influence of microbiota and metabolites on FET outcomes, participants were then divided into two groups: the failure group (*n* = 29) and the success group (*n* = 30), based on whether a viable intrauterine pregnancy was confirmed. Participants in the success group had a viable intrauterine pregnancy confirmed by the 9 week ultrasound. In our study, the microbiota from different sites is labeled with the following symbols: “G” represents the gut; “O” represents the oral cavity; and “V” represents the vagina. Time points A, B, and C refer to the day before FET, the third day after FET, and the ninth day after FET, respectively. Additionally, “F” indicates the failure group, while “S” represents the success group (Table 1).

**TABLE 1:** Definition of study group symbols

Symbol	Meaning
G	Gut
O	Oral cavity
V	Vagina
A	Day before FET
B	Third day after FET
C	Ninth day after FET
F	Failure
S	Success

### FET protocol

FET was conducted according to the following protocol. Endometrial preparation was performed using hormone replacement cycles. When the endometrial stripe thickness, as measured by ultrasound, was ≥8 mm with a proliferative pattern, daily progesterone was administered via vaginal suppositories, intramuscular injections, or oral tablets. Frozen–thawed embryo transfer was then performed on either day 4 or 6 of progesterone support. Patients were instructed to continue progesterone treatment until the end of the first trimester of pregnancy or until a negative pregnancy blood test was obtained on day 14 after embryo transfer.

### Fecal collection

Feces from patients were collected three times: the day before FET, the third day after FET, and the ninth day after FET. Feces were obtained in the hospital and transferred to the laboratory freezer (−80℃) within 30 min of collection (26, 27)

### Vaginal secretion collection

Vaginal secretion from patients was collected three times: the day before FET, the third day after FET, and the ninth day after FET. Individuals were instructed to abstain from sexual activity, refrain from cleansing the vulva or vagina, and avoid using vaginal medication within 48 hours prior to sample collection. Prior to sample collection, all patients underwent laboratory testing of vaginal discharge to exclude bacterial vaginosis (Nugent score ≥ 7), vulvovaginal candidiasis, trichomonal vaginitis, *Chlamydia trachomatis*, *Ureaplasma urealyticum*, and *Neisseria gonorrhoeae* infections. A vaginal speculum was used to expose the vagina, and aseptic cotton swabs were used to collect vaginal discharge from one-third of the lateral vaginal wall. The specimens were promptly preserved at −80°C until subsequent 16S rDNA gene sequencing analysis.

### Oral sample collection

Oral samples from patients were collected three times: the day before FET, the third day after FET, and the ninth day after FET. Participants were instructed to avoid brushing their teeth, eating, or drinking 30 min before sampling. Thorough sampling of the teeth, gums, saliva, and mucous membranes was performed on both sides of the cheeks. Oral samples were obtained in the hospital and transferred to the laboratory freezer (−80℃) within 30 min of collection.

### Blood sample collection

Blood samples from patients were collected twice: the day before FET and the ninth day after FET. Approximately 3 mL of venous blood was collected from all patients by nurses. After collection, the blood specimens underwent centrifugation at a speed of 4,000 r/min for 10 min. Subsequently, the upper serum layer was carefully extracted into freezing tubes using a pipette gun and transferred to the laboratory freezer (−80℃) within 30 min of collection.

### DNA extraction

Microbial genomic DNA was extracted from fecal samples using the magnetic bead-based Fecal Genomic DNA Extraction Kit (TianGen, Beijing, China), following the manufacturer’s protocol. For vaginal and oral swab samples, DNA was extracted using the cetyltrimethylammonium bromide method. The concentration and purity of the extracted DNA were evaluated by 1% agarose gel electrophoresis. DNA was then diluted to 1 ng/µL using sterile water and stored at –20°C until further use.

### Amplicon generation

The 16S rRNA genes of distinct regions were amplified in the V3–V4 region using the primers 341F (CCTAYGGGRBGCASCAG) and 806R (GGACTACNNGGGTATCTAAT), with the barcode (File S1). All PCRs were carried out with 15 µL of Phusion High-Fidelity PCR Master Mix (New England Biolabs), 0.2 µM forward and reverse primers, and approximately 10 ng of template DNA. Thermal cycling consisted of initial denaturation at 98°C for 1 min, followed by 30 cycles of denaturation at 98°C for 10 s, annealing at 50°C for 30 s, and elongation at 72°C for 30 s and 72°C for 5 min. A negative control (ddH_₂_O replacing template DNA) was included in each PCR run to monitor potential contamination; only when no amplification was observed in the negative control did we proceed with subsequent steps.

### PCR product quantification and qualification

The PCR products were purified using magnetic bead purification. Samples were mixed in equidensity ratios based on the concentration of the PCR products. After thorough mixing, the PCR products were detected, and the target bands were recovered.

### Library preparation and sequencing

Sequencing libraries were generated, and indices were added. The library was checked with a Qubit and real-time PCR for quantification and a bioanalyzer for size distribution detection. The quantified libraries were pooled and sequenced on Illumina platforms according to the effective library concentration and data amount needed.

### Paired-end reads assembly and quality control

#### Data split

Paired-end reads were assigned to samples based on their unique barcode and were truncated by removing the barcode and primer sequence.

#### Sequence assembly

Paired-end reads were merged using FLASH (v.1.2.11, http://ccb.jhu.edu/software/FLASH/) (28), a very fast and accurate analysis tool that was designed to merge paired-end reads when at least some of the overlapping reads were generated from the opposite end of the same DNA fragment. The resulting splicing sequences were called raw tags. For our sequencing, both ends were sequenced to 250 bp, making the total read length 500 bp.

#### Data filtration

Quality filtering of the raw tags was performed using fastp (v.0.23.1) software to obtain high-quality clean tags (29). Sequences with a Phred quality score below -q 19 were removed. Reads containing more than 15% low-quality bases (-u 15) were discarded.

#### Chimera removal

The tags were compared with the Silva database (https://www.arb-silva.de/) to detect chimera sequences, and the effective tags were obtained by removing the chimera sequences with the vsearch package (v.2.16.0, https://github.com/torognes/vsearch) (30) (File S2).

### Amplicon sequence variant denoising and species annotation

#### Amplicon sequence variant denoising

For the effective tags obtained previously, denoising was performed with DADA2 to obtain the initial amplicon sequence variants (ASVs).

#### Species annotation

Species annotation was performed using QIIME2 software. The annotation database used was the Silva Database.

#### Phylogenetic relationship construction

To study the phylogenetic relationship of each ASV and the differences in the dominant species among different samples (groups), multiple sequence alignment was performed using QIIME2 software.

#### Data normalization

The absolute abundance of ASVs was normalized using a standard sequence number corresponding to the sample with the fewest sequences. Subsequent analyses of alpha diversity and beta diversity were all performed based on the output normalized data.

#### Relative abundance

The top 10 taxa of each sample at each taxonomic rank at the phylum level and the top 30 at the genus level were selected to plot the distribution histogram of relative abundance in Perl (v.5.26.2) through the SVG function.

### Metabolite extraction

The samples (100 µL) were placed in the Eppendorf tubes and resuspended with prechilled 80% methanol by well vortex. Then the samples were incubated on ice for 5 min and centrifuged at 15,000 × *g* at 4°C for 20 min. Some of the supernatant was diluted to a final concentration containing 53% methanol by liquid chromatography–mass spectrometry grade water. The samples were subsequently transferred to a fresh Eppendorf tube and then were centrifuged at 15,000 × *g* at 4°C for 20 min. Finally, the supernatant was injected into the liquid chromatography–tandem mass spectrometry (LC-MS/MS) system analysis (31, 32).

### Ultra-high-performance liquid chromatography–tandem mass spectrometry analysis

Ultra-high-performance liquid chromatography–tandem mass spectrometry (UHPLC-MS/MS) analyses were performed using a Vanquish UHPLC system (Thermo Fisher, Germany) coupled with an Orbitrap Q ExactiveTM HF mass spectrometer or Orbitrap Q Exactive HF-X mass spectrometer (Thermo Fisher) in Novogene Co., Ltd. (Beijing, China). Samples were injected onto a Hypersil Gold column (100.0 × 2.1 mm, 1.9 µm) using a 12 min linear gradient at a flow rate of 0.2 mL/min. The eluents for the positive and negative polarity modes were eluent A (0.1% formic acid in water) and eluent B (methanol). The solvent gradient was set as follows: 2% B, 1.5 min; 2%–85% B, 3 min; 85%–100% B, 10 min; 100%–2% B, 10.1 min; 2% B, 12 min. Q Exactive HF mass spectrometer was operated in positive/negative polarity mode with a spray voltage of 3.5 kV, capillary temperature of 320°C, sheath gas flow rate of 35 psi and auxillary (aux) gas flow rate of 10 L/min, S-lens RF level of 60, and aux gas heater temperature of 350°C.

### Data processing and metabolite identification

The raw data files generated by UHPLC-MS/MS were processed using the Compound Discoverer (v.3.3) (CD3.3, Thermo Fisher) to perform peak alignment, peak picking, and quantitation for each metabolite. The main parameters were set as follows: peak area was corrected with the first QC, actual mass tolerance, 5 ppm; signal intensity tolerance, 30%; and minimum intensity, etc. After that, peak intensities were normalized to the total spectral intensity. The normalized data were used to predict the molecular formula based on additive ions, molecular ion peaks, and fragment ions. Then peaks were matched with the mzCloud (https://www.mzcloud.org/), mzVault, and MassList databases to obtain the accurate qualitative and relative quantitative results. Statistical analyses were performed using the statistical software R (R v.R-3.4.3), Python (v.2.7.6), and CentOS (release 6.6). Then we combined positive and negative ionization mode results to make our results clearer.

### Alpha diversity

To analyze the diversity and richness of the communities in the sample, alpha diversity was calculated from three indices in QIIME2, including Chao1, Shannon, and Simpson. One index was selected to identify community richness: Chao (the Chao1 estimator, https://scikit.bio/docs/latest/generated/skbio.diversity.alpha.chao1.html#skbio.diversity.alpha.chao1); two indices were used to identify community diversity: Shannon (the Shannon index, https://scikit.bio/docs/latest/generated/skbio.diversity.alpha.shannon.html#skbio.diversity.alpha.shannon) and Simpson (the Simpson index, https://scikit.bio/docs/latest/generated/skbio.diversity.alpha.shannon.html#skbio.diversity.alpha.shannon ).

### Beta diversity

To evaluate the complexity of the community composition and compare the differences among samples (groups), beta diversity was calculated based on weighted UniFrac distances in QIIME2.

Non-metric multidimensional scaling (NMDS) (33) was implemented for data dimension reduction. NMDS uses the distance matrix but instead emphasizes the numerical rank. The distance between sample points on the diagram can only reflect the rank information rather than the numerical differences. NMDS analysis was implemented through R software with the ade4 package and ggplot2 package.

### Group characteristics analysis

Linear discriminant analysis effect size (LEfSe) is widely used to discover biomarkers and can reveal metagenomic characteristics (34). To achieve this goal, an exclusive package named lefse (1.1.01) was utilized.

### Association analysis

To explore the symbiotic relationship between species and to reveal the environmental factor influence on community structures, the Spearman correlation test and Benjamini–Hochberg false discovery rate (FDR) correction were used to reflect the correlation between environmental factors and species abundance. All of the diagrams and analyses were completed in R.

### Operational taxonomic unit biomarker identification

A random forest model was used to select significantly different operational taxonomic units (OTUs) in each sample group. The generalization error was estimated by a 10-fold cross-validation. An OTU frequency profile was generated by mapping reads from the BCG and BPG groups onto these represented sequences (35). A cross-validation error curve was plotted after a 10-fold cross-validation. The cutoff point was that with the lowest cross-validation error. The sum of the minimum error and the SD at the corresponding point was defined as the cutoff value. All sets of OTU markers with errors below the cutoff value are listed. The optimal set with the fewest OTUs, which revealed the differences between the two groups with the highest accuracy, was identified.

Subsequent analyses, such as receiver operating characteristic (ROC) analysis, were then performed. Statistical significance was determined with a Wilcoxon rank-sum test (*P* < 0.05) (36). The ROC curve was plotted to evaluate the diagnostic efficacies of the selected biomarkers, and the area under the curve (AUC) was also calculated using pROC (R v.3.8.1) (37).

### Co-occurrence network analysis

Spearman’s rank correlation coefficient was conducted through R package of “ccrepe” between genera, based on the relative abundance profile of genera. Networks were then constructed by using the method implemented in Gephi (v.0.10.1) (38).

### Metabolite analysis

These metabolites were annotated using the Kyoto Encyclopedia of Genes and Genomes (KEGG) database (https://www.genome.jp/kegg/pathway.html). Partial least squares discriminant analysis (PLS-DA) was performed at metaX (39) (a flexible and comprehensive software for processing metabolomics data). We applied univariate analysis (*t*-test) to calculate the statistical significance (*P* value). The metabolites with variable importance in projection (VIPs) of >1 and *P* values of <0.05 were considered to be differential metabolites. Volcano plots were used to filter metabolites of interest based on log2(fold change) and −log10(*P* value) of metabolites by ggplot2 in R language. The functions of these metabolites and metabolic pathways were studied using the KEGG database.

### Statistical analysis

All measurement data are presented as the mean ± standard error. All enumeration data are presented as numbers. The difference in clinical characteristics between the successful group and the failure group was analyzed using independent-sample *t*-tests. Multiple testing correlation was applied using the Benjamini–Hochberg FDR method, and *q* < 0.05 was considered statistically significant. The difference in characteristics of the transferred embryos between the successful group and the failure group was analyzed using the chi-square test, and *P* < 0.05 was considered statistically significant. All data were analyzed using SPSS statistical software (v.29.0.2.0; SPSS, Inc., Chicago, IL, USA)

## RESULTS

The baseline clinical characteristics of all participants, as well as comparisons between the failure and successful groups, are summarized in Table 2. No significant differences were observed between the two groups in terms of age, body mass index, duration of infertility, number of IVF attempts, baseline hormone levels, endometrial thickness, or lipid and glucose metabolism indicators (*q* > 0.05). The only statistically significant difference was day 9 human chorionic gonadotropin (HCG) levels, which were significantly higher in the successful group (*q* < 0.05), confirming implantation success. These findings suggest that conventional clinical parameters may not be strong predictors of FET outcomes.

**TABLE 2:** Clinical characteristics in the failure group and the successful group^*a*^

Parameter	All participants (*n* = 59)	Failure group (*n* = 29)	Successful group (*n* = 30)	*P*		*q*
Age (years)	32.98 ± 4.50	33.90 ± 5.00	32.10 ± 3.84	0.126		0.736
Body mass index (kg/m²)	21.35 ± 3.14	22.04 ± 3.73	20.68 ± 2.31	0.096		0.736
Times of IVF (*n*)	1.93 ± 1.36	2.07 ± 1.56	1.80 ± 1.16	0.453		0.736
Duration of infertility (years)	3.98 ± 2.87	3.59 ± 2.47	4.37 ± 3.19	0.300		0.736
Baseline estradiol (pg/mL)	37.53 ± 13.59	39.05 ± 14.52	36.05 ± 12.69	0.401		0.736
Baseline luteinizing hormone (IU/L)	4.00 ± 2.02	3.84 ± 1.52	4.15 ± 2.43	0.553		0.745
Baseline follicle-stimulating hormone (IU/L)	6.68 ± 1.86	6.56 ± 1.85	6.80 ± 1.89	0.627		0.768
Baseline progesterone (ng/mL)	0.54 ± 0.35	0.57 ± 0.34	0.50 ± 0.37	0.440		0.736
Baseline testosterone (ng/mL)	1.37 ± 0.62	1.42 ± 0.68	1.31 ± 0.56	0.522		0.736
Baseline prolactin (mIU/L)	344.28 ± 196.99	297.24 ± 145.68	388.19 ± 228.87	0.079		0.736
Baseline anti-Müllerian hormone (ng/mL)	4.77 ± 3.45	4.46 ± 3.73	5.07 ± 3.21	0.506		0.736
Quantity of embryos transferred (*n*)	1.63 ± 0.49	1.69 ± 0.47	1.57 ± 0.54	0.337		0.736
Endometrial thickness (mm)	9.57 ± 1.46	9.65 ± 1.63	9.49 ± 1.31	0.669		0.736
Estradiol before FET (pg/mL)	615.38 ± 1027.51	580.34 ± 733.26	389.85 ± 464.11	0.262		0.768
Progesterone before FET (ng/mL)	23.42 ± 12.31	23.52 ± 12.28	23.32 ± 12.58	0.953		0.985
Day 3 estradiol (pg/mL)	415.29 ± 440.83	415.66 ± 449.95	414.93 ± 439.54	0.995		0.995
Day 3 progesterone (ng/mL)	26.41 ± 9.63	25.84 ± 10.65	26.95 ± 8.64	0.662		0.768
Day 9 Estradiol (pg/mL)	503.31 ± 625.94	561.76 ± 707.96	446.80 ± 541.25	0.485		0.735
Day 9 progesterone (ng/mL)	28.18 ± 9.89	27.93 ± 9.97	28.42 ± 9.97	0.851		0.910
Day 9 human chorionic gonadotropin (HCG, mIU/mL)	100.40 ± 121.99	13.21 ± 23.60	184.68 ± 119.47	<0.001		0.031
Alanine aminotransferase (U/L)	18.51 ± 12.83	20.74 ± 15.05	16.46 ± 10.07	0.203		0.736
Aspartate aminotransferase (U/L)	20.80 ± 12.96	22.51 ± 17.73	19.15 ± 5.17	0.325		0.736
Triglycerides (mmol/L)	1.58 ± 1.83	1.92 ± 2.46	1.23 ± 0.66	0.162		0.736
Total cholesterol (mmol/L)	4.82 ± 0.69	4.89 ± 0.64	4.74 ± 0.75	0.406		0.736
Fasting glucose (mmol/L)	4.88 ± 0.51	4.91 ± 0.52	4.84 ± 0.50	0.617		0.768
High-density lipoprotein cholesterol (mmol/L)	1.38 ± 0.34	1.33 ± 0.29	1.44 ± 0.38	0.232		0.736
Low-density lipoprotein cholesterol (mmol/L)	2.95 ± 0.58	3.07 ± 0.61	2.82 ± 0.54	0.114		0.736
Apolipoprotein A1 (g/L)	1.42 ± 0.27	1.40 ± 0.23	1.45 ± 0.31	0.508		0.736
Apolipoprotein B (g/L)	0.86 ± 0.21	0.89 ± 0.22	0.83 ± 0.18	0.247		0.736
Lipoprotein (a) (mg/L)	200.13 ± 227.59	207.36 ± 242.14	192.35 ± 215.18	0.808		0.895
Homocysteine (µmol/L)	10.14 ± 3.07	10.53 ± 3.78	9.72 ± 2.04	0.327		0.736

^*a*^ Baseline means the baseline hormone levels on the third day of the last menstrual cycle prior to FET. Day 3 means 3 days after FET, and day 9 means 9 days after FET.

Additionally, no significant differences were observed in the characteristics of the transferred embryos, including high-quality embryo rate (40, 41) and blastocyst rate (*P* > 0.05) (Table 3).

**TABLE 3:** Description of characteristics of the transferred embryos^*a*^

Characteristic	Failure group	Successful group	*P*
High-quality embryo rate	30 out of 49	35 out of 47	0.165
Blastocyst rate	17 out of 49	21 out of 47	0.317

^*a*^ The high-quality embryo rate is calculated as the number of high-quality embryos divided by the total number of embryos (high-quality embryos + non-high-quality embryos). The blastocyst rate is defined as the number of blastocysts divided by the total number of embryos (blastocysts + cleavage-stage embryos).

### Characterizing the composition of gut, oral, and vaginal microbiota at different times

#### Gut microbiota

At the phylum and genus levels, the dominant gut microbiota composition across GA, GB, and GC is provided in the supplemental material (Fig. S3A and B). LEfSe analysis revealed that *Stenotrophomonas*, *Xanthomonadales*, and *Xanthomonadaceae* were significantly enriched in GA compared to GB and GC (*P* < 0.05) (Fig. S3C).

#### Oral microbiota

At the phylum and genus levels, the dominant oral microbiota composition across OA, OB, and OC is provided in the supplemental material (Fig. S4A,B). LEfSe analysis revealed significant variations in oral microbiota during FET (Fig. S4C).

#### Vaginal microbiota

At the phylum and genus levels, the dominant vaginal microbiota composition across VA, VB, and VC is provided in the supplemental material (Fig. S5A and B). LEfSe analysis revealed variations in vaginal microbiota during FET, though these changes were less pronounced compared to the oral microbiota (Fig. S5C).

#### Alpha-diversity differences

Alpha-diversity analysis (Chao1, Shannon, and Simpson indices) showed no significant differences in gut microbiota richness or diversity among GA, GB, and GC. Oral microbiota diversity was significantly higher in OB and OC compared to OA (*P* < 0.05), while richness remained unchanged. In vaginal microbiota, richness was significantly lower in VB than in VA and VC, but diversity showed no significant difference (Fig. S6).

#### Beta diversity differences

NMDS and principal coordinate analysis (PCoA) were used to analyze the beta diversity of gut, oral, and vaginal microbiota during FET. The beta diversity of the gut, oral, and vaginal microbiota showed differences. However, the gut microbiota in the different groups were similar, as were the vaginal and oral microbiota, indicating that the FET process did not significantly alter beta diversity in these sites (Fig. S7).

#### Network diagram of the correlation of differential microbiota

Co-occurrence networks of the core genera (top 50) were constructed for the gut, oral, and vaginal microbiota within each group. The diagram reveals intricate interrelationships among bacterial genera, highlighting their correlations. In GA, GB, and GC, the number of nodes, edges, average degree, density, and clustering coefficient showed an overall declining trend, suggesting a reduction in network complexity over the FET process. In OB and OC, the nodes, edges, and average degree were lower compared to OA, indicating a shift in microbial interactions after FET. Similarly, in VC, the numbers of edges, average degree, density, and clustering coefficient were lower than in VA and VB, reflecting a reduction in network complexity within the vaginal microbiota as well (Fig. S8 and S9).

### Characterizing the composition of gut, oral, and vaginal microbiota in different groups and different times

#### Gut microbiota

**Fig 2:**
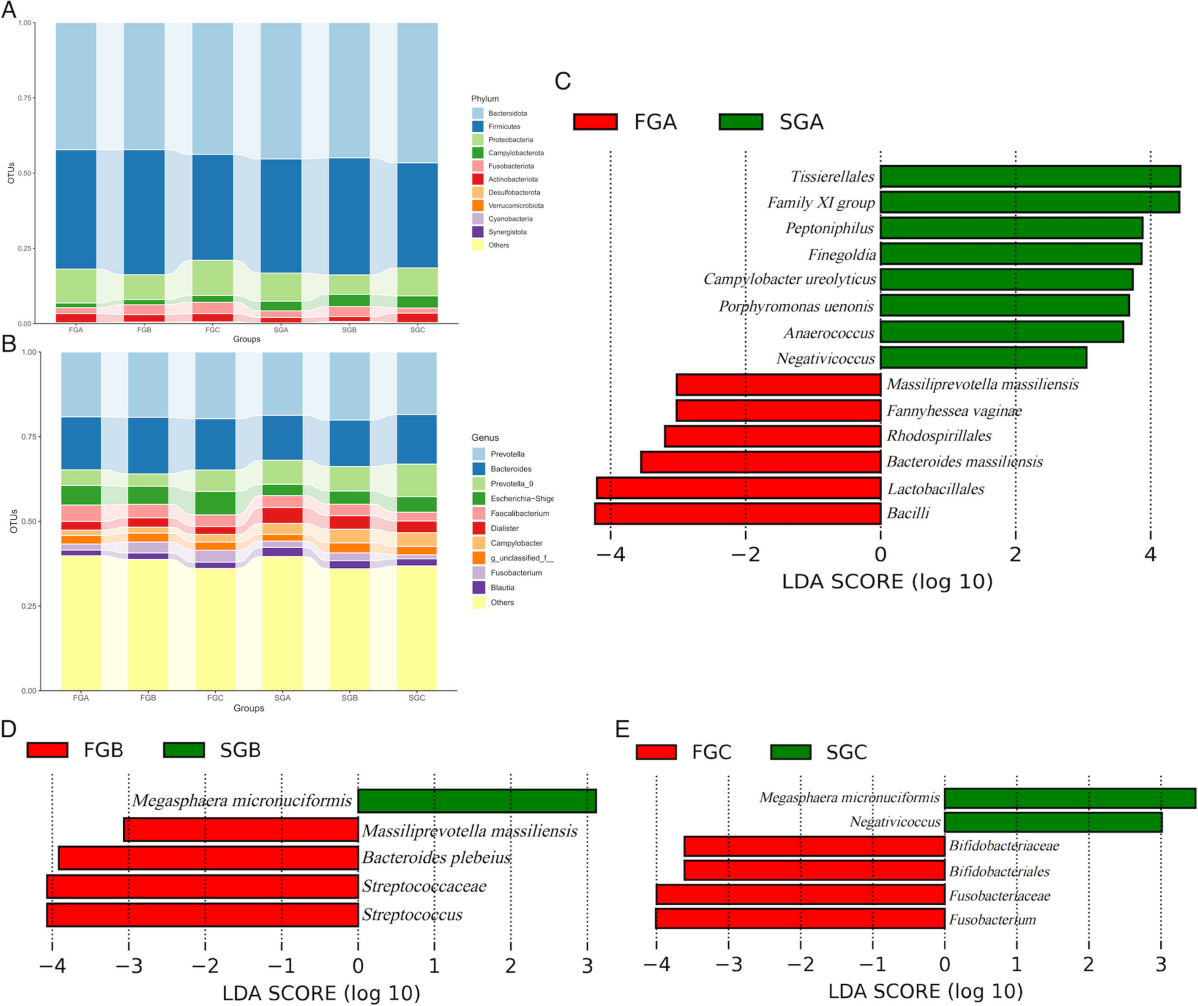
(A) Bar chart of gut microbiota in each group during the FET process at the phylum level. (B) Bar chart of gut microbiota in each group during the FET process at the genus level. (C–E) Bar plots showing the different taxa with a linear discriminant analysis (LDA) score of >3 and *P* < 0.05. The distance between each point represents the degree of difference in the microbiome of each sample. The length of the bars represents the magnitude of the impact of differential species.

At the phylum level, *Bacteroidota* (42.24%, 42.21%, and 43.78%, respectively), *Firmicutes* (39.66%, 41.55%, and 35.19%, respectively), and *Proteobacteria* (11.28%, 8.25%, and 11.71%, respectively) were the three most common components of gut microbiota in FGA, FGB, and FGC. Similarly, *Bacteroidota* (45.31%, 44.94%, and 46.59%, respectively), *Firmicutes* (37.90%, 38.89%, and 34.93%, respectively), and *Proteobacteria* (9.36%, 6.49%, and 9.29%, respectively) were the three most common components of gut microbiota in SGA, SGB, and SGC (Fig. 2A).

At the genus level, the five most abundant components of the gut microbiota in FGA, FGB, and FGC were *Prevotella* (19.14%, 19.28%, and 19.70%, respectively), *Bacteroides* (15.66%, 16.72%, and 15.16%, respectively), *Escherichia*–*Shigella* (5.79%, 5.34%, and 6.92%, respectively), *Faecalibacterium* (4.79%, 3.94%, and 3.44%, respectively), and *Prevotella* subgroup 9 (4.62%, 3.63%, and 6.30%, respectively). In SGA, the five most abundant genera were *Prevotella* (18.71%), *Bacteroides* (13.27%), *Prevotella* subgroup 9 (7.08%), *Dialister* (4.74%), and *Faecalibacterium* (3.50%). In SGB, the five most abundant components were *Prevotella* (20.10%), *Bacteroides* (13.73%), *Prevotella* subgroup 9 (7.24%), *Campylobacter* (4.02%), and *Dialister* (4.01%). In SGC, the five most abundant components were *Prevotella* (18.44%), *Bacteroides* (14.66%), *Prevotella* subgroup 9 (9.60%), *Escherichia*–*Shigella* (4.61%), and *Campylobacter* (4.03%) (Fig. 2B).

LEfSe analysis revealed significant differences in gut microbiota between the failure and successful groups at various time points. Before FET, *Tissierellales*, *Family XI* group, *Peptoniphilus*, *Finegoldia*, *Campylobacter ureolyticus*, *Porphyromonas uenonis*, *Anaerococcus*, and *Negaticicoccus* were significantly more abundant in SGA than in FGA (*P* < 0.05). Conversely, *Massiliprevotella massiliensis*, *Fannyhessea vaginae*, *Rhodospirillales*, *Bacteroides massiliensis*, *Lactobacillales*, and *Bacilli* were significantly more abundant in FGA than in SGA (*P* < 0.05) (Fig. 2C).

*Megasphaera micronuciformis* was significantly more abundant in SGB than in FGB (*P* < 0.05). In contrast, *Massiliprevotella massiliensis*, *Bacteroides plebeius*, *Streptococcaceae*, and *Streptococcus* were significantly more abundant in FGB than in SGB (*P* < 0.05) (Fig. 2D).

*Megasphaera micronuciformis* and *Negativicoccus* were significantly more abundant in SGC than in FGC (*P* < 0.05). Meanwhile, *Bifidobacteriaceae*, *Bifidobacteriales*, *Fusobacteriaceae*, and *Fusobacterium* were significantly more abundant in FGC than in SGC (*P* < 0.05) (Fig. 2E).

#### Oral microbiota

**Fig 3:**
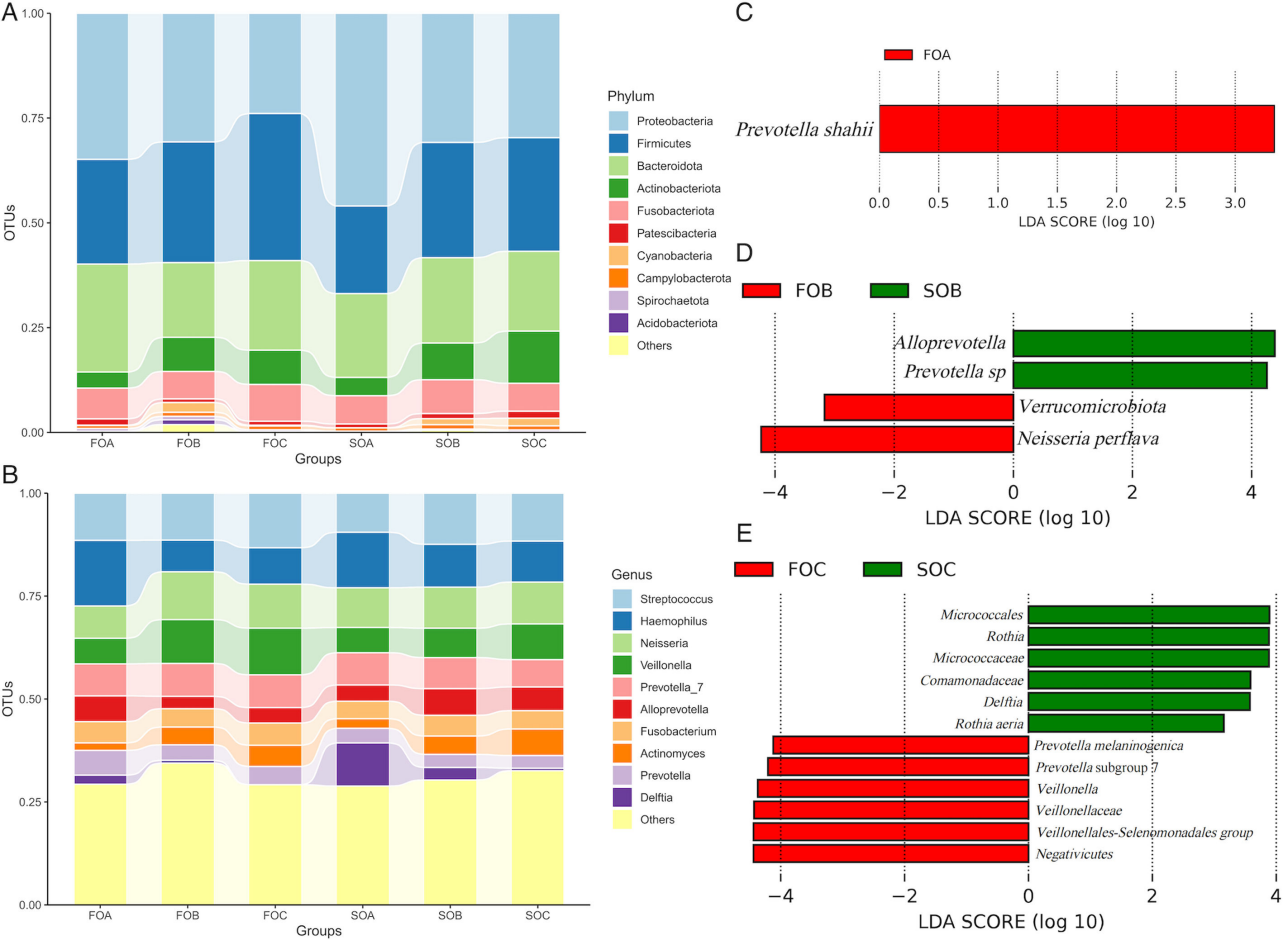
(A) Bar chart of oral microbiota in each group during the FET process at the phylum level. (B) Bar chart of oral microbiota in each group during the FET process at the genus level. (C–E) Bar plot showing the different taxa with an LDA score of >3 and *P* < 0.05. The distance between each point represents the degree of difference in the microbiome of each sample. The length of the bars represents the magnitude of the impact of differential species.

At the phylum level, *Proteobacteria* (36.47%, 29.68%, and 25.33%, respectively), *Firmicutes* (24.06%, 30.66%, and 32.60%, respectively), and *Bacteroidota* (24.30%, 17.63%, and 22.23%, respectively) were the three most common components of oral microbiota in FOA, FOB, and FOC. Similarly, *Proteobacteria* (47.38%, 31.84%, and 29.85%, respectively), *Firmicutes* (20.72%, 25.31%, and 27.42%, respectively), and *Bacteroidota* (19.87%, 21.23%, and 17.53%, respectively) were the three most common components of oral microbiota in SOA, SOB, and SOC (Fig. 3A).

At the genus level, the five most abundant components of the oral microbiota were *Haemophilus* (16.41%, 9.60% and 10.12% respectively), *Streptococcus* (10.77%, 13.02% and 11.73%, respectively), *Neisseria* (9.03%, 10.00% and 11.29% respectively), *Veillonella* (6.92%, 9.63% and 11.95%, respectively), and *Prevotella* subgroup 7 (8.41%, 7.63% and 8.34%) in FOA, FOB, and FOC. In SOA, the five most abundant genera were *Haemophilus* (12.40%), *Delftia* (12.24%), *Streptococcus* (9.62%), *Neisseria* (8.95%), and *Prevotella* subgroup 7 (7.22%). In SOB, the five most abundant genera were *Neisseria* (11.10%), *Streptococcus* (11.06%), *Haemophilus* (9.29%), *Prevotella* subgroup 7 (7.76%), and *Alloprevotella* (7.65%). In SOC, the five most abundant genera were *Streptococcus* (12.70%), *Neisseria* (9.37%), *Haemophilus* (9.02%), *Veillonella* (7.35%), and *Actinomyces* (6.19%) (Fig. 3B).

LEfSe analysis revealed significant differences in the oral microbiota between the failure and successful groups at different periods. Before FET, *Prevotella shahii* was significantly more abundant in FOA than in SOA (*P* < 0.05) (Fig. 3C).

*Alloprevotella* and *Prevotella* spp. were significantly more abundant in SOB than in FOB (*P* < 0.05). *Verrucomicrobiota* and *Neisseria perflava* were significantly more abundant in FOB than in SOB (*P* < 0.05) (Fig. 3D).

*Prevotella melaninogenica*, *Prevotella* subgroup 7, *Veillonella*, *Veillonellaceae*, *Veillonellales-Selenomonadales* group*,* and *Negativicutes* were significantly more abundant in FOC than in SOC, while *Comamonadaceae*, *Micrococcaceae*, *Micrococcales*, *Rothia*, *Delftia*, and *Rothia aeria* were significantly more abundant in SOC than in FOC (*P* < 0.05) (Fig. 3E).

#### Vaginal microbiota

**Fig 4:**
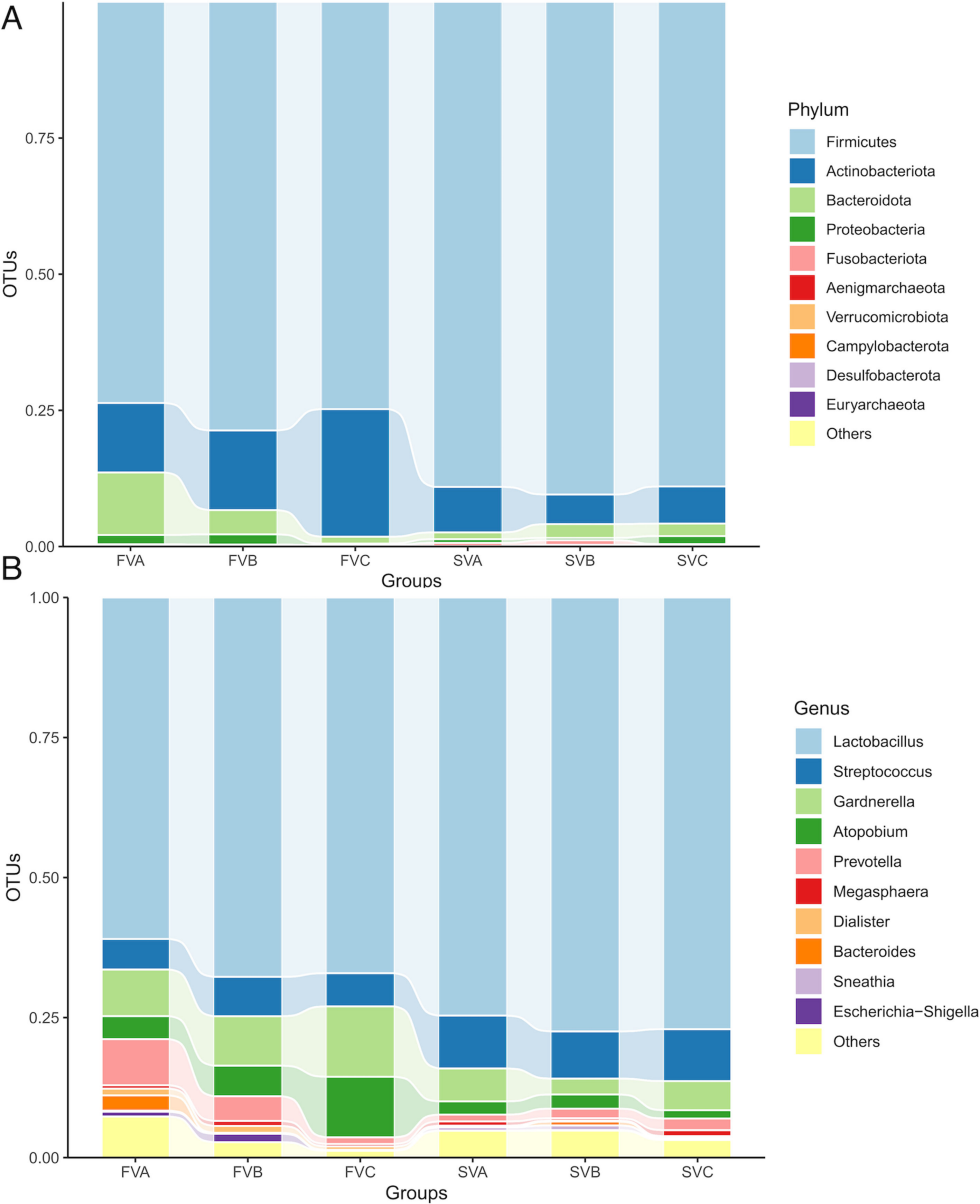
(A) Bar chart of vaginal microbiota in each group during the FET process at the phylum level. (B) Bar chart of vaginal microbiota in each group during the FET process at the genus level. OTU, operational taxonomic unit.

At the phylum level, *Firmicutes* (78.13%, 82.91%, and 79.96%, respectively), *Actinobacteriota* (12.00%, 10.65%, and 17.25%, respectively), and *Bacteroidota* (8.32%, 4.32%, and 1.16%, respectively) were the three most common components of vaginal microbiota in FVA, FVB, and FVC. Similarly, *Firmicutes* (88.83%, 89.47%, and 87.70%, respectively), *Actinobacteriota* (7.92%, 6.88%, and 8.42%, respectively), and *Bacteroidota* (1.56%, 2.14%, and 2.67%, respectively) were the three most common components of vaginal microbiota in SVA, SVB, and SVC (Fig. 4A).

At the genus level, the five most abundant components of vaginal microbiota in FVA, FVB, and FVC were *Lactobacillus* (65.28%, 69.75%, and 72.10%, respectively), *Gardnerella* (8.38%, 6.41%, and 9.37%, respectively), *Streptococcus* (7.38%, 8.49%, and 6.41%, respectively), *Prevotella* (5.96%, 3.26%, and 0.91%, respectively), and *Atopobium* (3.37%, 3.95%, and 7.83%). In SVA, SVB, and SVC, the five most abundant genera were *Lactobacillus* (74.13%, 78.13%, and 74.79%, respectively), *Streptococcus* (8.63%, 7.36%, and 9.71%, respectively), *Gardnerella* (5.15%, 3.57%, and 6.38%, respectively), *Atopobium* (2.62%, 3.23%, and 1.87%), and *Prevotella* (1.54%, 2.13%, and 2.60%) (Fig. 4B).

LEfSe analysis was also used to reveal significant differences in vaginal microbiota between the failure and successful groups at different time points. Surprisingly, no significant differences were found in the vaginal microbiota between the two groups.

#### Alpha-diversity differences

Chao1, Shannon, and Simpson indices were used for alpha-diversity analysis. There were no significant differences in richness (Chao1) or diversity (Shannon and Simpson) among gut microbiota in FGA and SGA, FGB and SGB, or FGC and SGC.

For oral microbiota, the alpha-diversity results showed that the Shannon index was lower in SOA than in SOB and SOC. Furthermore, the richness and diversity of SOA were significantly lower than those of FOB and FOC.

**Fig 5:**
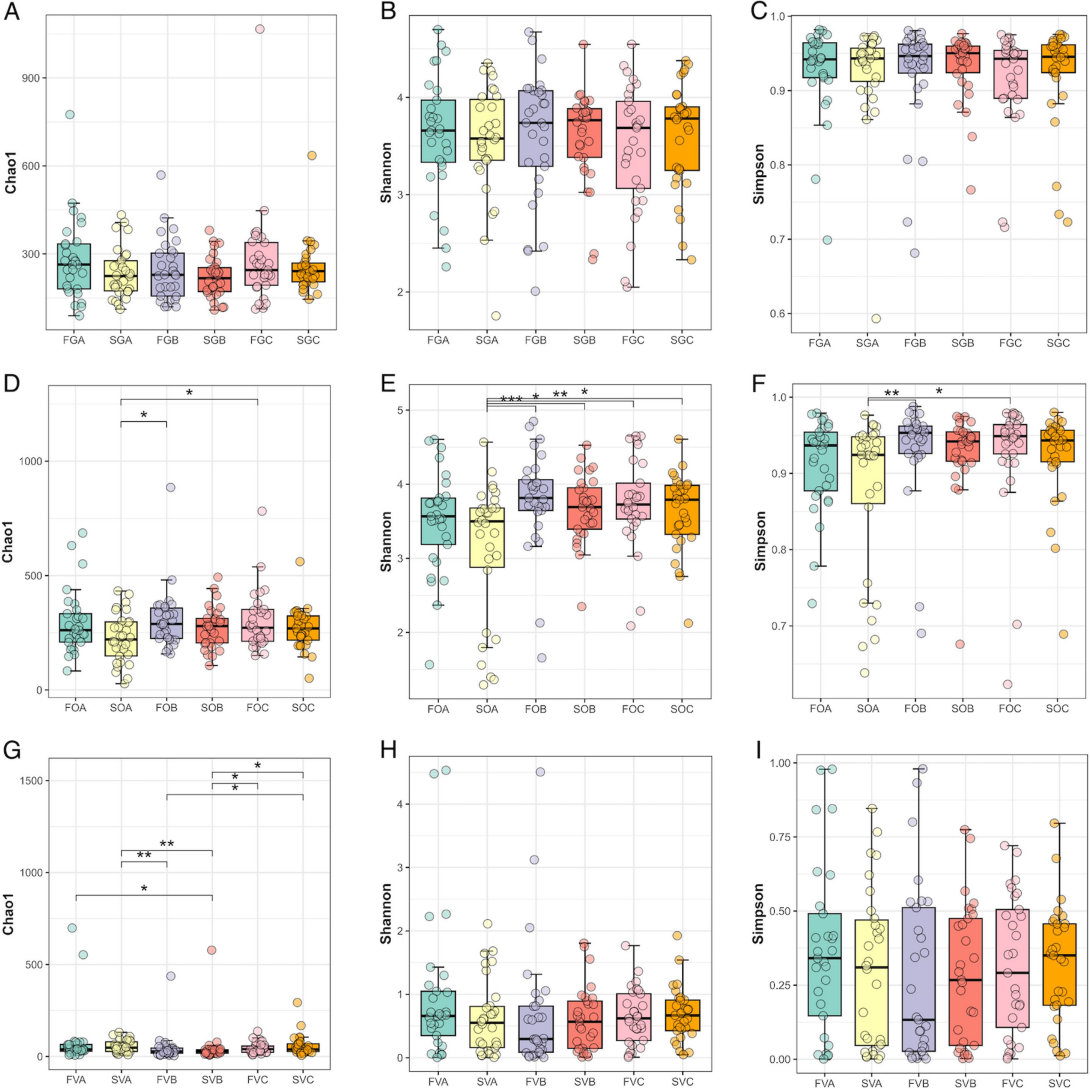
Alpha diversity of each microbiota during the FET process is shown as a boxplot (mean ± SD). (A) Chao1 index of gut microbiota in each group. (B) Shannon index of gut microbiota in each group. (C) Simpson index of gut microbiota in each group. (D) Chao1 index of oral microbiota in each group. (E) Shannon index of oral microbiota in each group. (F) Simpson index of oral microbiota in each group. (G) Chao1 index of vaginal microbiota in each group. (H) Shannon index of vaginal microbiota in each group. (I) Simpson index of vaginal microbiota in each group.

In vaginal microbiota, the richness in SVB was lower than that in SVA and SVC (Fig. 5).

#### Beta diversity differences

**Fig 6:**
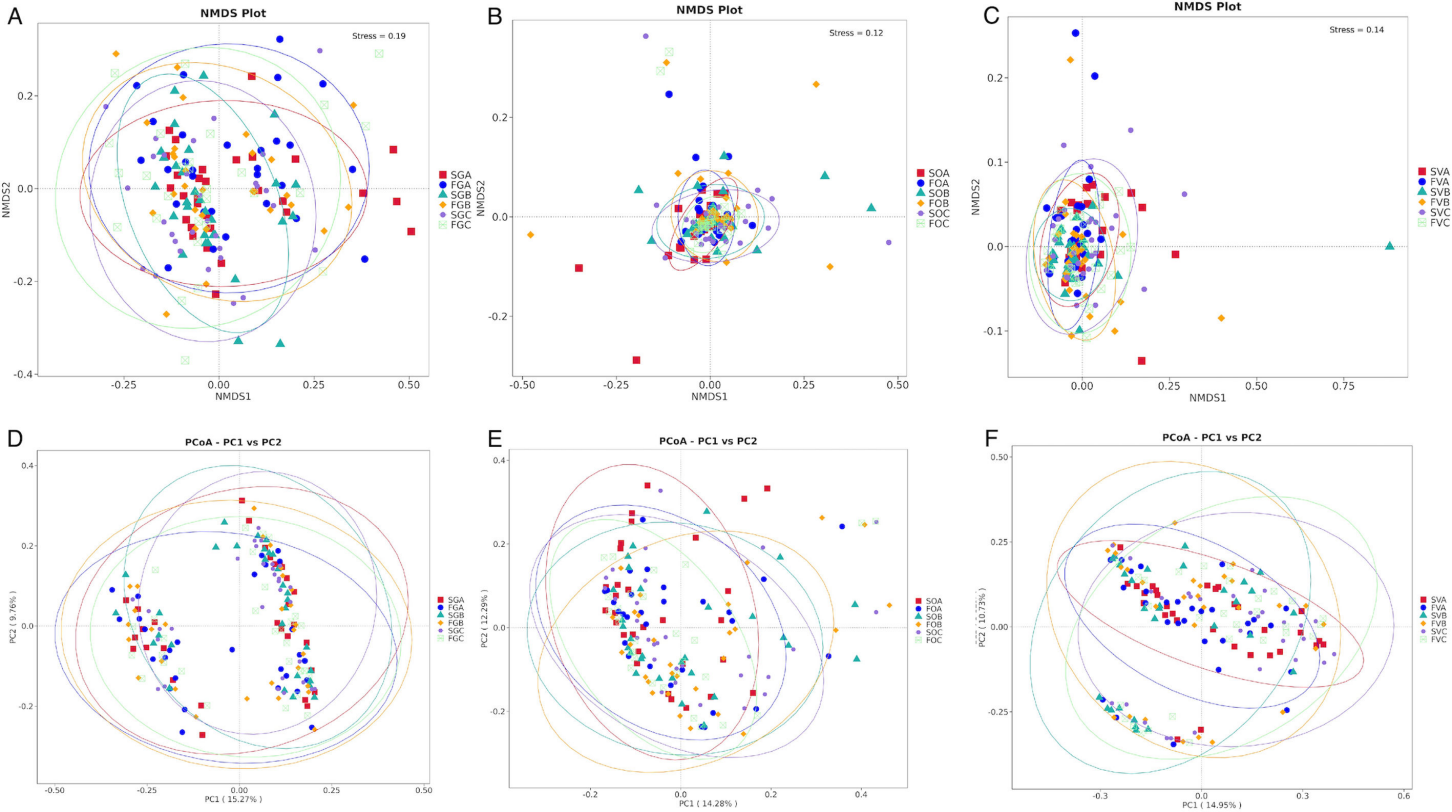
Beta diversity (as assessed by the unweighted UniFrac NMDS and PCoA) of samples in each group. The distance between each point represents the degree of difference in the microbiome of each sample. (A) NMDS of gut microbiota. Stress value (0–1) is a measure of the error between the original distance and the low-dimensional spatial distance obtained by NMDS. The lower stress value (usually <0.05) indicates a very good fit. (B) NMDS of oral microbiota. (C) NMDS of vaginal microbiota. (D) PCoA of gut microbiota. (E) PCoA of oral microbiota. (F) PCoA of vaginal microbiota.

NMDS and PCoA were used to analyze the beta diversity of gut, oral, and vaginal microbiota during FET. The beta-diversity analysis revealed that the gut, vaginal, and oral microbiota were similar across different groups and time periods (Fig. 6).

### Network diagram of the correlation of differential microbiota

#### Gut microbiota topological properties

**Fig 7:**
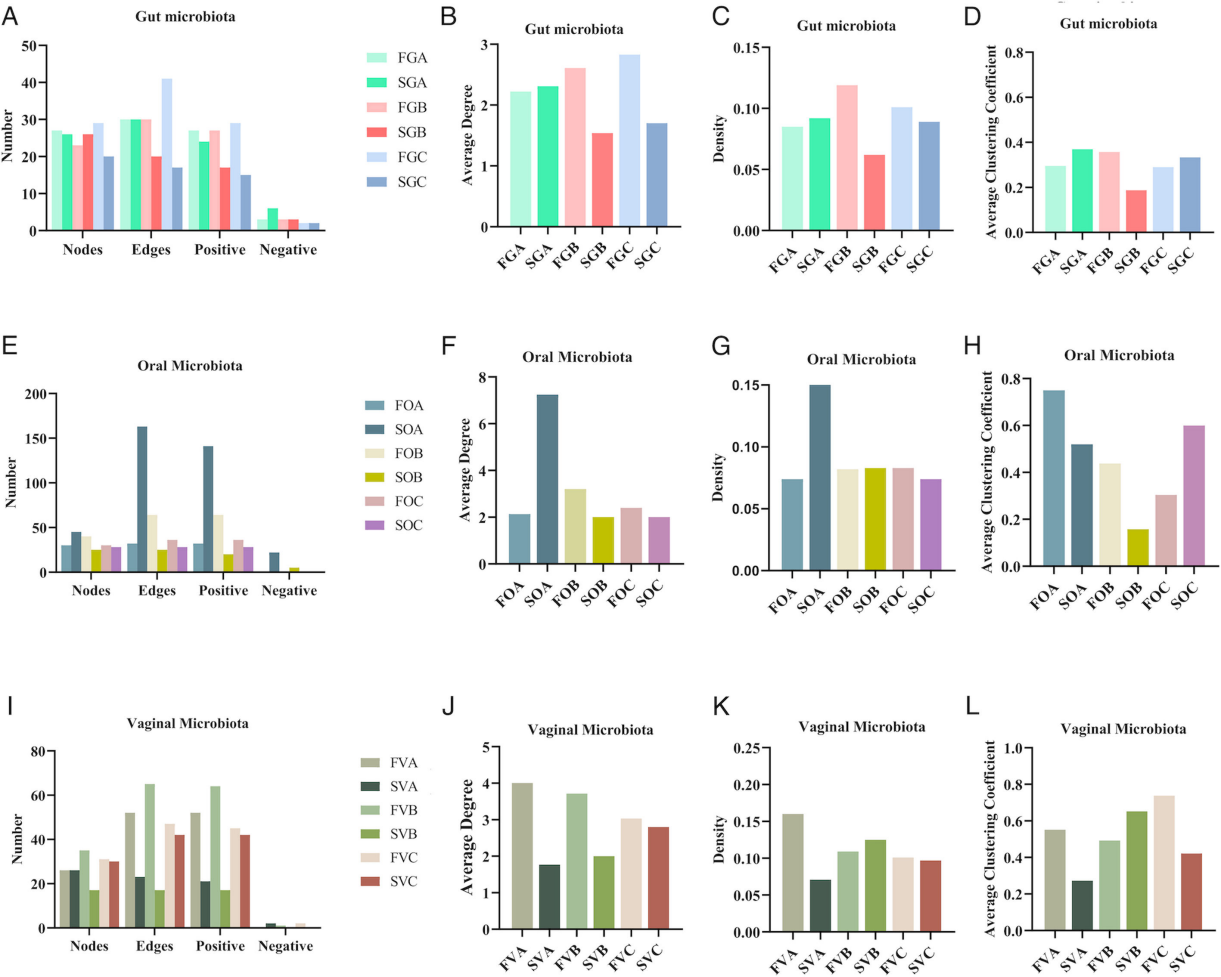
Topological properties of microbial networks. (A) Nodes, edges, and positive and negative correlations of gut microbiota networks. (B) Average degree of gut microbiota networks. (C) Density of gut microbiota networks. (D) Average clustering coefficient of gut microbiota networks. (E) Nodes, edges, and positive and negative correlations of oral microbiota networks. (F) Average degree of oral microbiota networks. (G) Density of oral microbiota networks. (H) Average clustering coefficient of oral microbiota networks. (I) Nodes, edges, and positive and negative correlations of vaginal microbiota networks. (J) Average degree of vaginal microbiota networks. (K) Density of vaginal microbiota networks. (L) Average clustering coefficient of vaginal microbiota networks.

In the gut microbiota, the failure groups (FGB and FGC) consistently exhibited higher network complexity compared to the success groups (SGB and SGC) after FET. For instance, the FGB group (failure, 3 days post-FET) had 23 nodes and 30 edges compared to the SGB group, which had 26 nodes and 20 edges with lower average degree and density. By day 9 post-FET, the FGC group had 29 nodes and 41 edges, while the SGC group had only 20 nodes and 17 edges with lower average degree and density (Fig. 7A through D; Fig. S10A).

#### Oral microbiota topological properties

In the oral microbiota, the differences in network complexity between the success and failure groups were more interesting before and after FET. For example, the SOA group (success, day before FET) had 45 nodes and 163 edges, with an average degree of 7.244 and density of 0.165, whereas the FOA group (failure, day before FET) had 30 nodes and 32 edges, with an average degree of 2.133 and density of 0.074. By 3 days post-FET, the FOB group (failure) had 43 nodes and 64 edges, while the SOB group (success) had 25 nodes and 25 edges with lower average degree. On day 9 post-FET, the FOC group (failure) exhibited higher complexity, with 30 nodes and 36 edges, compared to the SOC group (success), which had 28 nodes and 28 edges with lower average degree and density (Fig. 7E through H; Fig. S10B).

#### Vaginal microbiota topological properties

In the vaginal microbiota, the failure groups (FVA, FVB, and FVC) exhibited greater network complexity compared to the success groups (SVA, SVB, SVC). For example, the FVA group had 26 nodes and 52 edges, with an average degree of 4.000, while the SVA group had 26 nodes and 23 edges, with an average degree of 1.769. By day 3 post-FET, the FVB group had 35 nodes and 65 edges, whereas the SVB group had 17 nodes and 17 edges. On day 9 post-FET, the FVC group had 31 nodes and 47 edges, with an average degree of 3.032, while the SVC group had 30 nodes and 42 edges, with an average degree of 2.800 (Fig. 7I through L; Fig. S10C).

#### Correlation between microbiota and clinical outcomes

In our study, heatmaps were used to explore the correlations between the top 50 most abundant genera and clinical parameters. To refine this analysis, we incorporated the LEfSe results to focus on differentially abundant genera and further explore their potential associations with clinical outcomes.

#### Gut microbiota

**Fig 8:**
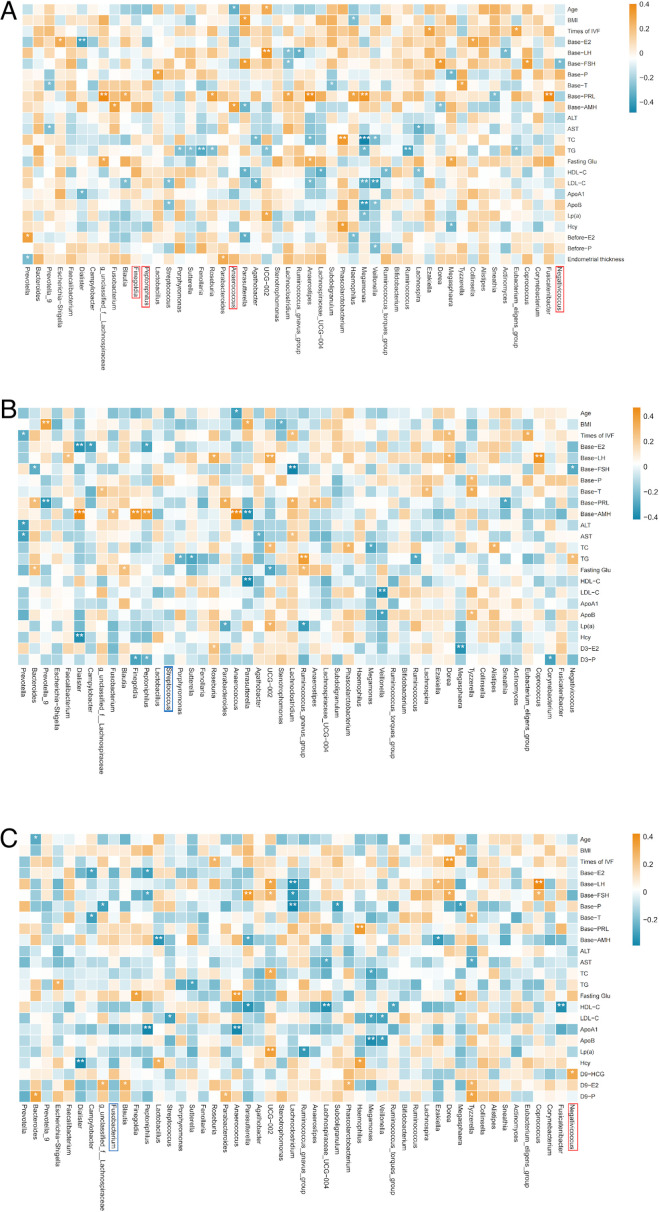
Spearman correlation heatmap of gut microbiota and clinical outcomes in different groups. (A) Correlation between gut microbiota before FET and clinical results. (B) Correlation between gut microbiota on the third day post-FET and clinical results. (C) Correlation between gut microbiota on the ninth day post-FET and clinical results. Red squares represent positive correlation; blue squares represent negative correlation. Red frames represent more abundance evaluated by LEfSe in the successful group. Blue frames represent more abundance evaluated by LEfSe in the failure group. *P < 0.05, **P < 0.01, ***P < 0.001. ALT, alanine aminotransferase; AST, aspartate aminotransferase; TC, total cholesterol; TG, triglyceride.

A Spearman correlation heatmap was used to show the correlation between gut microbiota at the genus level (top 50) and clinical characteristics at corresponding time points. Combined with the LEfSe results, specific bacterial genera were found to be correlated with clinical characteristics. Before FET, *Anaerococcus*, which was more abundant in the successful group, showed a positive correlation with baseline anti-Müllerian hormone (AMH) and a negative correlation with the age of patients. Additionally, *Negativicoccus*, which was more abundant in the successful group, had a negative correlation with baseline follicle-stimulating hormone (FSH) (Fig. 8A).

On the third day after FET, we did not find any correlation between differential bacterial genera and clinical characteristics (Fig. 8B)

On the ninth day after FET, *Negativicoccus*, more abundant in the successful group, had a positive correlation with HCG (Fig. 8C).

#### Oral microbiota

**Fig 9:**
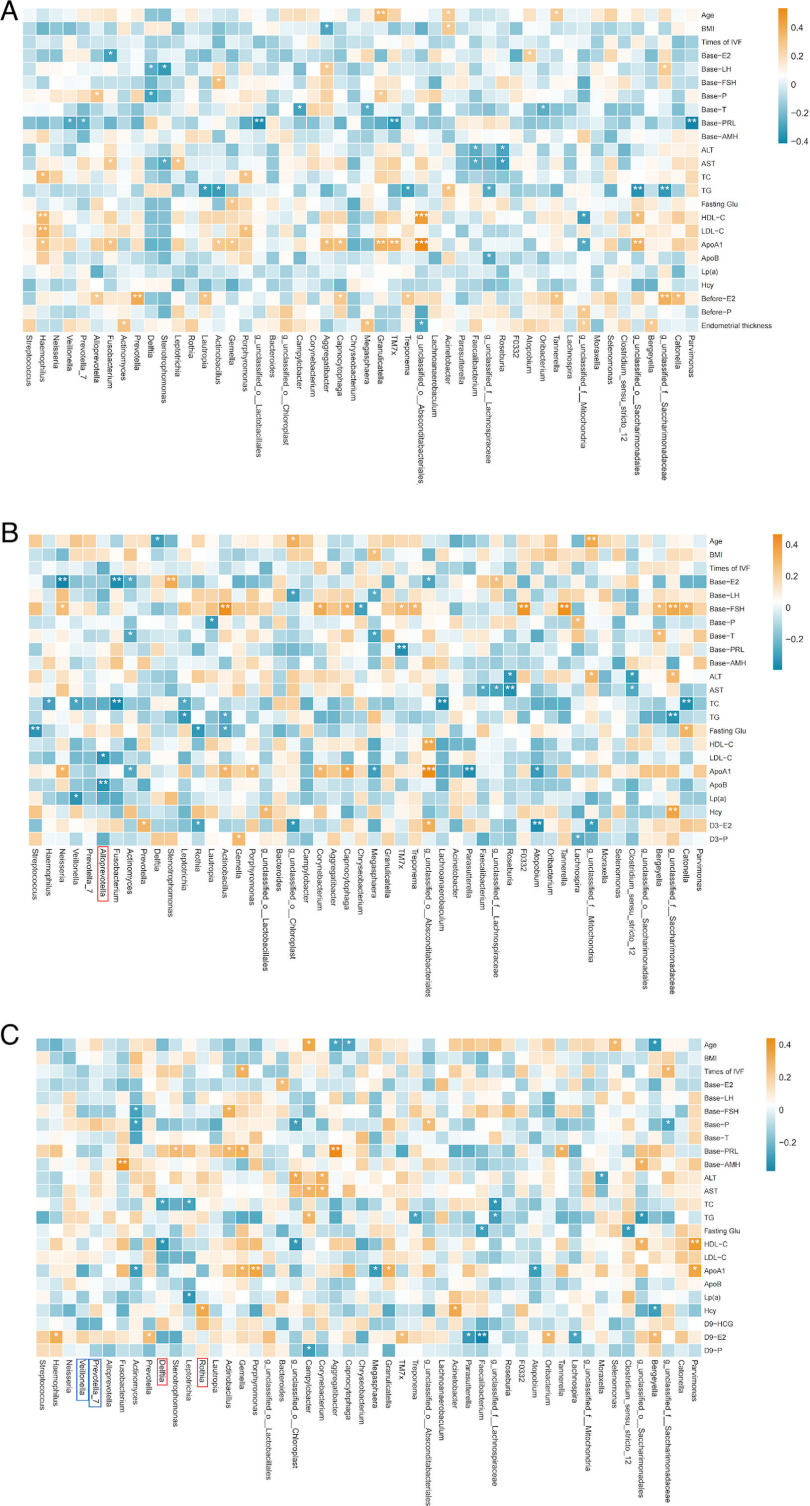
Spearman correlation heatmap of oral microbiota and clinical outcomes in different groups. (A) Correlation between oral microbiota before FET and clinical results. (B) Correlation between oral microbiota on the third day post-FET and clinical results. (C) Correlation between oral microbiota on the ninth day post-FET and clinical results. Red squares represent positive correlation; blue squares represent negative correlation. Red frames represent more abundant evaluated by LEfSe in the successful group. Blue frames represent more abundance evaluated by LEfSe in the failure group. *P < 0.05, **P < 0.01, ***P < 0.001.

We also constructed a Spearman correlation heatmap to evaluate the correlation between oral microbiota at the genus level (top 50) and clinical characteristics at corresponding time points. However, combined with the LEfSe results, on the third day post-FET, *Allprevotella*, which was more abundant in the successful group, showed negative correlation with low-density lipoprotein cholesterol and ApoB. On the ninth day post-FET, *Delftia*, which was more abundant in the successful group, showed a negative correlation with total cholesterol (TC) and high-density lipoprotein cholesterol. Meanwhile, *Rothia*, which was more abundant in the successful group, showed a positive correlation with homocysteine (Fig. 9).

#### Vaginal microbiota

**Fig 10:**
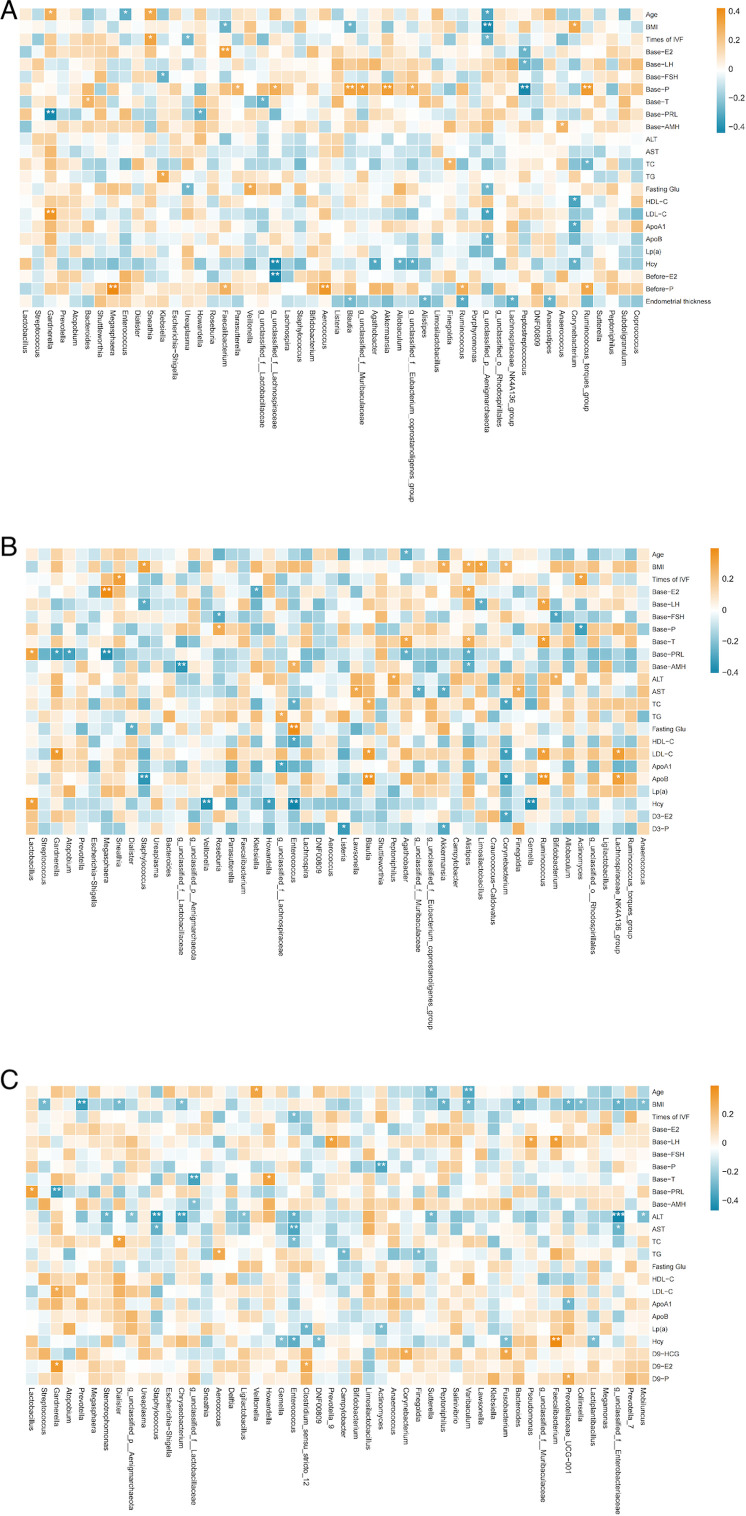
Spearman correlation heatmap of vaginal microbiota and clinical outcomes in different groups. (A) Correlation between vaginal microbiota before FET and clinical results. (B) Correlation between vaginal microbiota on the third day post-FET and clinical results. (C) Correlation between vaginal microbiota on the ninth day post-FET and clinical results. Red squares represent positive correlation; blue squares represent negative correlation. Red frames represent more abundance evaluated by LEfSe in the successful group. Blue frames represent more abundance evaluated by LEfSe in the failure group. *P < 0.05, **P < 0.01, ***P < 0.001.

We also constructed a Spearman correlation heatmap to evaluate the correlation between vaginal microbiota at the genus level (top 50) and clinical characteristics at corresponding time points. We observed that *Lactobacillus* had a positive correlation with baseline prolactin on the third day and ninth day post-FET (Fig. 10).

### Microbiome and metabolites

#### Random forest

**Fig 11:**
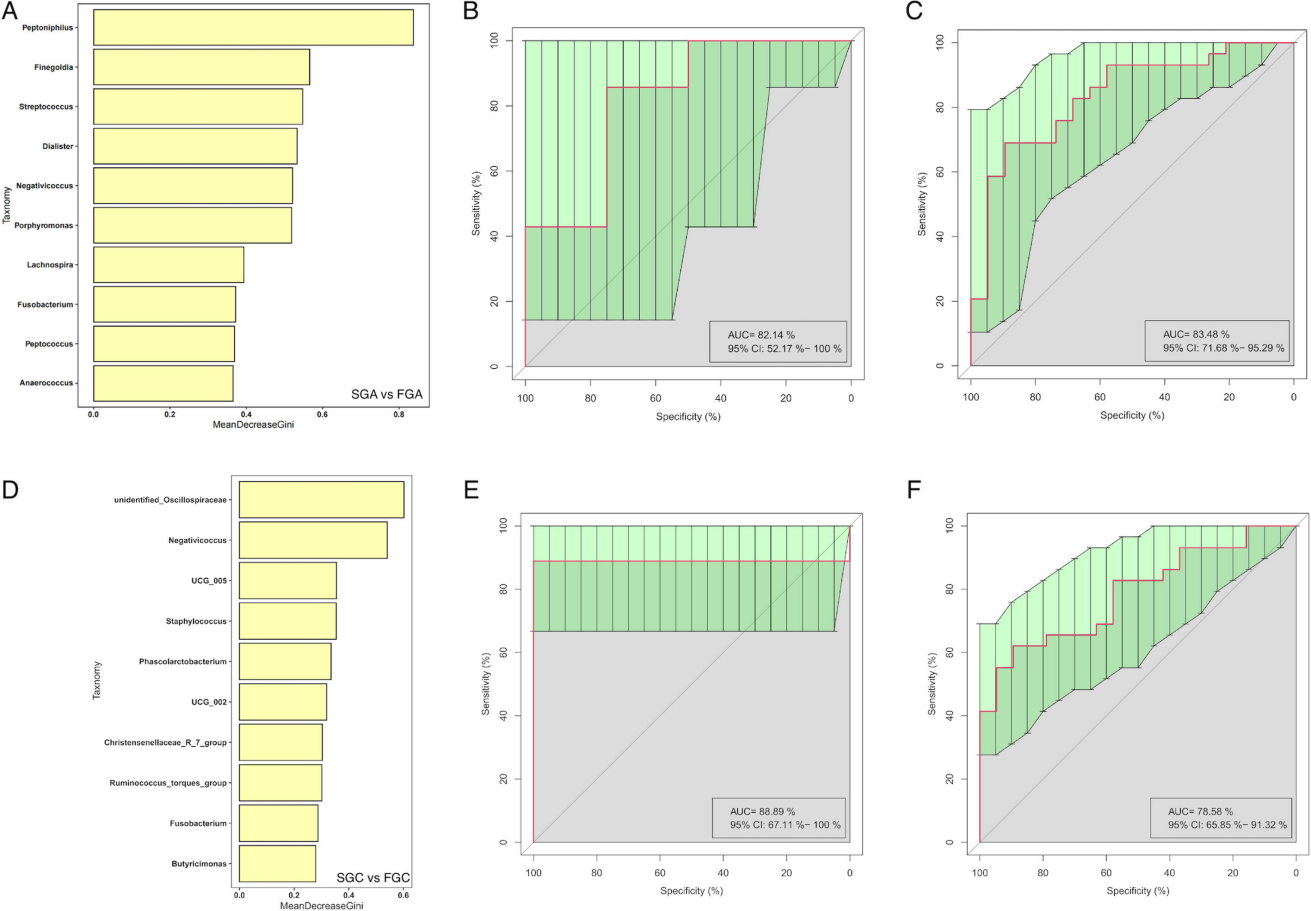
Random forest results for gut microbiota before FET and on the ninth day post-FET. (A) Top 10 gut bacterial genus before FET selected by MeanDecreaseGini. The length of the bar represents the importance that distinguishes the failure group and the successful group. (B) AUC of test set for gut microbiota before FET. (C) AUC of train set for gut microbiota before FET. (D) Top 10 gut bacterial genus on the ninth day post-FET selected by MeanDecreaseGini. (E) AUC of test set for gut microbiota on the ninth day post-FET. (F) AUC of train set for gut microbiota on the ninth day post-FET.

To further understand the relationship between gut microbiota and FET outcomes, a cross-validation curve from the random forest model identified 10 OTU biomarkers for gut microbiota before FET. The ROC curve constructed with the training set showed an AUC of 0.8348 with a 95% CI of 0.7168–0.9529. For the test set, the AUC was 0.8214, with a 95% CI of 0.5217–1.0 (Fig. 11A through C).

We also used the random forest model to identify 10 OTU biomarkers for gut microbiota between the FGC and SGC groups. The ROC curve constructed with the training set showed an AUC of 0.7858 with a 95% CI of 0.6585–0.9132. For the test set, the AUC was 0.8889, with a 95% CI of 0.6711–1.0 (Fig. 11D through F).

#### Difference in serum metabolites between the successful group and the failure group before FET

**Fig 12:**
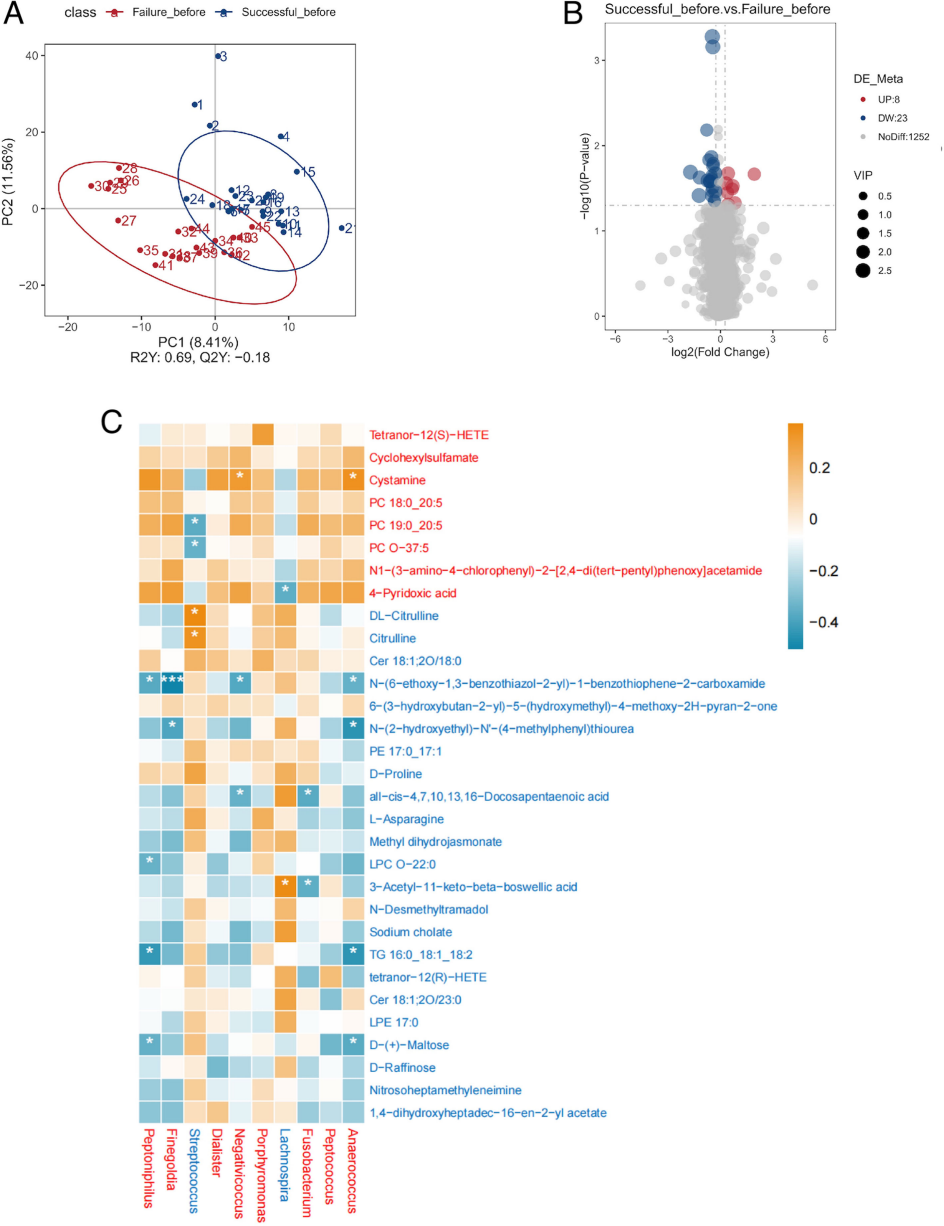
(A) PLS-DA for metabolites before FET in the successful group and the failure group. The distance between each point represents the degree of difference in the metabolites of each sample. (B) Volcano plots were used to filter metabolites of interest based on log2(fold change) and −log10(*P* value) of metabolites. Up (UP) represents having a higher concentration in the successful group. Down (DW) represents having a higher concentration in the failure group. (C) Spearman correlation heatmap of different metabolites and bacterial genus selected by random forest in the failure group and the successful group before FET. Red squares represent positive correlation; blue squares represent negative correlation. Red metabolite or bacterial genus represents more abundance in the successful group. Blue metabolite or bacterial genus represents more abundance evaluated in the failure group. *P < 0.05; **P < 0.01; ***P < 0.001.

PLS-DA was used to investigate the differences in serum metabolites between the successful and failure groups before FET. We combined both positive and negative ionization models. Before FET, the serum metabolite composition was clearly distinguishable between the failure and successful groups. Additionally, a 200-cycle permutation test was performed to identify the best-fitted PLS-DA model. A total of 1,252 differential metabolites were identified between the two groups based on the PLS-DA model’s predictive value (VIP > 1.0) combined with *P* < 0.05. Differential analysis showed that 8 metabolites were significantly more abundant in the successful group, while 23 metabolites were significantly more abundant in the failure group (Fig. 12A and B).

Spearman correlation analysis with Benjamini–Hochberg FDR correction was performed to investigate the relationships between the differential metabolites and the gut microbiota identified by the random forest model. Our results showed that cystamine, which was elevated in the successful group prior to FET, was significantly correlated with more than two differential gut microbiota. Similarly, D-(+)-maltose, triglyceride 16:0_18:1_18:2, N-(2-hydroxyethyl)-N′-(4-methylphenyl)thiourea, 3-acetyl-11-keto-beta-boswellic acid, N-(6-ethoxy-1,3-benzothiazol-2-yl)-1-benzothiophene-2-carboxamide and all*-cis*-4,7,10,13,16-docosapentaenoic acid, which were increased in the failure group before FET, were also correlated with more than two differential gut microbiota (Fig. 12C).

#### Difference in serum metabolites between the successful group and the failure group 9 days post-FET

**Fig 13:**
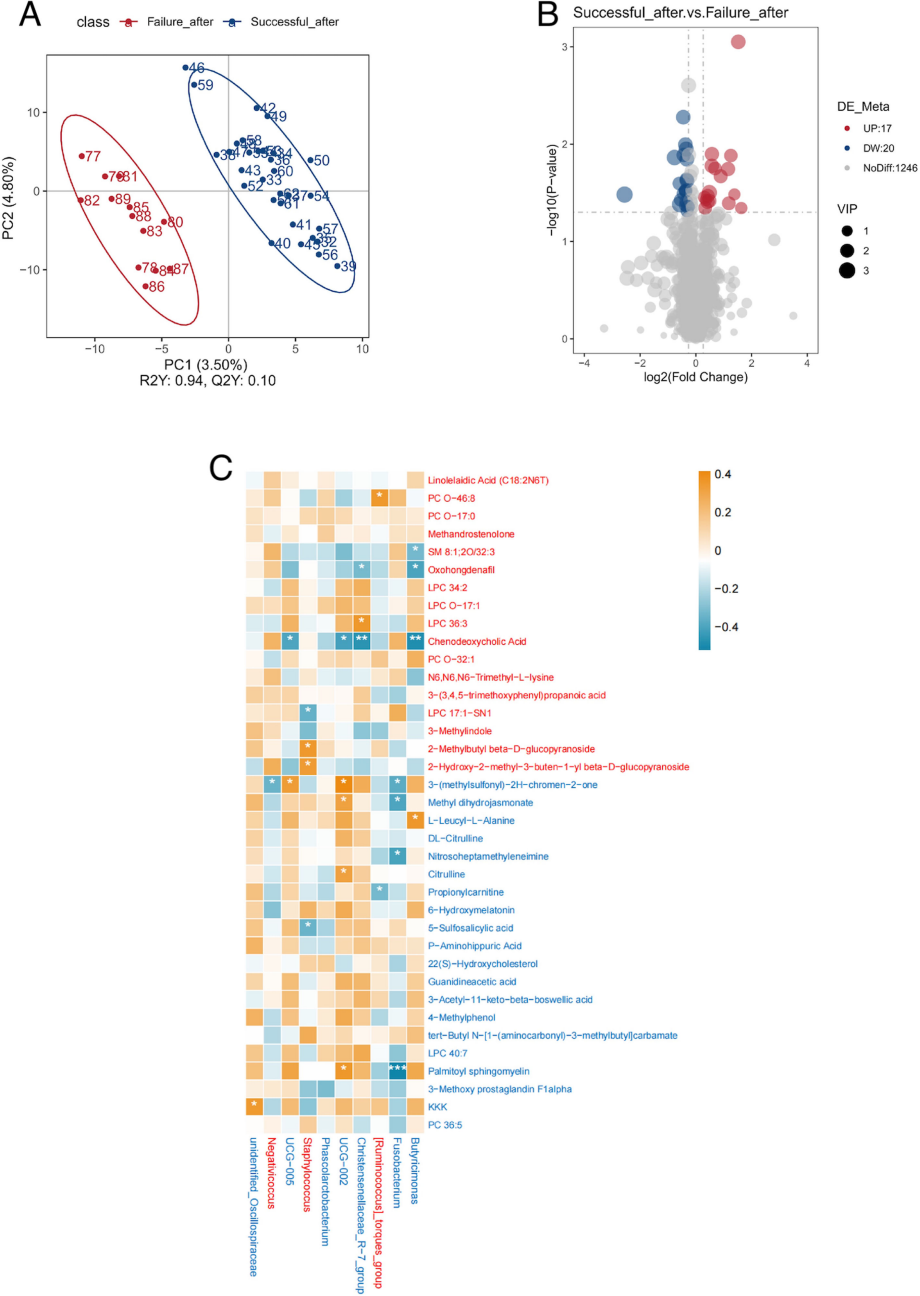
(A) PLS-DA for metabolites on the ninth day post-FET in the successful group and the failure group. The distance between each point represents the degree of difference in the metabolites of each sample. (B) Volcano plots were used to filter metabolites of interest based on log2(fold change) and −log10(*P* value) of metabolites. Up (UP) represents having a higher concentration in the successful group. Down (DW) represents having a higher concentration in the failure group. (C) Spearman correlation heatmap of different metabolites and bacterial genus selected by random forest in the failure group and the successful group in the ninth day post-FET. Red squares represent positive correlation; blue squares represent negative correlation. Red metabolite or bacterial genus represents more abundance in the successful group. Blue metabolite or bacterial genus represents more abundance evaluated in the failure group. *P < 0.05; **P < 0.01; ***P < 0.001.

PLS-DA was also used to investigate the differences in serum metabolites between the successful and failure groups on the ninth day after FET. After FET, the serum metabolite composition was again clearly distinguishable between the two groups. Additionally, a 200-cycle permutation test was performed to identify the best-fitted PLS-DA model. A total of 1,246 differential metabolites were identified between the two groups based on the PLS-DA model’s predictive value (VIP > 1.0) combined with *P* < 0.05. Differential analysis showed that 17 metabolites were significantly more abundant in the successful group, while 20 metabolites were significantly more abundant in the failure group (Fig. 13A and B).

Spearman correlation was then used to examine the relationships between these differential metabolites and the gut microbiota identified by the random forest model. We found that oxohongdenafil and chenodeoxycholic acid, which were increased in the successful group after FET, were correlated with more than two differential gut microbiota. Similarly, 3-(methylsulfonyl)-2H-chromen-2-one, methyl dihydrojasmonate, and palmitoyl sphingomyelin, which were increased in the failure group after FET, were also correlated with more than two differential gut microbiota (Fig. 13C).

## DISCUSSION

In recent years, the connection between IVF and microbiota—particularly the vaginal and endometrial microbiomes—has garnered increasing attention (42, 43). Numerous studies have identified vaginal microbiota as a critical factor in IVF success (44–46). However, as demonstrated in our previous research (20) and corroborated by other studies, pregnancy outcomes are not solely influenced by vaginal microbiota. Gut, oral, and other microbiomes also play essential roles in successful pregnancy (47–50). Despite this, very few studies have examined the variation in gut, oral, and vaginal microbiota throughout the FET process or how these changes influence FET outcomes. Given the substantial hormonal interventions and procedural aspects of FET, it is reasonable to hypothesize that these factors might influence microbial ecosystems at various body sites. Therefore, our study aims to provide preliminary insights into potential interactions between FET treatments and the human microbiome.

Our findings indicate that gut microbiota exhibited relatively minor changes during the FET process, with no significant shifts in alpha diversity or beta diversity. Additionally, the co-occurrence network topology of gut bacteria showed minimal declining trends after FET. While specific taxa, such as *Stenotrophomonas*, *Xanthomonadales*, and *Xanthomonadaceae*, were more abundant before FET, overall gut microbial composition showed limited variation. In contrast, oral microbiota displayed more pronounced variation. Diversity increased after FET, accompanied by a reduction in network complexity. Similarly, vaginal microbiota exhibited relatively large changes during the FET process, with a decrease in richness on the third day after FET and alterations in network characteristics on the ninth day after FET. These findings may reflect oral (10, 51) and vaginal microbial (52) shifts influenced by hormonal fluctuations. Hence, our study provides preliminary insights into microbial changes during the FET process, emphasizing the need for further research to better understand their potential implications.

To further explore biomarkers potentially associated with FET outcomes, we divided participants into successful and failure groups. Notably, the composition of gut microbiota differed significantly between the two groups at all time points. In the successful group, the abundance of *Anaerococcus* (involved in short-chain fatty acid production [53]), *Negativicoccus* (linked to short-chain fatty acid metabolic processes [54, 55]), and *Megasphaera micronuciformis* was higher, whereas the abundance of *Massiliprevotella massiliensis* was higher in the failure group. In our study, *Anaerococcus* (positively correlated with baseline AMH and negatively correlated with patient age) and *Negativicoccus* (negatively correlated with baseline FSH and positively correlated with D9 HCG) from the gut microbiota showed significant correlations with key reproductive hormones and metabolic markers, making them potential biomarkers for predicting FET success or failure. Despite these differences in composition, alpha diversity and beta diversity remained similar between the two groups. Our co-occurrence network analysis revealed that after FET, the gut microbiota network in the failure group became more complex, highlighting the need for further investigation into the potential relationship between gut microbiota complexity and implantation success. Few studies have explored the role of gut microbiota in IVF outcomes (25, 56). The former examined the relationship between fecal propionate and IVF success, while the latter investigated gut microbiota in IVF cycles with different follicle-to-oocyte indices. Our research provides a new perspective by considering gut microbiota in the broader context of reproductive success.

The differences in oral microbiota between the successful and failure groups were also noteworthy. The differences in the oral microbiota between the success group and the failure group are relatively small before FET, but they become increasingly significant after FET. Specifically, before FET, *Prevotella shahii* was significantly more abundant in FOA than in SOA. In SOB, *Alloprevotella* and *Prevotella* spp. were more abundant, whereas in FOB, *Verrucomicrobiota* and *Neisseria perflava* were more prevalent. In FOC, several taxa, including *Prevotella melaninogenica*, *Prevotella* subgroup 7, and *Veillonella*, were significantly enriched, while in SOC, *Comamonadaceae*, *Rothia*, and *Rothia aeria* were more abundant. Interestingly, the relationship between certain oral microorganisms (e.g., *Delftia* and *Allprevotella*) and lipid metabolism suggests a potential link between oral health and fertility outcomes. We also observed variations in oral microbiota richness and diversity across different groups during FET. The co-occurrence network of oral microbiota in the successful group showed higher complexity before FET, with a decrease in complexity after embryo transfer. These results suggest that a dynamic oral microbiota prior to FET may have potential benefits, but stabilization after transfer could be an important factor for success. Hormonal changes—particularly in estradiol and progesterone after pregnancy—are known to influence oral microbiota composition (57, 58). However, in our study, the differences between the two groups after FET did not align with the oral microbiota differences reported in the literature comparing early pregnancy with non-pregnancy (47, 51). This may suggest that FET exerts a unique hormonal influence on the oral microbiota.

Surprisingly, we found no significant differences in the vaginal microbiota between the two groups during FET. Unlike previous studies that concluded the vaginal microbiota affects ART outcomes (44, 45), our study participants exhibited greater homogeneity, as we specifically focused on FET patients and implemented strict inclusion criteria to ensure consistency. Furthermore, to minimize the influence of pathological factors, we excluded patients with vaginal inflammation or any vaginal symptoms. In our study, the vaginal microbiota in the successful group consistently displayed lower complexity, suggesting that a simpler vaginal microbiota network may contribute to improved implantation rates. Moreover, although fewer associations were found between vaginal microbiota and clinical characteristics, *Lactobacillus*’s correlation with prolactin suggested that a healthy vaginal environment might be important for hormonal balance during FET. Of course, future studies with larger sample sizes are needed to further explore the relationship between vaginal microbiota and implantation success rates. Additionally, further research is needed to explore the mechanisms linking the co-occurrence networks of oral and vaginal microbiota with FET outcomes.

To further investigate the relationship between gut microbiota and metabolic processes, we used random forest analysis to identify key gut microbiota genera that distinguish between the success and failure groups and LC-MS/MS to detect differential metabolites. This integrated approach enables us to explore potential associations between microbial features and metabolic profiles relevant to FET outcomes. Several noteworthy metabolites were found. Cystamine, present in higher concentrations in the successful group, has been shown to help reduce vascular stiffness (59) and holds potential therapeutic applications for managing vascular complications in diabetic retinopathy and other conditions associated with excessive vascular permeability (60). Cysteamine (61, 62), the active form of cystamine and a derivative of cysteine metabolism, is widely used to improve IVM (63), fertilization rates, and embryo development in ART (64, 65). It stimulates glutathione synthesis, which is essential for protecting oocytes from oxidative damage (66). In our study, cystamine showed a positive relationship with *Anaerococcus* (which was positively correlated with baseline AMH and negatively correlated with patient age) and *Negativicoccus* (which had a negative relationship with baseline FSH). Notably, KEGG pathway analysis, conducted via the KEGG website, revealed that *Anaerococcus* is enriched in the general metabolic pathways (map01100), particularly in sulfur-containing amino acid metabolism (e.g., cysteine and methionine pathways, map00270). This suggests that *Anaerococcus* may play a role in cysteamine production by modulating precursor availability through its metabolic activity. The observed positive correlation between *Anaerococcus* and cystamine levels could indicate a potential microbial-driven modulation of antioxidant defenses, which might help explain its association with better ovarian reserve markers (e.g., AMH) and younger age. However, further research, including validation experiments, is necessary to confirm the direct role of *Anaerococcus* in enhancing antioxidant defense mechanisms and its broader implications for reproductive health.

After the ninth day post-FET, we identified additional interesting metabolites. Chenodeoxycholic acid, found in higher concentrations in the successful group after FET, plays a key role in improving embryo implantation and enhancing metabolic health during early pregnancy. Previous research has shown that chenodeoxycholic acid achieves this by modulating the interaction between gut microbiota and host metabolites, reducing inflammation, oxidative stress, and insulin resistance (67). In our study, chenodeoxycholic acid was negatively correlated with UCG-002 (genus of *Oscillospiraceae)*, which is associated with TC and lipoprotein (a). Conversely, 3-(methylsulfonyl)-2H-chromen-2-one was found in higher concentrations in the failure group and is related to lipid metabolism (68), with a positive correlation with UCG-002. The correlation between FET outcomes and lipid metabolism was also observed with palmitoyl sphingomyelin. Previous studies have shown that palmitoyl sphingomyelin plays a role in sphingomyelin metabolism, which has been implicated in insulin resistance and other metabolic disorders (69). In our study, palmitoyl sphingomyelin had higher concentrations in the failure group and was positively correlated with UCG-002 after FET. However, whether these correlations arise from lipid metabolism influencing FET outcomes or whether FET outcomes lead to differences in lipid metabolism remains to be further investigated.

Our study has the following limitations. First, we did not include extraction and library preparation controls; however, we included a no-template (ddH₂O) control during PCR to monitor potential contamination. Second, we only explored the relationship between serum metabolites and gut microbiota, and future studies will include fecal metabolites to further validate their connection. Lastly, follow-up validation experiments for our preliminary findings are essential, and we can also investigate the role of microbiota after the ninth day post-FET and in other ART methods.

### Conclusion

During the FET process, while the gut microbiota experienced the least change, significant fluctuations were observed in the oral microbiota, with moderate changes in the vaginal microbiota. Furthermore, the gut microbiota exhibited the greatest differences between the success and failure groups, while the oral microbiota showed dynamic differences, with the variations gradually increasing as the FET process progressed. Vaginal microbiota may not play as significant a role in influencing FET outcomes as previously thought. Notably, two key genera, *Anaerococcus* and *Negativicoccus* from gut microbiota, were identified as potential microbial predictors. Additionally, we found that specific gut microbiota and metabolites exhibited significant differences between the success and failure groups before and 9 days after FET. Importantly, cystamine, which showed a positive correlation with *Anaerococcus* and *Negativicoccus* before FET, may potentially be beneficial for FET outcomes. After FET, lipid metabolism pathways were found to be associated with the FET outcomes.

## Data Availability

The original sequencing data presented in this study are publicly available in NCBI under accession number PRJNA1271528. Other data generated or analyzed during this study are included in this published article.

## SUPPLEMENTAL MATERIAL

File S1 mbio.00509-25-s0001.pdf Samples' information with primers and unique barcode.

File S2 mbio.00509-25-s0002.docx Statistical results obtained at each step of the data processing.

Supplemental material mbio.00509-25-s0003.pdf Files S3 to S10.

ASM does not own the copyrights to Supplemental Material that may be linked to, or accessed through, an article. The authors have granted ASM a non-exclusive, world-wide license to publish the Supplemental Material files. Please contact the corresponding author directly for reuse.
