# Correction for Zhang et al., “Will gut, oral, and vaginal microbiota influence the outcome of FET or be influenced by FET? A pilot study”

**DOI:** 10.1128/mbio.02313-25

**Published:** 2025-08-11

**Authors:** Yiwen Zhang, Zhao Zhang, Aihua Wu, Chengfang Xu, Zhe Li

**Affiliations:** 1 Department of Obstetrics, The Third Affiliated Hospital of Sun Yat-sen University, Guangzhou, Guangdong, China; 2 Department of Reproduction, Affiliated Dongguan People’s Hospital, Southern Medical University (Dongguan People’s Hospital), Dongguan, Guangdong, China

## AUTHOR CORRECTION

Volume 16, no. 7, e00509-25, 2025, https://doi.org/10.1128/mbio.00509-25. The article byline and affiliations should read as given in this correction.

