# Supplementary material for: Will gut, oral, and vaginal microbiota influence the outcome of FET or be influenced by FET? A pilot study
Source: mBio. 2025 Jun 17;16(7):e00509-25. doi: 10.1128/mbio.00509-25 (PMC12239586; doi:10.1128/mbio.00509-25)
Supplement: File S1 — Samples' information with primers and unique barcode. [file mbio.00509-25-s0001.pdf]

Sample	Barcode	Primer
GA10	AGGTACCA,TTCGCAAC	CCTAYGGGRBGCASCAG,GGACTACNNGGGTATCTAAT
GA11	GACTGCAA,CAAGTCTC	CCTAYGGGRBGCASCAG,GGACTACNNGGGTATCTAAT
GA12	CGCTTCAA,TCCAGCAT	CCTAYGGGRBGCASCAG,GGACTACNNGGGTATCTAAT
GA13	AGCGATAC,ATCAGGCA	CCTAYGGGRBGCASCAG,GGACTACNNGGGTATCTAAT
GA14	CCAGTAAG,CTAACAGG	CCTAYGGGRBGCASCAG,GGACTACNNGGGTATCTAAT
GA15	GATTCACC,GCCATCTA	CCTAYGGGRBGCASCAG,GGACTACNNGGGTATCTAAT
GA16	GTAACACG,GACCAAGT	CCTAYGGGRBGCASCAG,GGACTACNNGGGTATCTAAT
GA17	GCCTATAC,CCAGGAAT	CCTAYGGGRBGCASCAG,GGACTACNNGGGTATCTAAT
GA18	CCATAGCT,GCCATGAA	CCTAYGGGRBGCASCAG,GGACTACNNGGGTATCTAAT
GA19	AACGCTTC,CACTCAGT	CCTAYGGGRBGCASCAG,GGACTACNNGGGTATCTAAT
GA1	TAATCCGC,ATCAAGCG	CCTAYGGGRBGCASCAG,GGACTACNNGGGTATCTAAT
GA20	GCAAGCTA,CGACATAG	CCTAYGGGRBGCASCAG,GGACTACNNGGGTATCTAAT
GA21	GAGCAACT,AGACGTAC	CCTAYGGGRBGCASCAG,GGACTACNNGGGTATCTAAT
GA22	TACCAGAG,CCTATCAG	CCTAYGGGRBGCASCAG,GGACTACNNGGGTATCTAAT
GA23	CAGTGAAC,CAATTCGC	CCTAYGGGRBGCASCAG,GGACTACNNGGGTATCTAAT
GA24	AGCCGATA,GGTCACAA	CCTAYGGGRBGCASCAG,GGACTACNNGGGTATCTAAT
GA25	CCATATCG,CTAGAGAC	CCTAYGGGRBGCASCAG,GGACTACNNGGGTATCTAAT
GA26	AGGCTACA,TACCACTG	CCTAYGGGRBGCASCAG,GGACTACNNGGGTATCTAAT
GA27	AGGACCTA,CGTATCAC	CCTAYGGGRBGCASCAG,GGACTACNNGGGTATCTAAT
GA28	CAATGCAG,GAACAGCT	CCTAYGGGRBGCASCAG,GGACTACNNGGGTATCTAAT
GA29	CTCTAAGC,CATCTAGC	CCTAYGGGRBGCASCAG,GGACTACNNGGGTATCTAAT
GA2	TAATCCGC,ATCAAGCG	CCTAYGGGRBGCASCAG,GGACTACNNGGGTATCTAAT
GA30	CATGACGA,TGAACAGC	CCTAYGGGRBGCASCAG,GGACTACNNGGGTATCTAAT
GA31	ATCGTACC,TACCTACG	CCTAYGGGRBGCASCAG,GGACTACNNGGGTATCTAAT
GA32	AACAGGTC,CGTTCACA	CCTAYGGGRBGCASCAG,GGACTACNNGGGTATCTAAT
GA33	TACAGTCC,CACGTTCA	CCTAYGGGRBGCASCAG,GGACTACNNGGGTATCTAAT
GA34	ACGTCCAT,AGAAGTCC	CCTAYGGGRBGCASCAG,GGACTACNNGGGTATCTAAT
GA35	ATAACCGG,GCCAATGA	CCTAYGGGRBGCASCAG,GGACTACNNGGGTATCTAAT
GA36	CCTGACAT,CCAGTCTA	CCTAYGGGRBGCASCAG,GGACTACNNGGGTATCTAAT

GA37	CAGCATAG,CATGACCT	CCTAYGGGRBGCASCAG,GGACTACNNGGGTATCTAAT
GA38	GCTCACAT,ACCGATCT	CCTAYGGGRBGCASCAG,GGACTACNNGGGTATCTAAT
GA39	CTACACGT,CTCATGAC	CCTAYGGGRBGCASCAG,GGACTACNNGGGTATCTAAT
GA3	TACGCATC,AGACCTTC	CCTAYGGGRBGCASCAG,GGACTACNNGGGTATCTAAT
GA40	CTCACTGA,CGGACAAT	CCTAYGGGRBGCASCAG,GGACTACNNGGGTATCTAAT
GA41	ATAGGACC,GAAGATCC	CCTAYGGGRBGCASCAG,GGACTACNNGGGTATCTAAT
GA42	CAGCTACT,CTCAGTAC	CCTAYGGGRBGCASCAG,GGACTACNNGGGTATCTAAT
GA43	CCAATGTC,TCCACTAG	CCTAYGGGRBGCASCAG,GGACTACNNGGGTATCTAAT
GA44	ACGCAGAT,ATCGGAAC	CCTAYGGGRBGCASCAG,GGACTACNNGGGTATCTAAT
GA45	TAACTCGC,ATCATCCG	CCTAYGGGRBGCASCAG,GGACTACNNGGGTATCTAAT
GA46	CACAGCTT,ATCCAGGA	CCTAYGGGRBGCASCAG,GGACTACNNGGGTATCTAAT
GA47	CAGCAATG,GTGCAACA	CCTAYGGGRBGCASCAG,GGACTACNNGGGTATCTAAT
GA48	CCTTACGA,ATACTGCC	CCTAYGGGRBGCASCAG,GGACTACNNGGGTATCTAAT
GA49	CGGTAACA,ACCAGAGT	CCTAYGGGRBGCASCAG,GGACTACNNGGGTATCTAAT
GA4	ATTGCACC,TTGACACC	CCTAYGGGRBGCASCAG,GGACTACNNGGGTATCTAAT
GA50	ACGGATAC,GTCCACTA	CCTAYGGGRBGCASCAG,GGACTACNNGGGTATCTAAT
GA51	AAGTGACC,CCTTCAAG	CCTAYGGGRBGCASCAG,GGACTACNNGGGTATCTAAT
GA52	GCAGTAAC,ATTCACCG	CCTAYGGGRBGCASCAG,GGACTACNNGGGTATCTAAT
GA53	AGCGAATC,ACGTGAAC	CCTAYGGGRBGCASCAG,GGACTACNNGGGTATCTAAT
GA54	GCCGTAAT,CCTGAGAA	CCTAYGGGRBGCASCAG,GGACTACNNGGGTATCTAAT
GA55	AGTCCGAA,TATACCGC	CCTAYGGGRBGCASCAG,GGACTACNNGGGTATCTAAT
GA56	AGACTCAG,ACAGAGTC	CCTAYGGGRBGCASCAG,GGACTACNNGGGTATCTAAT
GA57	GTCACAAG,CCTAGGAA	CCTAYGGGRBGCASCAG,GGACTACNNGGGTATCTAAT
GA58	ACAATGGC,ATGAACGC	CCTAYGGGRBGCASCAG,GGACTACNNGGGTATCTAAT
GA59	CACTGAAG,AGCACTCT	CCTAYGGGRBGCASCAG,GGACTACNNGGGTATCTAAT
GA5	AGCATCTC,GTGAACCA	CCTAYGGGRBGCASCAG,GGACTACNNGGGTATCTAAT
GA6	CAACTGAG,ACATGTCC	CCTAYGGGRBGCASCAG,GGACTACNNGGGTATCTAAT
GA7	GAATGACC,CATCCGAT	CCTAYGGGRBGCASCAG,GGACTACNNGGGTATCTAAT
GA8	CATTCGCA,CCTCATAG	CCTAYGGGRBGCASCAG,GGACTACNNGGGTATCTAAT
GA9	CCGATTCA,ACTCGACT	CCTAYGGGRBGCASCAG,GGACTACNNGGGTATCTAAT

GB10	CCTAGCTA,CGATAACG	CCTAYGGGRBGCASCAG,GGACTACNNGGGTATCTAAT
GB11	ACGGACTA,GCTCAAGA	CCTAYGGGRBGCASCAG,GGACTACNNGGGTATCTAAT
GB12	AAGCTTCC,CCACTAGT	CCTAYGGGRBGCASCAG,GGACTACNNGGGTATCTAAT
GB13	AACGCTTC,CACTCAGT	CCTAYGGGRBGCASCAG,GGACTACNNGGGTATCTAAT
GB14	TACCAGAG,CCTATCAG	CCTAYGGGRBGCASCAG,GGACTACNNGGGTATCTAAT
GB15	ATAACCGG,GCCAATGA	CCTAYGGGRBGCASCAG,GGACTACNNGGGTATCTAAT
GB16	ACAATGGC,ATGAACGC	CCTAYGGGRBGCASCAG,GGACTACNNGGGTATCTAAT
GB17	CAATGGCA,GCCTAACT	CCTAYGGGRBGCASCAG,GGACTACNNGGGTATCTAAT
GB18	AGATCCTC,GAACTTCC	CCTAYGGGRBGCASCAG,GGACTACNNGGGTATCTAAT
GB19	AAGCGTCA,ATACGGCA	CCTAYGGGRBGCASCAG,GGACTACNNGGGTATCTAAT
GB1	AGCCGATA,GGTCACAA	CCTAYGGGRBGCASCAG,GGACTACNNGGGTATCTAAT
GB20	AGCCACTT,CTAGCGAA	CCTAYGGGRBGCASCAG,GGACTACNNGGGTATCTAAT
GB21	TCGCCTAA,CACTAAGG	CCTAYGGGRBGCASCAG,GGACTACNNGGGTATCTAAT
GB22	GGCACTAA,GCTTACAC	CCTAYGGGRBGCASCAG,GGACTACNNGGGTATCTAAT
GB23	GTAACAGC,ACGTACCT	CCTAYGGGRBGCASCAG,GGACTACNNGGGTATCTAAT
GB24	TAAGCAGC,ACGCCTAT	CCTAYGGGRBGCASCAG,GGACTACNNGGGTATCTAAT
GB25	CCGAGTAA,TCAACGGA	CCTAYGGGRBGCASCAG,GGACTACNNGGGTATCTAAT
GB26	GACAAGCT,ATGAGACC	CCTAYGGGRBGCASCAG,GGACTACNNGGGTATCTAAT
GB27	CTGTAACC,TAGGCAAC	CCTAYGGGRBGCASCAG,GGACTACNNGGGTATCTAAT
GB28	TAATCCGC,ATCAAGCG	CCTAYGGGRBGCASCAG,GGACTACNNGGGTATCTAAT
GB29	TACGCATC,AGACCTTC	CCTAYGGGRBGCASCAG,GGACTACNNGGGTATCTAAT
GB2	CCATATCG,CTAGAGAC	CCTAYGGGRBGCASCAG,GGACTACNNGGGTATCTAAT
GB30	ATTGCACC,TTGACACC	CCTAYGGGRBGCASCAG,GGACTACNNGGGTATCTAAT
GB31	AGCATCTC,GTGAACCA	CCTAYGGGRBGCASCAG,GGACTACNNGGGTATCTAAT
GB32	CAACTGAG,ACATGTCC	CCTAYGGGRBGCASCAG,GGACTACNNGGGTATCTAAT
GB33	GAATGACC,CATCCGAT	CCTAYGGGRBGCASCAG,GGACTACNNGGGTATCTAAT
GB34	CATTCGCA,CCTCATAG	CCTAYGGGRBGCASCAG,GGACTACNNGGGTATCTAAT
GB35	AGGTACCA,TTCGCAAC	CCTAYGGGRBGCASCAG,GGACTACNNGGGTATCTAAT
GB36	GACTGCAA,CAAGTCTC	CCTAYGGGRBGCASCAG,GGACTACNNGGGTATCTAAT
GB37	CGCTTCAA,TCCAGCAT	CCTAYGGGRBGCASCAG,GGACTACNNGGGTATCTAAT

GB38	AGCGATAC,ATCAGGCA	CCTAYGGGRBGCASCAG,GGACTACNNGGGTATCTAAT
GB39	CCAGTAAG,CTAACAGG	CCTAYGGGRBGCASCAG,GGACTACNNGGGTATCTAAT
GB3	AGGCTACA,TACCACTG	CCTAYGGGRBGCASCAG,GGACTACNNGGGTATCTAAT
GB40	GATTCACC,GCCATCTA	CCTAYGGGRBGCASCAG,GGACTACNNGGGTATCTAAT
GB41	GTAACACG,GACCAAGT	CCTAYGGGRBGCASCAG,GGACTACNNGGGTATCTAAT
GB42	GCCTATAC,CCAGGAAT	CCTAYGGGRBGCASCAG,GGACTACNNGGGTATCTAAT
GB43	CCATAGCT,GCCATGAA	CCTAYGGGRBGCASCAG,GGACTACNNGGGTATCTAAT
GB44	CCATGCAT,CTCGCAAT	CCTAYGGGRBGCASCAG,GGACTACNNGGGTATCTAAT
GB45	GCAAGCTA,CGACATAG	CCTAYGGGRBGCASCAG,GGACTACNNGGGTATCTAAT
GB46	GAGCAACT,AGACGTAC	CCTAYGGGRBGCASCAG,GGACTACNNGGGTATCTAAT
GB47	TATACGCC,CCGAATAG	CCTAYGGGRBGCASCAG,GGACTACNNGGGTATCTAAT
GB48	CAGTGAAC,CAATTCGC	CCTAYGGGRBGCASCAG,GGACTACNNGGGTATCTAAT
GB49	AGCCGATA,GGTCACAA	CCTAYGGGRBGCASCAG,GGACTACNNGGGTATCTAAT
GB4	AGGACCTA,CGTATCAC	CCTAYGGGRBGCASCAG,GGACTACNNGGGTATCTAAT
GB50	CCATATCG,CTAGAGAC	CCTAYGGGRBGCASCAG,GGACTACNNGGGTATCTAAT
GB51	AGGCTACA,TACCACTG	CCTAYGGGRBGCASCAG,GGACTACNNGGGTATCTAAT
GB52	AGGACCTA,CGTATCAC	CCTAYGGGRBGCASCAG,GGACTACNNGGGTATCTAAT
GB53	CAATGCAG,GAACAGCT	CCTAYGGGRBGCASCAG,GGACTACNNGGGTATCTAAT
GB54	CTCTAAGC,CATCTAGC	CCTAYGGGRBGCASCAG,GGACTACNNGGGTATCTAAT
GB55	CATGACGA,TGAACAGC	CCTAYGGGRBGCASCAG,GGACTACNNGGGTATCTAAT
GB56	ATCGTACC,TACCTACG	CCTAYGGGRBGCASCAG,GGACTACNNGGGTATCTAAT
GB57	AACAGGTC,CGTTCACA	CCTAYGGGRBGCASCAG,GGACTACNNGGGTATCTAAT
GB58	TACAGTCC,CACGTTCA	CCTAYGGGRBGCASCAG,GGACTACNNGGGTATCTAAT
GB59	ACGTCCAT,AGAAGTCC	CCTAYGGGRBGCASCAG,GGACTACNNGGGTATCTAAT
GB5	CAATGCAG,GAACAGCT	CCTAYGGGRBGCASCAG,GGACTACNNGGGTATCTAAT
GB6	GCCTAAGA,GTACCAGA	CCTAYGGGRBGCASCAG,GGACTACNNGGGTATCTAAT
GB7	CCGAGTAA,TCAACGGA	CCTAYGGGRBGCASCAG,GGACTACNNGGGTATCTAAT
GB8	CTTCCAGA,CTTCGAAC	CCTAYGGGRBGCASCAG,GGACTACNNGGGTATCTAAT
GB9	CGATCAAG,AACATGGC	CCTAYGGGRBGCASCAG,GGACTACNNGGGTATCTAAT
GC10	CAATGCAG,GAACAGCT	CCTAYGGGRBGCASCAG,GGACTACNNGGGTATCTAAT

GC11	CTCTAAGC,CATCTAGC	CCTAYGGGRBGCASCAG,GGACTACNNGGGTATCTAAT
GC12	CATGACGA,TGAACAGC	CCTAYGGGRBGCASCAG,GGACTACNNGGGTATCTAAT
GC13	ATCGTACC,TACCTACG	CCTAYGGGRBGCASCAG,GGACTACNNGGGTATCTAAT
GC14	AACAGGTC,CGTTCACA	CCTAYGGGRBGCASCAG,GGACTACNNGGGTATCTAAT
GC15	TACAGTCC,CACGTTCA	CCTAYGGGRBGCASCAG,GGACTACNNGGGTATCTAAT
GC16	ACGTCCAT,AGAAGTCC	CCTAYGGGRBGCASCAG,GGACTACNNGGGTATCTAAT
GC17	ACTTAGCC,GAGCTACA	CCTAYGGGRBGCASCAG,GGACTACNNGGGTATCTAAT
GC18	CCTGACAT,CCAGTCTA	CCTAYGGGRBGCASCAG,GGACTACNNGGGTATCTAAT
GC19	CAGCATAG,CATGACCT	CCTAYGGGRBGCASCAG,GGACTACNNGGGTATCTAAT
GC1	CCATGCAT,CTCGCAAT	CCTAYGGGRBGCASCAG,GGACTACNNGGGTATCTAAT
GC20	GCCTAAGA,GTACCAGA	CCTAYGGGRBGCASCAG,GGACTACNNGGGTATCTAAT
GC21	CTTCCAGA,CTTCGAAC	CCTAYGGGRBGCASCAG,GGACTACNNGGGTATCTAAT
GC22	CGATCAAG,AACATGGC	CCTAYGGGRBGCASCAG,GGACTACNNGGGTATCTAAT
GC23	CCTAGCTA,CGATAACG	CCTAYGGGRBGCASCAG,GGACTACNNGGGTATCTAAT
GC24	TGACACCT,GGACTACA	CCTAYGGGRBGCASCAG,GGACTACNNGGGTATCTAAT
GC25	AAGCTTCC,CCACTAGT	CCTAYGGGRBGCASCAG,GGACTACNNGGGTATCTAAT
GC26	AACGCTTC,CACTCAGT	CCTAYGGGRBGCASCAG,GGACTACNNGGGTATCTAAT
GC27	TACCAGAG,CCTATCAG	CCTAYGGGRBGCASCAG,GGACTACNNGGGTATCTAAT
GC28	ATAACCGG,GCCAATGA	CCTAYGGGRBGCASCAG,GGACTACNNGGGTATCTAAT
GC29	ACAATGGC,ATGAACGC	CCTAYGGGRBGCASCAG,GGACTACNNGGGTATCTAAT
GC2	GCAAGCTA,CGACATAG	CCTAYGGGRBGCASCAG,GGACTACNNGGGTATCTAAT
GC30	CAATGGCA,GCCTAACT	CCTAYGGGRBGCASCAG,GGACTACNNGGGTATCTAAT
GC31	AGATCCTC,GAACTTCC	CCTAYGGGRBGCASCAG,GGACTACNNGGGTATCTAAT
GC32	AAGCGTCA,ATACGGCA	CCTAYGGGRBGCASCAG,GGACTACNNGGGTATCTAAT
GC33	AGCCACTT,CTAGCGAA	CCTAYGGGRBGCASCAG,GGACTACNNGGGTATCTAAT
GC34	TCGCCTAA,CACTAAGG	CCTAYGGGRBGCASCAG,GGACTACNNGGGTATCTAAT
GC35	GGCACTAA,GCTTACAC	CCTAYGGGRBGCASCAG,GGACTACNNGGGTATCTAAT
GC36	AGACTCCT,CTCTAGAC	CCTAYGGGRBGCASCAG,GGACTACNNGGGTATCTAAT
GC37	TAAGCAGC,ACGCCTAT	CCTAYGGGRBGCASCAG,GGACTACNNGGGTATCTAAT
GC38	CCGAGTAA,TCAACGGA	CCTAYGGGRBGCASCAG,GGACTACNNGGGTATCTAAT

GC39	GACAAGCT,ATGAGACC	CCTAYGGGRBGCASCAG,GGACTACNNGGGTATCTAAT
GC3	GAGCAACT,AGACGTAC	CCTAYGGGRBGCASCAG,GGACTACNNGGGTATCTAAT
GC40	CTGTAACC,TAGGCAAC	CCTAYGGGRBGCASCAG,GGACTACNNGGGTATCTAAT
GC41	TAATCCGC,ATCAAGCG	CCTAYGGGRBGCASCAG,GGACTACNNGGGTATCTAAT
GC42	TACGCATC,AGACCTTC	CCTAYGGGRBGCASCAG,GGACTACNNGGGTATCTAAT
GC43	CACGCTAT,TGCGACAA	CCTAYGGGRBGCASCAG,GGACTACNNGGGTATCTAAT
GC44	AGCATCTC,GTGAACCA	CCTAYGGGRBGCASCAG,GGACTACNNGGGTATCTAAT
GC45	CAACTGAG,ACATGTCC	CCTAYGGGRBGCASCAG,GGACTACNNGGGTATCTAAT
GC46	GAATGACC,CATCCGAT	CCTAYGGGRBGCASCAG,GGACTACNNGGGTATCTAAT
GC47	CATTCGCA,CCTCATAG	CCTAYGGGRBGCASCAG,GGACTACNNGGGTATCTAAT
GC48	CGATCCTA,GAATGCAC	CCTAYGGGRBGCASCAG,GGACTACNNGGGTATCTAAT
GC49	GACTGCAA,CAAGTCTC	CCTAYGGGRBGCASCAG,GGACTACNNGGGTATCTAAT
GC4	TATACGCC,CCGAATAG	CCTAYGGGRBGCASCAG,GGACTACNNGGGTATCTAAT
GC50	CGCTTCAA,TCCAGCAT	CCTAYGGGRBGCASCAG,GGACTACNNGGGTATCTAAT
GC51	AGCGATAC,ATCAGGCA	CCTAYGGGRBGCASCAG,GGACTACNNGGGTATCTAAT
GC52	CCAGTAAG,CTAACAGG	CCTAYGGGRBGCASCAG,GGACTACNNGGGTATCTAAT
GC53	GATTCACC,GCCATCTA	CCTAYGGGRBGCASCAG,GGACTACNNGGGTATCTAAT
GC54	GTAACACG,GACCAAGT	CCTAYGGGRBGCASCAG,GGACTACNNGGGTATCTAAT
GC55	AGCCACTT,CTAGCGAA	CCTAYGGGRBGCASCAG,GGACTACNNGGGTATCTAAT
GC56	GCCTATAC,CCAGGAAT	CCTAYGGGRBGCASCAG,GGACTACNNGGGTATCTAAT
GC57	CCATAGCT,GCCATGAA	CCTAYGGGRBGCASCAG,GGACTACNNGGGTATCTAAT
GC58	CCATGCAT,CTCGCAAT	CCTAYGGGRBGCASCAG,GGACTACNNGGGTATCTAAT
GC59	GACCTTAC,ACCGATGA	CCTAYGGGRBGCASCAG,GGACTACNNGGGTATCTAAT
GC5	CAGTGAAC,CAATTCGC	CCTAYGGGRBGCASCAG,GGACTACNNGGGTATCTAAT
GC6	AGCCGATA,GGTCACAA	CCTAYGGGRBGCASCAG,GGACTACNNGGGTATCTAAT
GC7	CCATATCG,CTAGAGAC	CCTAYGGGRBGCASCAG,GGACTACNNGGGTATCTAAT
GC8	AGGCTACA,TACCACTG	CCTAYGGGRBGCASCAG,GGACTACNNGGGTATCTAAT
GC9	AGGACCTA,CGTATCAC	CCTAYGGGRBGCASCAG,GGACTACNNGGGTATCTAAT
OA10	TACCAGAG,CCTATCAG	CCTAYGGGRBGCASCAG,GGACTACNNGGGTATCTAAT
OA11	ATAACCGG,GCCAATGA	CCTAYGGGRBGCASCAG,GGACTACNNGGGTATCTAAT

OA12	ACAATGGC,ATGAACGC	CCTAYGGGRBGCASCAG,GGACTACNNGGGTATCTAAT
OA13	CAATGGCA,GCCTAACT	CCTAYGGGRBGCASCAG,GGACTACNNGGGTATCTAAT
OA14	AGATCCTC,GAACTTCC	CCTAYGGGRBGCASCAG,GGACTACNNGGGTATCTAAT
OA15	AAGCGTCA,ATACGGCA	CCTAYGGGRBGCASCAG,GGACTACNNGGGTATCTAAT
OA16	AGCCACTT,CTAGCGAA	CCTAYGGGRBGCASCAG,GGACTACNNGGGTATCTAAT
OA17	TCGCCTAA,CACTAAGG	CCTAYGGGRBGCASCAG,GGACTACNNGGGTATCTAAT
OA18	GGCACTAA,GCTTACAC	CCTAYGGGRBGCASCAG,GGACTACNNGGGTATCTAAT
OA19	GTAACAGC,ACGTACCT	CCTAYGGGRBGCASCAG,GGACTACNNGGGTATCTAAT
OA1	GACAAGCT,ATGAGACC	CCTAYGGGRBGCASCAG,GGACTACNNGGGTATCTAAT
OA20	TAAGCAGC,ACGCCTAT	CCTAYGGGRBGCASCAG,GGACTACNNGGGTATCTAAT
OA21	CCGAGTAA,TCAACGGA	CCTAYGGGRBGCASCAG,GGACTACNNGGGTATCTAAT
OA22	GACAAGCT,ATGAGACC	CCTAYGGGRBGCASCAG,GGACTACNNGGGTATCTAAT
OA23	CTGTAACC,TAGGCAAC	CCTAYGGGRBGCASCAG,GGACTACNNGGGTATCTAAT
OA24	TAATCCGC,ATCAAGCG	CCTAYGGGRBGCASCAG,GGACTACNNGGGTATCTAAT
OA25	TACGCATC,AGACCTTC	CCTAYGGGRBGCASCAG,GGACTACNNGGGTATCTAAT
OA26	ATTGCACC,TTGACACC	CCTAYGGGRBGCASCAG,GGACTACNNGGGTATCTAAT
OA27	TAATCCGC,ATCAAGCG	CCTAYGGGRBGCASCAG,GGACTACNNGGGTATCTAAT
OA28	CAACTGAG,ACATGTCC	CCTAYGGGRBGCASCAG,GGACTACNNGGGTATCTAAT
OA29	TACGCATC,AGACCTTC	CCTAYGGGRBGCASCAG,GGACTACNNGGGTATCTAAT
OA2	GCCTAAGA,GTACCAGA	CCTAYGGGRBGCASCAG,GGACTACNNGGGTATCTAAT
OA30	CATTCGCA,CCTCATAG	CCTAYGGGRBGCASCAG,GGACTACNNGGGTATCTAAT
OA31	AGGTACCA,TTCGCAAC	CCTAYGGGRBGCASCAG,GGACTACNNGGGTATCTAAT
OA32	GACTGCAA,CAAGTCTC	CCTAYGGGRBGCASCAG,GGACTACNNGGGTATCTAAT
OA33	CGCTTCAA,TCCAGCAT	CCTAYGGGRBGCASCAG,GGACTACNNGGGTATCTAAT
OA34	AGCGATAC,ATCAGGCA	CCTAYGGGRBGCASCAG,GGACTACNNGGGTATCTAAT
OA35	CCAGTAAG,CTAACAGG	CCTAYGGGRBGCASCAG,GGACTACNNGGGTATCTAAT
OA36	GATTCACC,GCCATCTA	CCTAYGGGRBGCASCAG,GGACTACNNGGGTATCTAAT
OA37	GTAACACG,GACCAAGT	CCTAYGGGRBGCASCAG,GGACTACNNGGGTATCTAAT
OA38	GCCTATAC,CCAGGAAT	CCTAYGGGRBGCASCAG,GGACTACNNGGGTATCTAAT
OA39	CCATAGCT,GCCATGAA	CCTAYGGGRBGCASCAG,GGACTACNNGGGTATCTAAT

OA3	CTTCCAGA,CTTCGAAC	CCTAYGGGRBGCASCAG,GGACTACNNGGGTATCTAAT
OA40	CCATGCAT,CTCGCAAT	CCTAYGGGRBGCASCAG,GGACTACNNGGGTATCTAAT
OA41	GCAAGCTA,CGACATAG	CCTAYGGGRBGCASCAG,GGACTACNNGGGTATCTAAT
OA42	GAGCAACT,AGACGTAC	CCTAYGGGRBGCASCAG,GGACTACNNGGGTATCTAAT
OA43	ATTGCACC,TTGACACC	CCTAYGGGRBGCASCAG,GGACTACNNGGGTATCTAAT
OA44	TCAAGCGA,ACGAGTAC	CCTAYGGGRBGCASCAG,GGACTACNNGGGTATCTAAT
OA45	AGCCGATA,GGTCACAA	CCTAYGGGRBGCASCAG,GGACTACNNGGGTATCTAAT
OA46	CCATATCG,CTAGAGAC	CCTAYGGGRBGCASCAG,GGACTACNNGGGTATCTAAT
OA47	AGGCTACA,TACCACTG	CCTAYGGGRBGCASCAG,GGACTACNNGGGTATCTAAT
OA48	AGGACCTA,CGTATCAC	CCTAYGGGRBGCASCAG,GGACTACNNGGGTATCTAAT
OA49	CAATGCAG,GAACAGCT	CCTAYGGGRBGCASCAG,GGACTACNNGGGTATCTAAT
OA4	CGATCAAG,AACATGGC	CCTAYGGGRBGCASCAG,GGACTACNNGGGTATCTAAT
OA50	CTCTAAGC,CATCTAGC	CCTAYGGGRBGCASCAG,GGACTACNNGGGTATCTAAT
OA51	CATGACGA,TGAACAGC	CCTAYGGGRBGCASCAG,GGACTACNNGGGTATCTAAT
OA52	AGCATCTC,GTGAACCA	CCTAYGGGRBGCASCAG,GGACTACNNGGGTATCTAAT
OA53	CAACTGAG,ACATGTCC	CCTAYGGGRBGCASCAG,GGACTACNNGGGTATCTAAT
OA54	GAATGACC,CATCCGAT	CCTAYGGGRBGCASCAG,GGACTACNNGGGTATCTAAT
OA55	CATTCGCA,CCTCATAG	CCTAYGGGRBGCASCAG,GGACTACNNGGGTATCTAAT
OA56	AGGTACCA,TTCGCAAC	CCTAYGGGRBGCASCAG,GGACTACNNGGGTATCTAAT
OA57	CCTGACAT,CCAGTCTA	CCTAYGGGRBGCASCAG,GGACTACNNGGGTATCTAAT
OA58	CAGCATAG,CATGACCT	CCTAYGGGRBGCASCAG,GGACTACNNGGGTATCTAAT
OA59	GCTCACAT,ACCGATCT	CCTAYGGGRBGCASCAG,GGACTACNNGGGTATCTAAT
OA5	CCTAGCTA,CGATAACG	CCTAYGGGRBGCASCAG,GGACTACNNGGGTATCTAAT
OA6	ACGGACTA,GCTCAAGA	CCTAYGGGRBGCASCAG,GGACTACNNGGGTATCTAAT
OA7	AAGCTTCC,CCACTAGT	CCTAYGGGRBGCASCAG,GGACTACNNGGGTATCTAAT
OA8	AACGCTTC,CACTCAGT	CCTAYGGGRBGCASCAG,GGACTACNNGGGTATCTAAT
OA9	TACGCATC,AGACCTTC	CCTAYGGGRBGCASCAG,GGACTACNNGGGTATCTAAT
OB10	TCCACTGA,ATCGCAAG	CCTAYGGGRBGCASCAG,GGACTACNNGGGTATCTAAT
OB11	TTCCGAAC,GGACAATC	CCTAYGGGRBGCASCAG,GGACTACNNGGGTATCTAAT
OB12	CATGATCC,GACACTTC	CCTAYGGGRBGCASCAG,GGACTACNNGGGTATCTAAT

OB13	CTAGAACG,GATACTCC	CCTAYGGGRBGCASCAG,GGACTACNNGGGTATCTAAT
OB14	AACTCCGT,TCAGGCAA	CCTAYGGGRBGCASCAG,GGACTACNNGGGTATCTAAT
OB15	GCAGATAC,ACACTGTC	CCTAYGGGRBGCASCAG,GGACTACNNGGGTATCTAAT
OB16	CAATGGCA,GCCTAACT	CCTAYGGGRBGCASCAG,GGACTACNNGGGTATCTAAT
OB17	GCCTAAGA,GTACCAGA	CCTAYGGGRBGCASCAG,GGACTACNNGGGTATCTAAT
OB18	CTTCCAGA,CTTCGAAC	CCTAYGGGRBGCASCAG,GGACTACNNGGGTATCTAAT
OB19	CGATCAAG,AACATGGC	CCTAYGGGRBGCASCAG,GGACTACNNGGGTATCTAAT
OB1	CGGTACAA,CATGCAAG	CCTAYGGGRBGCASCAG,GGACTACNNGGGTATCTAAT
OB20	CCTAGCTA,CGATAACG	CCTAYGGGRBGCASCAG,GGACTACNNGGGTATCTAAT
OB21	ACGGACTA,GCTCAAGA	CCTAYGGGRBGCASCAG,GGACTACNNGGGTATCTAAT
OB22	AAGCTTCC,CCACTAGT	CCTAYGGGRBGCASCAG,GGACTACNNGGGTATCTAAT
OB23	AGCCACTT,CTAGCGAA	CCTAYGGGRBGCASCAG,GGACTACNNGGGTATCTAAT
OB24	TACCAGAG,CCTATCAG	CCTAYGGGRBGCASCAG,GGACTACNNGGGTATCTAAT
OB25	CCAGTAAG,CTAACAGG	CCTAYGGGRBGCASCAG,GGACTACNNGGGTATCTAAT
OB26	ACAATGGC,ATGAACGC	CCTAYGGGRBGCASCAG,GGACTACNNGGGTATCTAAT
OB27	GGCACTAA,GCTTACAC	CCTAYGGGRBGCASCAG,GGACTACNNGGGTATCTAAT
OB28	GATTCACC,GCCATCTA	CCTAYGGGRBGCASCAG,GGACTACNNGGGTATCTAAT
OB2	CCAGATTC,CTTCACAG	CCTAYGGGRBGCASCAG,GGACTACNNGGGTATCTAAT
OB30	AGCCACTT,CTAGCGAA	CCTAYGGGRBGCASCAG,GGACTACNNGGGTATCTAAT
OB31	TCGCCTAA,CACTAAGG	CCTAYGGGRBGCASCAG,GGACTACNNGGGTATCTAAT
OB32	GGCACTAA,GCTTACAC	CCTAYGGGRBGCASCAG,GGACTACNNGGGTATCTAAT
OB33	GTAACAGC,ACGTACCT	CCTAYGGGRBGCASCAG,GGACTACNNGGGTATCTAAT
OB34	CCGAGTAA,TCAACGGA	CCTAYGGGRBGCASCAG,GGACTACNNGGGTATCTAAT
OB35	CCGAGTAA,TCAACGGA	CCTAYGGGRBGCASCAG,GGACTACNNGGGTATCTAAT
OB36	GACAAGCT,ATGAGACC	CCTAYGGGRBGCASCAG,GGACTACNNGGGTATCTAAT
OB37	CTGTAACC,TAGGCAAC	CCTAYGGGRBGCASCAG,GGACTACNNGGGTATCTAAT
OB38	GACAAGCT,ATGAGACC	CCTAYGGGRBGCASCAG,GGACTACNNGGGTATCTAAT
OB39	TACGCATC,AGACCTTC	CCTAYGGGRBGCASCAG,GGACTACNNGGGTATCTAAT
OB3	GGCATCAA,TTACACCG	CCTAYGGGRBGCASCAG,GGACTACNNGGGTATCTAAT
OB40	ATTGCACC,TTGACACC	CCTAYGGGRBGCASCAG,GGACTACNNGGGTATCTAAT

OB41	AGCATCTC,GTGAACCA	CCTAYGGGRBGCASCAG,GGACTACNNGGGTATCTAAT
OB42	CTGTAACC,TAGGCAAC	CCTAYGGGRBGCASCAG,GGACTACNNGGGTATCTAAT
OB43	GAATGACC,CATCCGAT	CCTAYGGGRBGCASCAG,GGACTACNNGGGTATCTAAT
OB44	CATTCGCA,CCTCATAG	CCTAYGGGRBGCASCAG,GGACTACNNGGGTATCTAAT
OB45	AGGTACCA,TTCGCAAC	CCTAYGGGRBGCASCAG,GGACTACNNGGGTATCTAAT
OB46	TAATCCGC,ATCAAGCG	CCTAYGGGRBGCASCAG,GGACTACNNGGGTATCTAAT
OB47	CGCTTCAA,TCCAGCAT	CCTAYGGGRBGCASCAG,GGACTACNNGGGTATCTAAT
OB48	AGCGATAC,ATCAGGCA	CCTAYGGGRBGCASCAG,GGACTACNNGGGTATCTAAT
OB49	CCAGTAAG,CTAACAGG	CCTAYGGGRBGCASCAG,GGACTACNNGGGTATCTAAT
OB4	CAAGTCGA,CACAAGGT	CCTAYGGGRBGCASCAG,GGACTACNNGGGTATCTAAT
OB50	GATTCACC,GCCATCTA	CCTAYGGGRBGCASCAG,GGACTACNNGGGTATCTAAT
OB51	GTAACACG,GACCAAGT	CCTAYGGGRBGCASCAG,GGACTACNNGGGTATCTAAT
OB52	GCCTATAC,CCAGGAAT	CCTAYGGGRBGCASCAG,GGACTACNNGGGTATCTAAT
OB53	CCATAGCT,GCCATGAA	CCTAYGGGRBGCASCAG,GGACTACNNGGGTATCTAAT
OB54	TACGCATC,AGACCTTC	CCTAYGGGRBGCASCAG,GGACTACNNGGGTATCTAAT
OB55	GCAAGCTA,CGACATAG	CCTAYGGGRBGCASCAG,GGACTACNNGGGTATCTAAT
OB56	GAGCAACT,AGACGTAC	CCTAYGGGRBGCASCAG,GGACTACNNGGGTATCTAAT
OB57	TATACGCC,CCGAATAG	CCTAYGGGRBGCASCAG,GGACTACNNGGGTATCTAAT
OB58	CAGTGAAC,CAATTCGC	CCTAYGGGRBGCASCAG,GGACTACNNGGGTATCTAAT
OB59	AGCCGATA,GGTCACAA	CCTAYGGGRBGCASCAG,GGACTACNNGGGTATCTAAT
OB5	AGCTACCT,TGCTACAC	CCTAYGGGRBGCASCAG,GGACTACNNGGGTATCTAAT
OB6	AGACATCG,AACCTGAG	CCTAYGGGRBGCASCAG,GGACTACNNGGGTATCTAAT
OB7	AGACTCCT,CTCTAGAC	CCTAYGGGRBGCASCAG,GGACTACNNGGGTATCTAAT
OB8	ACCTGTAC,GATAAGCC	CCTAYGGGRBGCASCAG,GGACTACNNGGGTATCTAAT
OB9	ACCTGTCA,AGACTTCC	CCTAYGGGRBGCASCAG,GGACTACNNGGGTATCTAAT
OC10	ACGCAGAT,ATCGGAAC	CCTAYGGGRBGCASCAG,GGACTACNNGGGTATCTAAT
OC11	TAACTCGC,ATCATCCG	CCTAYGGGRBGCASCAG,GGACTACNNGGGTATCTAAT
OC12	CACAGCTT,ATCCAGGA	CCTAYGGGRBGCASCAG,GGACTACNNGGGTATCTAAT
OC13	GGCACTAA,GCTTACAC	CCTAYGGGRBGCASCAG,GGACTACNNGGGTATCTAAT
OC14	CAGCAATG,GTGCAACA	CCTAYGGGRBGCASCAG,GGACTACNNGGGTATCTAAT

OC15	TACCAGAG,CCTATCAG	CCTAYGGGRBGCASCAG,GGACTACNNGGGTATCTAAT
OC16	CGGTAACA,ACCAGAGT	CCTAYGGGRBGCASCAG,GGACTACNNGGGTATCTAAT
OC17	ACGGATAC,GTCCACTA	CCTAYGGGRBGCASCAG,GGACTACNNGGGTATCTAAT
OC18	AAGTGACC,CCTTCAAG	CCTAYGGGRBGCASCAG,GGACTACNNGGGTATCTAAT
OC19	ATAACCGG,GCCAATGA	CCTAYGGGRBGCASCAG,GGACTACNNGGGTATCTAAT
OC1	ACTTAGCC,GAGCTACA	CCTAYGGGRBGCASCAG,GGACTACNNGGGTATCTAAT
OC20	AGCGAATC,ACGTGAAC	CCTAYGGGRBGCASCAG,GGACTACNNGGGTATCTAAT
OC21	GCCGTAAT,CCTGAGAA	CCTAYGGGRBGCASCAG,GGACTACNNGGGTATCTAAT
OC22	AGTCCGAA,TATACCGC	CCTAYGGGRBGCASCAG,GGACTACNNGGGTATCTAAT
OC23	TCGCCTAA,CACTAAGG	CCTAYGGGRBGCASCAG,GGACTACNNGGGTATCTAAT
OC24	AGACTCAG,ACAGAGTC	CCTAYGGGRBGCASCAG,GGACTACNNGGGTATCTAAT
OC25	GTCACAAG,CCTAGGAA	CCTAYGGGRBGCASCAG,GGACTACNNGGGTATCTAAT
OC26	ACAATGGC,ATGAACGC	CCTAYGGGRBGCASCAG,GGACTACNNGGGTATCTAAT
OC27	CAATGGCA,GCCTAACT	CCTAYGGGRBGCASCAG,GGACTACNNGGGTATCTAAT
OC29	CCAGATTC,CTTCACAG	CCTAYGGGRBGCASCAG,GGACTACNNGGGTATCTAAT
OC2	CCTGACAT,CCAGTCTA	CCTAYGGGRBGCASCAG,GGACTACNNGGGTATCTAAT
OC30	GGCATCAA,TTACACCG	CCTAYGGGRBGCASCAG,GGACTACNNGGGTATCTAAT
OC31	CAAGTCGA,CACAAGGT	CCTAYGGGRBGCASCAG,GGACTACNNGGGTATCTAAT
OC32	AGCTACCT,TGCTACAC	CCTAYGGGRBGCASCAG,GGACTACNNGGGTATCTAAT
OC33	AGACATCG,AACCTGAG	CCTAYGGGRBGCASCAG,GGACTACNNGGGTATCTAAT
OC34	ACCTGTAC,GATAAGCC	CCTAYGGGRBGCASCAG,GGACTACNNGGGTATCTAAT
OC35	ACCTGTCA,AGACTTCC	CCTAYGGGRBGCASCAG,GGACTACNNGGGTATCTAAT
OC36	TCCACTGA,ATCGCAAG	CCTAYGGGRBGCASCAG,GGACTACNNGGGTATCTAAT
OC37	TTCCGAAC,GGACAATC	CCTAYGGGRBGCASCAG,GGACTACNNGGGTATCTAAT
OC38	CATGATCC,GACACTTC	CCTAYGGGRBGCASCAG,GGACTACNNGGGTATCTAAT
OC39	CTAGAACG,GATACTCC	CCTAYGGGRBGCASCAG,GGACTACNNGGGTATCTAAT
OC3	CAGCATAG,CATGACCT	CCTAYGGGRBGCASCAG,GGACTACNNGGGTATCTAAT
OC40	AACTCCGT,TCAGGCAA	CCTAYGGGRBGCASCAG,GGACTACNNGGGTATCTAAT
OC41	GCAGATAC,ACACTGTC	CCTAYGGGRBGCASCAG,GGACTACNNGGGTATCTAAT
OC42	ATCGACCT,ACTCGAGA	CCTAYGGGRBGCASCAG,GGACTACNNGGGTATCTAAT

OC43	CTCACTGA,CGGACAAT	CCTAYGGGRBGCASCAG,GGACTACNNGGGTATCTAAT
OC44	ATAGGACC,GAAGATCC	CCTAYGGGRBGCASCAG,GGACTACNNGGGTATCTAAT
OC45	CAGCTACT,CTCAGTAC	CCTAYGGGRBGCASCAG,GGACTACNNGGGTATCTAAT
OC46	CCAATGTC,TCCACTAG	CCTAYGGGRBGCASCAG,GGACTACNNGGGTATCTAAT
OC47	ACGCAGAT,ATCGGAAC	CCTAYGGGRBGCASCAG,GGACTACNNGGGTATCTAAT
OC48	TAACTCGC,ATCATCCG	CCTAYGGGRBGCASCAG,GGACTACNNGGGTATCTAAT
OC49	CACAGCTT,ATCCAGGA	CCTAYGGGRBGCASCAG,GGACTACNNGGGTATCTAAT
OC4	GCTCACAT,ACCGATCT	CCTAYGGGRBGCASCAG,GGACTACNNGGGTATCTAAT
OC50	CAGCAATG,GTGCAACA	CCTAYGGGRBGCASCAG,GGACTACNNGGGTATCTAAT
OC51	CCTTACGA,ATACTGCC	CCTAYGGGRBGCASCAG,GGACTACNNGGGTATCTAAT
OC52	CGGTAACA,ACCAGAGT	CCTAYGGGRBGCASCAG,GGACTACNNGGGTATCTAAT
OC53	AGCGCTAA,TCTGCCAA	CCTAYGGGRBGCASCAG,GGACTACNNGGGTATCTAAT
OC54	AAGTGACC,CCTTCAAG	CCTAYGGGRBGCASCAG,GGACTACNNGGGTATCTAAT
OC55	AGATCCTC,GAACTTCC	CCTAYGGGRBGCASCAG,GGACTACNNGGGTATCTAAT
OC56	CATGACGA,TGAACAGC	CCTAYGGGRBGCASCAG,GGACTACNNGGGTATCTAAT
OC57	ATCGTACC,TACCTACG	CCTAYGGGRBGCASCAG,GGACTACNNGGGTATCTAAT
OC58	AACAGGTC,CGTTCACA	CCTAYGGGRBGCASCAG,GGACTACNNGGGTATCTAAT
OC59	TACAGTCC,CACGTTCA	CCTAYGGGRBGCASCAG,GGACTACNNGGGTATCTAAT
OC5	CTACACGT,CTCATGAC	CCTAYGGGRBGCASCAG,GGACTACNNGGGTATCTAAT
OC6	CTCACTGA,CGGACAAT	CCTAYGGGRBGCASCAG,GGACTACNNGGGTATCTAAT
OC7	ATAGGACC,GAAGATCC	CCTAYGGGRBGCASCAG,GGACTACNNGGGTATCTAAT
OC8	CAGCTACT,CTCAGTAC	CCTAYGGGRBGCASCAG,GGACTACNNGGGTATCTAAT
OC9	CCAATGTC,TCCACTAG	CCTAYGGGRBGCASCAG,GGACTACNNGGGTATCTAAT
VA10	CACAGCTT,ATCCAGGA	CCTAYGGGRBGCASCAG,GGACTACNNGGGTATCTAAT
VA11	CAGCAATG,GTGCAACA	CCTAYGGGRBGCASCAG,GGACTACNNGGGTATCTAAT
VA12	CCTTACGA,ATACTGCC	CCTAYGGGRBGCASCAG,GGACTACNNGGGTATCTAAT
VA13	CGGTAACA,ACCAGAGT	CCTAYGGGRBGCASCAG,GGACTACNNGGGTATCTAAT
VA14	ACGGATAC,GTCCACTA	CCTAYGGGRBGCASCAG,GGACTACNNGGGTATCTAAT
VA15	AAGTGACC,CCTTCAAG	CCTAYGGGRBGCASCAG,GGACTACNNGGGTATCTAAT
VA16	GCAGTAAC,ATTCACCG	CCTAYGGGRBGCASCAG,GGACTACNNGGGTATCTAAT

VA17	AGCGAATC,ACGTGAAC	CCTAYGGGRBGCASCAG,GGACTACNNGGGTATCTAAT
VA18	GCCGTAAT,CCTGAGAA	CCTAYGGGRBGCASCAG,GGACTACNNGGGTATCTAAT
VA19	AGTCCGAA,TATACCGC	CCTAYGGGRBGCASCAG,GGACTACNNGGGTATCTAAT
VA1	CTGTAACC,TAGGCAAC	CCTAYGGGRBGCASCAG,GGACTACNNGGGTATCTAAT
VA20	AGACTCAG,ACAGAGTC	CCTAYGGGRBGCASCAG,GGACTACNNGGGTATCTAAT
VA21	GTCACAAG,CCTAGGAA	CCTAYGGGRBGCASCAG,GGACTACNNGGGTATCTAAT
VA22	GACTGCAA,CAAGTCTC	CCTAYGGGRBGCASCAG,GGACTACNNGGGTATCTAAT
VA23	CGCTTCAA,TCCAGCAT	CCTAYGGGRBGCASCAG,GGACTACNNGGGTATCTAAT
VA24	CGGTACAA,CATGCAAG	CCTAYGGGRBGCASCAG,GGACTACNNGGGTATCTAAT
VA25	AGCGATAC,ATCAGGCA	CCTAYGGGRBGCASCAG,GGACTACNNGGGTATCTAAT
VA26	GGCATCAA,TTACACCG	CCTAYGGGRBGCASCAG,GGACTACNNGGGTATCTAAT
VA27	CAAGTCGA,CACAAGGT	CCTAYGGGRBGCASCAG,GGACTACNNGGGTATCTAAT
VA28	AGCTACCT,TGCTACAC	CCTAYGGGRBGCASCAG,GGACTACNNGGGTATCTAAT
VA29	AGACATCG,AACCTGAG	CCTAYGGGRBGCASCAG,GGACTACNNGGGTATCTAAT
VA2	CTACACGT,CTCATGAC	CCTAYGGGRBGCASCAG,GGACTACNNGGGTATCTAAT
VA30	ACCTGTAC,GATAAGCC	CCTAYGGGRBGCASCAG,GGACTACNNGGGTATCTAAT
VA31	ACCTGTCA,AGACTTCC	CCTAYGGGRBGCASCAG,GGACTACNNGGGTATCTAAT
VA32	TCCACTGA,ATCGCAAG	CCTAYGGGRBGCASCAG,GGACTACNNGGGTATCTAAT
VA33	TTCCGAAC,GGACAATC	CCTAYGGGRBGCASCAG,GGACTACNNGGGTATCTAAT
VA34	CATGATCC,GACACTTC	CCTAYGGGRBGCASCAG,GGACTACNNGGGTATCTAAT
VA35	CTAGAACG,GATACTCC	CCTAYGGGRBGCASCAG,GGACTACNNGGGTATCTAAT
VA36	AACTCCGT,TCAGGCAA	CCTAYGGGRBGCASCAG,GGACTACNNGGGTATCTAAT
VA37	GCAGATAC,ACACTGTC	CCTAYGGGRBGCASCAG,GGACTACNNGGGTATCTAAT
VA38	ATCGACCT,ACTCGAGA	CCTAYGGGRBGCASCAG,GGACTACNNGGGTATCTAAT
VA39	GCCTAAGA,GTACCAGA	CCTAYGGGRBGCASCAG,GGACTACNNGGGTATCTAAT
VA3	CTCACTGA,CGGACAAT	CCTAYGGGRBGCASCAG,GGACTACNNGGGTATCTAAT
VA40	CTTCCAGA,CTTCGAAC	CCTAYGGGRBGCASCAG,GGACTACNNGGGTATCTAAT
VA41	CGATCAAG,AACATGGC	CCTAYGGGRBGCASCAG,GGACTACNNGGGTATCTAAT
VA42	CCTAGCTA,CGATAACG	CCTAYGGGRBGCASCAG,GGACTACNNGGGTATCTAAT
VA43	ACGGACTA,GCTCAAGA	CCTAYGGGRBGCASCAG,GGACTACNNGGGTATCTAAT

VA44	AAGCTTCC,CCACTAGT	CCTAYGGGRBGCASCAG,GGACTACNNGGGTATCTAAT
VA45	AACGCTTC,CACTCAGT	CCTAYGGGRBGCASCAG,GGACTACNNGGGTATCTAAT
VA46	TACCAGAG,CCTATCAG	CCTAYGGGRBGCASCAG,GGACTACNNGGGTATCTAAT
VA47	ATAACCGG,GCCAATGA	CCTAYGGGRBGCASCAG,GGACTACNNGGGTATCTAAT
VA48	ACAATGGC,ATGAACGC	CCTAYGGGRBGCASCAG,GGACTACNNGGGTATCTAAT
VA49	CAATGGCA,GCCTAACT	CCTAYGGGRBGCASCAG,GGACTACNNGGGTATCTAAT
VA4	ATAGGACC,GAAGATCC	CCTAYGGGRBGCASCAG,GGACTACNNGGGTATCTAAT
VA50	AGATCCTC,GAACTTCC	CCTAYGGGRBGCASCAG,GGACTACNNGGGTATCTAAT
VA51	AAGCGTCA,ATACGGCA	CCTAYGGGRBGCASCAG,GGACTACNNGGGTATCTAAT
VA52	AGCCACTT,CTAGCGAA	CCTAYGGGRBGCASCAG,GGACTACNNGGGTATCTAAT
VA53	TCGCCTAA,CACTAAGG	CCTAYGGGRBGCASCAG,GGACTACNNGGGTATCTAAT
VA54	GGCACTAA,GCTTACAC	CCTAYGGGRBGCASCAG,GGACTACNNGGGTATCTAAT
VA55	GTAACAGC,ACGTACCT	CCTAYGGGRBGCASCAG,GGACTACNNGGGTATCTAAT
VA56	TAAGCAGC,ACGCCTAT	CCTAYGGGRBGCASCAG,GGACTACNNGGGTATCTAAT
VA57	CCGAGTAA,TCAACGGA	CCTAYGGGRBGCASCAG,GGACTACNNGGGTATCTAAT
VA58	GACAAGCT,ATGAGACC	CCTAYGGGRBGCASCAG,GGACTACNNGGGTATCTAAT
VA59	CTGTAACC,TAGGCAAC	CCTAYGGGRBGCASCAG,GGACTACNNGGGTATCTAAT
VA5	CAGCTACT,CTCAGTAC	CCTAYGGGRBGCASCAG,GGACTACNNGGGTATCTAAT
VA6	CCAATGTC,TCCACTAG	CCTAYGGGRBGCASCAG,GGACTACNNGGGTATCTAAT
VA7	ACGCAGAT,ATCGGAAC	CCTAYGGGRBGCASCAG,GGACTACNNGGGTATCTAAT
VA8	TAACTCGC,ATCATCCG	CCTAYGGGRBGCASCAG,GGACTACNNGGGTATCTAAT
VA9	CACGCTAT,TGCGACAA	CCTAYGGGRBGCASCAG,GGACTACNNGGGTATCTAAT
VB10	TACAGTCC,CACGTTCA	CCTAYGGGRBGCASCAG,GGACTACNNGGGTATCTAAT
VB11	ACGTCCAT,AGAAGTCC	CCTAYGGGRBGCASCAG,GGACTACNNGGGTATCTAAT
VB12	ACTTAGCC,GAGCTACA	CCTAYGGGRBGCASCAG,GGACTACNNGGGTATCTAAT
VB13	CCTGACAT,CCAGTCTA	CCTAYGGGRBGCASCAG,GGACTACNNGGGTATCTAAT
VB14	CAGCATAG,CATGACCT	CCTAYGGGRBGCASCAG,GGACTACNNGGGTATCTAAT
VB15	GCTCACAT,ACCGATCT	CCTAYGGGRBGCASCAG,GGACTACNNGGGTATCTAAT
VB16	CTACACGT,CTCATGAC	CCTAYGGGRBGCASCAG,GGACTACNNGGGTATCTAAT
VB17	CTCACTGA,CGGACAAT	CCTAYGGGRBGCASCAG,GGACTACNNGGGTATCTAAT

VB18	ATAGGACC,GAAGATCC	CCTAYGGGRBGCASCAG,GGACTACNNGGGTATCTAAT
VB19	CAGCTACT,CTCAGTAC	CCTAYGGGRBGCASCAG,GGACTACNNGGGTATCTAAT
VB1	ATTGCACC,TTGACACC	CCTAYGGGRBGCASCAG,GGACTACNNGGGTATCTAAT
VB20	CCAATGTC,TCCACTAG	CCTAYGGGRBGCASCAG,GGACTACNNGGGTATCTAAT
VB21	ACGCAGAT,ATCGGAAC	CCTAYGGGRBGCASCAG,GGACTACNNGGGTATCTAAT
VB22	TAACTCGC,ATCATCCG	CCTAYGGGRBGCASCAG,GGACTACNNGGGTATCTAAT
VB23	CACAGCTT,ATCCAGGA	CCTAYGGGRBGCASCAG,GGACTACNNGGGTATCTAAT
VB24	CAGCAATG,GTGCAACA	CCTAYGGGRBGCASCAG,GGACTACNNGGGTATCTAAT
VB25	CCTTACGA,ATACTGCC	CCTAYGGGRBGCASCAG,GGACTACNNGGGTATCTAAT
VB26	CGGTAACA,ACCAGAGT	CCTAYGGGRBGCASCAG,GGACTACNNGGGTATCTAAT
VB27	ACGGATAC,GTCCACTA	CCTAYGGGRBGCASCAG,GGACTACNNGGGTATCTAAT
VB29	GCAGTAAC,ATTCACCG	CCTAYGGGRBGCASCAG,GGACTACNNGGGTATCTAAT
VB2	AGGCTACA,TACCACTG	CCTAYGGGRBGCASCAG,GGACTACNNGGGTATCTAAT
VB30	AGCGAATC,ACGTGAAC	CCTAYGGGRBGCASCAG,GGACTACNNGGGTATCTAAT
VB31	GCCGTAAT,CCTGAGAA	CCTAYGGGRBGCASCAG,GGACTACNNGGGTATCTAAT
VB32	AGTCCGAA,TATACCGC	CCTAYGGGRBGCASCAG,GGACTACNNGGGTATCTAAT
VB33	AGACTCAG,ACAGAGTC	CCTAYGGGRBGCASCAG,GGACTACNNGGGTATCTAAT
VB34	GTCACAAG,CCTAGGAA	CCTAYGGGRBGCASCAG,GGACTACNNGGGTATCTAAT
VB35	TAAGCCTC,TCCGAAGA	CCTAYGGGRBGCASCAG,GGACTACNNGGGTATCTAAT
VB36	CACTGAAG,AGCACTCT	CCTAYGGGRBGCASCAG,GGACTACNNGGGTATCTAAT
VB37	CGGTACAA,CATGCAAG	CCTAYGGGRBGCASCAG,GGACTACNNGGGTATCTAAT
VB38	CCAGATTC,CTTCACAG	CCTAYGGGRBGCASCAG,GGACTACNNGGGTATCTAAT
VB39	GGCATCAA,TTACACCG	CCTAYGGGRBGCASCAG,GGACTACNNGGGTATCTAAT
VB3	AGGACCTA,CGTATCAC	CCTAYGGGRBGCASCAG,GGACTACNNGGGTATCTAAT
VB40	CAAGTCGA,CACAAGGT	CCTAYGGGRBGCASCAG,GGACTACNNGGGTATCTAAT
VB41	AGCTACCT,TGCTACAC	CCTAYGGGRBGCASCAG,GGACTACNNGGGTATCTAAT
VB42	AGACATCG,AACCTGAG	CCTAYGGGRBGCASCAG,GGACTACNNGGGTATCTAAT
VB43	ACCTGTAC,GATAAGCC	CCTAYGGGRBGCASCAG,GGACTACNNGGGTATCTAAT
VB44	ACCTGTCA,AGACTTCC	CCTAYGGGRBGCASCAG,GGACTACNNGGGTATCTAAT
VB45	TCCACTGA,ATCGCAAG	CCTAYGGGRBGCASCAG,GGACTACNNGGGTATCTAAT

VB46	AGCATCTC,GTGAACCA	CCTAYGGGRBGCASCAG,GGACTACNNGGGTATCTAAT
VB47	CATGATCC,GACACTTC	CCTAYGGGRBGCASCAG,GGACTACNNGGGTATCTAAT
VB48	CTAGAACG,GATACTCC	CCTAYGGGRBGCASCAG,GGACTACNNGGGTATCTAAT
VB49	AACTCCGT,TCAGGCAA	CCTAYGGGRBGCASCAG,GGACTACNNGGGTATCTAAT
VB4	CAATGCAG,GAACAGCT	CCTAYGGGRBGCASCAG,GGACTACNNGGGTATCTAAT
VB50	GCAGATAC,ACACTGTC	CCTAYGGGRBGCASCAG,GGACTACNNGGGTATCTAAT
VB51	ATCGACCT,ACTCGAGA	CCTAYGGGRBGCASCAG,GGACTACNNGGGTATCTAAT
VB52	GTAACACG,GACCAAGT	CCTAYGGGRBGCASCAG,GGACTACNNGGGTATCTAAT
VB53	GCCTATAC,CCAGGAAT	CCTAYGGGRBGCASCAG,GGACTACNNGGGTATCTAAT
VB54	CCATAGCT,GCCATGAA	CCTAYGGGRBGCASCAG,GGACTACNNGGGTATCTAAT
VB55	AGCGATAC,ATCAGGCA	CCTAYGGGRBGCASCAG,GGACTACNNGGGTATCTAAT
VB56	GCAAGCTA,CGACATAG	CCTAYGGGRBGCASCAG,GGACTACNNGGGTATCTAAT
VB57	CCTTACGA,ATACTGCC	CCTAYGGGRBGCASCAG,GGACTACNNGGGTATCTAAT
VB58	CGGTAACA,ACCAGAGT	CCTAYGGGRBGCASCAG,GGACTACNNGGGTATCTAAT
VB59	CCAGTAAG,CTAACAGG	CCTAYGGGRBGCASCAG,GGACTACNNGGGTATCTAAT
VB5	CTCTAAGC,CATCTAGC	CCTAYGGGRBGCASCAG,GGACTACNNGGGTATCTAAT
VB6	CATGACGA,TGAACAGC	CCTAYGGGRBGCASCAG,GGACTACNNGGGTATCTAAT
VB7	TAAGCAGC,ACGCCTAT	CCTAYGGGRBGCASCAG,GGACTACNNGGGTATCTAAT
VB8	ATCGTACC,TACCTACG	CCTAYGGGRBGCASCAG,GGACTACNNGGGTATCTAAT
VB9	AACAGGTC,CGTTCACA	CCTAYGGGRBGCASCAG,GGACTACNNGGGTATCTAAT
VC10	CCAATGTC,TCCACTAG	CCTAYGGGRBGCASCAG,GGACTACNNGGGTATCTAAT
VC11	ACGCAGAT,ATCGGAAC	CCTAYGGGRBGCASCAG,GGACTACNNGGGTATCTAAT
VC12	TAACTCGC,ATCATCCG	CCTAYGGGRBGCASCAG,GGACTACNNGGGTATCTAAT
VC13	CACAGCTT,ATCCAGGA	CCTAYGGGRBGCASCAG,GGACTACNNGGGTATCTAAT
VC14	CGATCAAG,AACATGGC	CCTAYGGGRBGCASCAG,GGACTACNNGGGTATCTAAT
VC15	CCTAGCTA,CGATAACG	CCTAYGGGRBGCASCAG,GGACTACNNGGGTATCTAAT
VC16	ACGGACTA,GCTCAAGA	CCTAYGGGRBGCASCAG,GGACTACNNGGGTATCTAAT
VC17	AAGCTTCC,CCACTAGT	CCTAYGGGRBGCASCAG,GGACTACNNGGGTATCTAAT
VC18	AACGCTTC,CACTCAGT	CCTAYGGGRBGCASCAG,GGACTACNNGGGTATCTAAT
VC19	TACCAGAG,CCTATCAG	CCTAYGGGRBGCASCAG,GGACTACNNGGGTATCTAAT

VC1	ACGTCCAT,AGAAGTCC	CCTAYGGGRBGCASCAG,GGACTACNNGGGTATCTAAT
VC20	ATAACCGG,GCCAATGA	CCTAYGGGRBGCASCAG,GGACTACNNGGGTATCTAAT
VC21	ACAATGGC,ATGAACGC	CCTAYGGGRBGCASCAG,GGACTACNNGGGTATCTAAT
VC22	CAATGGCA,GCCTAACT	CCTAYGGGRBGCASCAG,GGACTACNNGGGTATCTAAT
VC23	AGATCCTC,GAACTTCC	CCTAYGGGRBGCASCAG,GGACTACNNGGGTATCTAAT
VC24	AAGCGTCA,ATACGGCA	CCTAYGGGRBGCASCAG,GGACTACNNGGGTATCTAAT
VC25	AGCCACTT,CTAGCGAA	CCTAYGGGRBGCASCAG,GGACTACNNGGGTATCTAAT
VC26	TCGCCTAA,CACTAAGG	CCTAYGGGRBGCASCAG,GGACTACNNGGGTATCTAAT
VC27	GGCACTAA,GCTTACAC	CCTAYGGGRBGCASCAG,GGACTACNNGGGTATCTAAT
VC29	TAAGCAGC,ACGCCTAT	CCTAYGGGRBGCASCAG,GGACTACNNGGGTATCTAAT
VC2	ACTTAGCC,GAGCTACA	CCTAYGGGRBGCASCAG,GGACTACNNGGGTATCTAAT
VC30	CCGAGTAA,TCAACGGA	CCTAYGGGRBGCASCAG,GGACTACNNGGGTATCTAAT
VC31	GACAAGCT,ATGAGACC	CCTAYGGGRBGCASCAG,GGACTACNNGGGTATCTAAT
VC32	CTGTAACC,TAGGCAAC	CCTAYGGGRBGCASCAG,GGACTACNNGGGTATCTAAT
VC33	TAATCCGC,ATCAAGCG	CCTAYGGGRBGCASCAG,GGACTACNNGGGTATCTAAT
VC34	TACGCATC,AGACCTTC	CCTAYGGGRBGCASCAG,GGACTACNNGGGTATCTAAT
VC35	ATTGCACC,TTGACACC	CCTAYGGGRBGCASCAG,GGACTACNNGGGTATCTAAT
VC36	TCAAGCGA,ACGAGTAC	CCTAYGGGRBGCASCAG,GGACTACNNGGGTATCTAAT
VC37	AGCATCTC,GTGAACCA	CCTAYGGGRBGCASCAG,GGACTACNNGGGTATCTAAT
VC38	CCAGTAAGAGA,CTAACAGGACGA	CCTAYGGGRBGCASCAG,GGACTACNNGGGTATCTAAT
VC39	GAATGACC,CATCCGAT	CCTAYGGGRBGCASCAG,GGACTACNNGGGTATCTAAT
VC3	CCTGACAT,CCAGTCTA	CCTAYGGGRBGCASCAG,GGACTACNNGGGTATCTAAT
VC40	CATTCGCA,CCTCATAG	CCTAYGGGRBGCASCAG,GGACTACNNGGGTATCTAAT
VC41	AGGTACCA,TTCGCAAC	CCTAYGGGRBGCASCAG,GGACTACNNGGGTATCTAAT
VC42	GACTGCAA,CAAGTCTC	CCTAYGGGRBGCASCAG,GGACTACNNGGGTATCTAAT
VC43	CGCTTCAA,TCCAGCAT	CCTAYGGGRBGCASCAG,GGACTACNNGGGTATCTAAT
VC44	AGCGATAC,ATCAGGCA	CCTAYGGGRBGCASCAG,GGACTACNNGGGTATCTAAT
VC45	CCAGTAAG,CTAACAGG	CCTAYGGGRBGCASCAG,GGACTACNNGGGTATCTAAT
VC46	GATTCACC,GCCATCTA	CCTAYGGGRBGCASCAG,GGACTACNNGGGTATCTAAT
VC47	CTGCATCA,CGAACTTC	CCTAYGGGRBGCASCAG,GGACTACNNGGGTATCTAAT

VC48	GTAACACG,GACCAAGT	CCTAYGGGRBGCASCAG,GGACTACNNGGGTATCTAAT
VC49	GCCTATAC,CCAGGAAT	CCTAYGGGRBGCASCAG,GGACTACNNGGGTATCTAAT
VC4	CAGCATAG,CATGACCT	CCTAYGGGRBGCASCAG,GGACTACNNGGGTATCTAAT
VC50	CCATAGCT,GCCATGAA	CCTAYGGGRBGCASCAG,GGACTACNNGGGTATCTAAT
VC51	CCATGCAT,CTCGCAAT	CCTAYGGGRBGCASCAG,GGACTACNNGGGTATCTAAT
VC52	GCAAGCTA,CGACATAG	CCTAYGGGRBGCASCAG,GGACTACNNGGGTATCTAAT
VC53	GAGCAACT,AGACGTAC	CCTAYGGGRBGCASCAG,GGACTACNNGGGTATCTAAT
VC54	GATTCACC,GCCATCTA	CCTAYGGGRBGCASCAG,GGACTACNNGGGTATCTAAT
VC55	AAGCGTCA,ATACGGCA	CCTAYGGGRBGCASCAG,GGACTACNNGGGTATCTAAT
VC56	CTGCATCA,CGAACTTC	CCTAYGGGRBGCASCAG,GGACTACNNGGGTATCTAAT
VC57	GTAACACG,GACCAAGT	CCTAYGGGRBGCASCAG,GGACTACNNGGGTATCTAAT
VC58	CCATATCGAGACT,CTAGAGACG	CCTAYGGGRBGCASCAG,GGACTACNNGGGTATCTAAT
VC59	AAGCGTCA,ATACGGCA	CCTAYGGGRBGCASCAG,GGACTACNNGGGTATCTAAT
VC5	GCTCACAT,ACCGATCT	CCTAYGGGRBGCASCAG,GGACTACNNGGGTATCTAAT
VC6	CTACACGT,CTCATGAC	CCTAYGGGRBGCASCAG,GGACTACNNGGGTATCTAAT
VC7	CTCACTGA,CGGACAAT	CCTAYGGGRBGCASCAG,GGACTACNNGGGTATCTAAT
VC8	ATAGGACC,GAAGATCC	CCTAYGGGRBGCASCAG,GGACTACNNGGGTATCTAAT
VC9	CAGCTACT,CTCAGTAC	CCTAYGGGRBGCASCAG,GGACTACNNGGGTATCTAAT

Supplemental material 1. This table shows every sample in our study with its primers and unique barcode.
