# Supplementary material for: Will gut, oral, and vaginal microbiota influence the outcome of FET or be influenced by FET? A pilot study
Source: mBio. 2025 Jun 17;16(7):e00509-25. doi: 10.1128/mbio.00509-25 (PMC12239586; doi:10.1128/mbio.00509-25)
Supplement: File S2 — Statistical results obtained at each step of the data processing. [file mbio.00509-25-s0002.docx]

| Sample | RawPE | Combined | Qualified | Nochime | Base(nt) | Avglen(nt) | GC | Q20 | Q30 |
| --- | --- | --- | --- | --- | --- | --- | --- | --- | --- |
| OA1 | 156600 | 153900 | 148545 | 83469 | 34828296 | 417.26 | 50.89% | 98.11% | 94.00% |
| OA2 | 76336 | 75771 | 74822 | 71996 | 30691669 | 426.3 | 51.69% | 98.87% | 95.88% |
| OA3 | 104995 | 104095 | 102677 | 98683 | 41972732 | 425.33 | 51.55% | 98.68% | 95.29% |
| OA4 | 104137 | 103164 | 101414 | 92115 | 39282709 | 426.45 | 51.69% | 98.64% | 95.27% |
| OA5 | 103313 | 102260 | 100660 | 89710 | 38031063 | 423.93 | 51.42% | 98.70% | 95.44% |
| OA6 | 59858 | 59346 | 58342 | 52281 | 22063960 | 422.03 | 52.63% | 98.69% | 95.44% |
| OA7 | 105445 | 104360 | 102863 | 100476 | 42870075 | 426.67 | 51.88% | 98.60% | 95.12% |
| OA8 | 103639 | 102544 | 101124 | 94592 | 39979406 | 422.65 | 51.45% | 98.74% | 95.52% |
| OA9 | 186031 | 182431 | 175927 | 111258 | 47118936 | 423.51 | 51.63% | 97.96% | 93.53% |
| OA10 | 112183 | 111193 | 109690 | 101439 | 43045993 | 424.35 | 52.13% | 98.80% | 95.71% |
| OA11 | 105400 | 104486 | 103017 | 96255 | 41099796 | 426.99 | 52.27% | 98.84% | 95.82% |
| OA12 | 104125 | 102763 | 101050 | 97378 | 41280350 | 423.92 | 51.61% | 98.69% | 95.38% |
| OA13 | 103164 | 102194 | 100718 | 97245 | 41475530 | 426.51 | 52.60% | 98.77% | 95.64% |
| OA14 | 119055 | 117722 | 116050 | 112898 | 47729266 | 422.76 | 51.19% | 98.63% | 95.19% |
| OA15 | 103295 | 102201 | 100479 | 95027 | 40511833 | 426.32 | 51.53% | 98.67% | 95.35% |
| OA16 | 103798 | 102726 | 101241 | 97648 | 41091324 | 420.81 | 51.88% | 98.74% | 95.60% |
| OA17 | 83222 | 82450 | 81330 | 75411 | 32080885 | 425.41 | 51.92% | 98.83% | 95.82% |
| OA18 | 100478 | 99581 | 98317 | 92899 | 39448717 | 424.64 | 51.51% | 98.87% | 95.89% |
| OA19 | 125489 | 123436 | 120340 | 114881 | 48777699 | 424.59 | 51.82% | 98.41% | 94.68% |
| OA20 | 142159 | 140196 | 136690 | 131495 | 55735106 | 423.86 | 51.21% | 98.33% | 94.44% |
| OA21 | 68664 | 68036 | 67074 | 63286 | 26973337 | 426.21 | 52.37% | 98.82% | 95.76% |
| OA22 | 109132 | 108150 | 106638 | 101207 | 43088087 | 425.74 | 52.33% | 98.78% | 95.65% |
| OA23 | 102570 | 101559 | 100222 | 95070 | 40335000 | 424.27 | 51.19% | 98.79% | 95.69% |
| OA24 | 105566 | 104505 | 102788 | 101317 | 43212569 | 426.51 | 52.43% | 98.72% | 95.49% |
| OA25 | 102467 | 101411 | 99880 | 99186 | 42089904 | 424.35 | 51.60% | 98.70% | 95.44% |
| OA26 | 54761 | 54255 | 53451 | 52770 | 22288823 | 422.38 | 51.41% | 98.69% | 95.41% |
| OA27 | 105520 | 104598 | 103108 | 87268 | 37333397 | 427.8 | 52.97% | 98.86% | 95.91% |
| OA28 | 103724 | 102775 | 101432 | 98676 | 41906824 | 424.69 | 51.87% | 98.88% | 95.93% |
| OA29 | 106434 | 105668 | 104275 | 101240 | 43358552 | 428.27 | 52.91% | 98.92% | 96.09% |
| OA30 | 104495 | 103600 | 102238 | 100558 | 42846593 | 426.09 | 51.92% | 98.74% | 95.55% |
| OA31 | 70667 | 70036 | 69096 | 68771 | 29059237 | 422.55 | 51.94% | 98.78% | 95.67% |
| OA32 | 106443 | 105384 | 103916 | 100687 | 42699095 | 424.08 | 51.38% | 98.72% | 95.46% |
| OA33 | 106385 | 105406 | 103765 | 100296 | 42700345 | 425.74 | 51.88% | 98.76% | 95.59% |
| OA34 | 103876 | 102749 | 101033 | 100623 | 43072734 | 428.06 | 51.73% | 98.70% | 95.49% |
| OA35 | 106176 | 105074 | 103628 | 85750 | 35953305 | 419.28 | 52.83% | 98.81% | 95.73% |
| OA36 | 99426 | 98561 | 97263 | 96350 | 41101942 | 426.59 | 51.63% | 98.75% | 95.62% |
| OA37 | 113572 | 112629 | 111040 | 100579 | 42921905 | 426.75 | 52.21% | 98.76% | 95.59% |
| OA38 | 70785 | 70144 | 69232 | 68332 | 29129597 | 426.3 | 52.08% | 98.73% | 95.54% |
| OA39 | 102879 | 102017 | 100653 | 97995 | 41699381 | 425.53 | 51.57% | 98.80% | 95.71% |
| OA40 | 108258 | 107201 | 105725 | 104004 | 43948705 | 422.57 | 51.54% | 98.77% | 95.59% |
| OA41 | 76644 | 75960 | 74951 | 73067 | 30824021 | 421.86 | 50.55% | 98.89% | 95.97% |
| OA42 | 103884 | 102859 | 101306 | 94601 | 40158760 | 424.51 | 53.10% | 98.75% | 95.60% |
| OA43 | 63344 | 62950 | 62247 | 58465 | 25037005 | 428.24 | 53.09% | 98.89% | 96.00% |
| OA44 | 69577 | 69054 | 68093 | 63351 | 27145532 | 428.49 | 53.33% | 98.94% | 96.20% |
| OA45 | 119195 | 117799 | 116250 | 111387 | 46942091 | 421.43 | 50.41% | 98.78% | 95.61% |
| OA46 | 105605 | 104496 | 102795 | 97187 | 41315746 | 425.12 | 51.58% | 98.73% | 95.52% |
| OA47 | 118870 | 117852 | 116261 | 115975 | 49363098 | 425.64 | 52.22% | 98.86% | 95.90% |
| OA48 | 104389 | 102910 | 101230 | 95775 | 40543716 | 423.32 | 51.55% | 98.46% | 94.68% |
| OA49 | 103318 | 102386 | 100837 | 99334 | 42269333 | 425.53 | 51.84% | 98.69% | 95.41% |
| OA50 | 106399 | 105680 | 104580 | 103193 | 43853035 | 424.96 | 51.36% | 98.96% | 96.11% |
| OA51 | 104056 | 102965 | 101537 | 100218 | 42561648 | 424.69 | 52.69% | 98.73% | 95.51% |
| OA52 | 106984 | 106194 | 104772 | 97995 | 41934729 | 427.93 | 52.89% | 98.84% | 95.91% |
| OA53 | 103372 | 102644 | 101323 | 87054 | 37271705 | 428.14 | 52.97% | 98.93% | 96.10% |
| OA54 | 99009 | 98369 | 97203 | 93166 | 39911480 | 428.39 | 53.05% | 98.93% | 96.12% |
| OA55 | 103740 | 103014 | 101778 | 98240 | 42039819 | 427.93 | 52.65% | 98.89% | 95.98% |
| OA56 | 66719 | 66213 | 65071 | 51480 | 22031941 | 427.97 | 52.81% | 98.69% | 95.46% |
| OA57 | 119308 | 118273 | 116854 | 111984 | 46895004 | 418.77 | 53.43% | 98.83% | 95.76% |
| OA58 | 104153 | 103310 | 102054 | 100681 | 42773618 | 424.84 | 52.27% | 98.86% | 95.85% |
| OA59 | 102500 | 101589 | 100348 | 99321 | 41915633 | 422.02 | 54.12% | 98.85% | 95.88% |
| VA1 | 207605 | 203738 | 196406 | 110815 | 46321499 | 418.01 | 50.96% | 97.99% | 93.61% |
| VA2 | 103208 | 102494 | 101110 | 99466 | 41924597 | 421.5 | 53.72% | 98.83% | 95.68% |
| VA3 | 103748 | 102904 | 101237 | 95578 | 41000421 | 428.97 | 51.17% | 98.75% | 95.48% |
| VA4 | 106023 | 105159 | 103442 | 103002 | 44179307 | 428.92 | 50.38% | 98.72% | 95.41% |
| VA5 | 102959 | 102119 | 100537 | 97818 | 41944684 | 428.8 | 50.98% | 98.81% | 95.65% |
| VA6 | 106059 | 105361 | 103770 | 103640 | 44458064 | 428.97 | 51.50% | 98.85% | 95.91% |
| VA7 | 85688 | 84957 | 82974 | 82842 | 35533976 | 428.94 | 51.56% | 98.47% | 94.83% |
| VA8 | 103058 | 102297 | 100918 | 100173 | 41879917 | 418.08 | 54.45% | 98.84% | 95.76% |
| VA9 | 122447 | 120059 | 115861 | 62424 | 25962390 | 415.9 | 51.74% | 98.01% | 93.67% |
| VA10 | 104990 | 104227 | 102629 | 100634 | 42686294 | 424.17 | 52.10% | 98.76% | 95.59% |
| VA11 | 124825 | 122946 | 119991 | 119330 | 50761723 | 425.39 | 51.14% | 98.35% | 94.47% |
| VA12 | 106762 | 105994 | 104389 | 99995 | 42881414 | 428.84 | 51.24% | 98.87% | 95.88% |
| VA13 | 102743 | 101934 | 100756 | 100017 | 41512901 | 415.06 | 56.63% | 98.79% | 95.86% |
| VA14 | 139501 | 136862 | 132614 | 131505 | 56390694 | 428.81 | 51.49% | 98.02% | 93.46% |
| VA15 | 106287 | 105388 | 104168 | 103311 | 43189394 | 418.05 | 53.55% | 98.84% | 95.88% |
| VA16 | 133802 | 132114 | 128694 | 120042 | 51485589 | 428.9 | 51.25% | 98.26% | 94.25% |
| VA17 | 97240 | 96441 | 94903 | 92137 | 39512689 | 428.85 | 51.47% | 98.79% | 95.66% |
| VA18 | 100096 | 99473 | 98209 | 96537 | 41411791 | 428.97 | 51.13% | 98.92% | 96.04% |
| VA19 | 103458 | 102725 | 101190 | 101011 | 43322458 | 428.89 | 50.95% | 98.81% | 95.69% |
| VA20 | 106103 | 105311 | 103583 | 103561 | 44423361 | 428.96 | 51.45% | 98.78% | 95.66% |
| VA21 | 102813 | 101624 | 100139 | 99968 | 42873727 | 428.87 | 51.74% | 98.67% | 95.30% |
| VA22 | 85246 | 84690 | 83647 | 76808 | 31848400 | 414.65 | 56.02% | 98.87% | 95.89% |
| VA23 | 67149 | 66522 | 65524 | 51605 | 22137574 | 428.98 | 51.74% | 98.79% | 95.61% |
| VA24 | 82530 | 81972 | 80850 | 80652 | 34597725 | 428.98 | 51.55% | 98.82% | 95.79% |
| VA25 | 104356 | 103572 | 101880 | 86687 | 37185430 | 428.96 | 51.32% | 98.81% | 95.77% |
| VA26 | 117254 | 116448 | 114872 | 114808 | 49251860 | 428.99 | 51.46% | 98.88% | 95.96% |
| VA27 | 117766 | 116921 | 115165 | 115126 | 49384834 | 428.96 | 51.52% | 98.84% | 95.79% |
| VA28 | 104604 | 103882 | 102189 | 101892 | 43708794 | 428.97 | 51.39% | 98.70% | 95.37% |
| VA29 | 104235 | 103445 | 101792 | 101761 | 43648078 | 428.93 | 51.37% | 98.84% | 95.86% |
| VA30 | 105289 | 104584 | 103210 | 103026 | 44181100 | 428.83 | 51.61% | 98.92% | 96.08% |
| VA31 | 104073 | 103351 | 101927 | 101905 | 43715722 | 428.99 | 51.52% | 98.95% | 96.12% |
| VA32 | 96049 | 95345 | 93958 | 93629 | 40151292 | 428.83 | 51.61% | 98.93% | 96.09% |
| VA33 | 103825 | 103148 | 101654 | 101227 | 43422747 | 428.96 | 51.07% | 98.80% | 95.62% |
| VA34 | 110165 | 109459 | 108034 | 108014 | 46335849 | 428.98 | 51.49% | 98.88% | 95.96% |
| VA35 | 104275 | 103564 | 102069 | 100582 | 43148971 | 428.99 | 51.47% | 98.88% | 95.92% |
| VA36 | 104784 | 104036 | 102497 | 102356 | 43909118 | 428.98 | 51.52% | 98.86% | 95.92% |
| VA37 | 139232 | 137984 | 135293 | 134106 | 55500890 | 413.86 | 55.77% | 98.62% | 95.23% |
| VA38 | 64241 | 63689 | 62904 | 60806 | 25641537 | 421.69 | 53.08% | 98.84% | 95.87% |
| VA39 | 88511 | 87918 | 85981 | 85960 | 36876129 | 428.99 | 51.52% | 98.80% | 95.75% |
| VA40 | 103194 | 102030 | 99836 | 98916 | 42312399 | 427.76 | 51.49% | 98.65% | 95.31% |
| VA41 | 106727 | 105722 | 103080 | 102784 | 44053570 | 428.6 | 51.63% | 98.65% | 95.33% |
| VA42 | 115256 | 114283 | 111551 | 111287 | 47736403 | 428.95 | 51.02% | 98.70% | 95.40% |
| VA43 | 69382 | 68832 | 67157 | 67127 | 28792868 | 428.93 | 51.53% | 98.66% | 95.40% |
| VA44 | 104095 | 103146 | 101444 | 95075 | 40777293 | 428.9 | 51.21% | 98.57% | 95.02% |
| VA45 | 102181 | 101302 | 98802 | 97779 | 41944203 | 428.97 | 50.60% | 98.59% | 95.09% |
| VA46 | 104023 | 102340 | 99875 | 99727 | 42757643 | 428.75 | 51.74% | 98.53% | 94.90% |
| VA47 | 104062 | 102639 | 99941 | 98885 | 42359106 | 428.37 | 53.08% | 98.48% | 94.84% |
| VA48 | 103323 | 102279 | 99764 | 99515 | 42554807 | 427.62 | 51.05% | 98.69% | 95.31% |
| VA49 | 75969 | 75238 | 73436 | 73148 | 31220729 | 426.82 | 51.07% | 98.74% | 95.59% |
| VA50 | 102259 | 101263 | 98638 | 98497 | 42241093 | 428.86 | 51.10% | 98.49% | 94.74% |
| VA51 | 104312 | 103354 | 100716 | 95623 | 41021762 | 428.99 | 51.31% | 98.71% | 95.45% |
| VA52 | 105579 | 104575 | 101975 | 100879 | 43269764 | 428.93 | 50.71% | 98.60% | 95.10% |
| VA53 | 93792 | 93100 | 91039 | 90804 | 38948596 | 428.93 | 51.05% | 98.81% | 95.76% |
| VA54 | 102968 | 102074 | 99686 | 99662 | 42753962 | 428.99 | 51.75% | 98.71% | 95.49% |
| VA55 | 138115 | 136162 | 132661 | 129041 | 53648564 | 415.75 | 54.99% | 98.44% | 94.82% |
| VA56 | 106260 | 105283 | 102737 | 102725 | 44068395 | 428.99 | 51.52% | 98.70% | 95.50% |
| VA57 | 85209 | 84533 | 82936 | 82097 | 34090640 | 415.25 | 53.62% | 98.85% | 95.99% |
| VA58 | 104169 | 103078 | 100484 | 100400 | 43069878 | 428.98 | 51.72% | 98.64% | 95.28% |
| VA59 | 106524 | 105749 | 103847 | 103661 | 42673578 | 411.66 | 56.98% | 98.82% | 95.74% |
| GA1 | 252790 | 248191 | 239566 | 148129 | 61857610 | 417.59 | 50.68% | 98.05% | 93.71% |
| GA2 | 109029 | 107673 | 105407 | 96556 | 40053624 | 414.82 | 50.62% | 98.73% | 95.57% |
| GA3 | 103234 | 102179 | 100281 | 95477 | 39841949 | 417.29 | 51.40% | 98.86% | 95.96% |
| GA4 | 78400 | 77703 | 76294 | 75547 | 32024582 | 423.9 | 52.45% | 98.84% | 95.89% |
| GA5 | 107968 | 106789 | 104593 | 103885 | 43779194 | 421.42 | 50.04% | 98.76% | 95.65% |
| GA6 | 104833 | 103772 | 101844 | 97367 | 40779496 | 418.82 | 50.48% | 98.87% | 95.95% |
| GA7 | 105984 | 104908 | 102915 | 102388 | 42392632 | 414.04 | 50.70% | 98.78% | 95.64% |
| GA8 | 114883 | 113691 | 111515 | 99072 | 41652419 | 420.43 | 51.48% | 98.68% | 95.46% |
| GA9 | 180944 | 177559 | 171306 | 123468 | 52399428 | 424.4 | 52.08% | 97.86% | 93.25% |
| GA10 | 105242 | 104113 | 102074 | 88497 | 37164875 | 419.96 | 51.31% | 98.79% | 95.79% |
| GA11 | 82153 | 81399 | 79710 | 79129 | 32950090 | 416.41 | 50.60% | 98.81% | 95.74% |
| GA12 | 104688 | 103591 | 101617 | 97193 | 40455487 | 416.24 | 50.07% | 98.84% | 95.88% |
| GA13 | 103095 | 101567 | 99440 | 98762 | 41285970 | 418.03 | 51.49% | 98.75% | 95.65% |
| GA14 | 104386 | 103235 | 101146 | 96383 | 40606214 | 421.3 | 51.08% | 98.83% | 95.86% |
| GA15 | 106400 | 105259 | 103322 | 101220 | 42328699 | 418.19 | 51.41% | 98.83% | 95.87% |
| GA16 | 103170 | 102298 | 100256 | 95489 | 39468645 | 413.33 | 51.48% | 98.89% | 96.06% |
| GA17 | 103459 | 102407 | 100604 | 93420 | 38940227 | 416.83 | 52.09% | 98.94% | 96.19% |
| GA18 | 105383 | 104265 | 102201 | 100584 | 41822956 | 415.8 | 50.94% | 98.83% | 95.86% |
| GA19 | 113854 | 112645 | 111174 | 109146 | 46543470 | 426.43 | 53.28% | 98.88% | 95.96% |
| GA20 | 61296 | 60725 | 59410 | 57804 | 24378180 | 421.74 | 50.88% | 98.84% | 95.86% |
| GA21 | 102787 | 101697 | 99611 | 97113 | 40793515 | 420.06 | 51.43% | 98.88% | 96.03% |
| GA22 | 106724 | 105745 | 104386 | 71788 | 30103897 | 419.34 | 53.66% | 98.84% | 95.85% |
| GA23 | 110587 | 109375 | 107099 | 106718 | 44876084 | 420.51 | 52.96% | 98.79% | 95.73% |
| GA24 | 103815 | 102538 | 100433 | 99142 | 41642189 | 420.03 | 51.82% | 98.81% | 95.83% |
| GA25 | 104743 | 103581 | 101602 | 97430 | 40718647 | 417.93 | 50.59% | 98.89% | 96.01% |
| GA26 | 104946 | 103736 | 101648 | 95658 | 39932459 | 417.45 | 50.94% | 98.84% | 95.91% |
| GA27 | 106512 | 105106 | 102810 | 95594 | 40419585 | 422.83 | 51.68% | 98.66% | 95.24% |
| GA28 | 105278 | 104093 | 101941 | 100792 | 42169050 | 418.38 | 50.86% | 98.81% | 95.74% |
| GA29 | 104623 | 103678 | 101979 | 101174 | 41869083 | 413.83 | 52.07% | 98.94% | 96.15% |
| GA30 | 102003 | 100918 | 98734 | 96537 | 39948866 | 413.82 | 51.46% | 98.81% | 95.77% |
| GA31 | 106399 | 105243 | 103163 | 102842 | 43027759 | 418.39 | 51.52% | 98.85% | 95.90% |
| GA32 | 104445 | 103467 | 101529 | 100821 | 42170852 | 418.27 | 51.31% | 98.91% | 96.09% |
| GA33 | 106431 | 105336 | 103214 | 102319 | 43123567 | 421.46 | 51.54% | 98.76% | 95.67% |
| GA34 | 134106 | 132253 | 129067 | 125136 | 51500502 | 411.56 | 50.67% | 98.47% | 94.79% |
| GA35 | 105545 | 104411 | 102917 | 90938 | 38507226 | 423.44 | 51.94% | 98.83% | 95.79% |
| GA36 | 106548 | 105632 | 103682 | 103056 | 42742991 | 414.75 | 52.16% | 98.86% | 95.92% |
| GA37 | 119998 | 118499 | 116187 | 114470 | 48032792 | 419.61 | 51.76% | 98.85% | 95.90% |
| GA38 | 113297 | 112159 | 109833 | 104378 | 44472021 | 426.07 | 52.74% | 98.82% | 95.77% |
| GA39 | 104391 | 103228 | 101005 | 97393 | 41294623 | 424 | 51.82% | 98.75% | 95.58% |
| GA40 | 102316 | 101235 | 99051 | 94208 | 38636078 | 410.11 | 52.07% | 98.79% | 95.69% |
| GA41 | 112592 | 111295 | 109024 | 106632 | 44099053 | 413.56 | 51.77% | 98.84% | 95.83% |
| GA42 | 109601 | 108531 | 106384 | 104848 | 42989297 | 410.02 | 51.56% | 98.88% | 95.99% |
| GA43 | 105012 | 103867 | 101903 | 98932 | 41264648 | 417.1 | 51.08% | 98.84% | 95.86% |
| GA44 | 68887 | 68055 | 66478 | 64663 | 27099513 | 419.09 | 51.60% | 98.54% | 95.08% |
| GA45 | 105933 | 104849 | 102815 | 102526 | 42664082 | 416.13 | 50.21% | 98.87% | 95.93% |
| GA46 | 104265 | 103041 | 101082 | 100026 | 41881667 | 418.71 | 51.02% | 98.86% | 95.97% |
| GA47 | 104713 | 103353 | 101006 | 94264 | 39763857 | 421.84 | 50.90% | 98.66% | 95.41% |
| GA48 | 106462 | 105335 | 103284 | 97154 | 40392977 | 415.76 | 51.81% | 98.83% | 95.84% |
| GA49 | 102764 | 101521 | 99282 | 96426 | 40355081 | 418.51 | 50.07% | 98.62% | 95.24% |
| GA50 | 52918 | 52428 | 51432 | 50012 | 20906919 | 418.04 | 50.97% | 98.90% | 96.11% |
| GA51 | 67925 | 67245 | 65959 | 65523 | 27565616 | 420.7 | 51.56% | 98.81% | 95.83% |
| GA52 | 61921 | 61326 | 60152 | 56306 | 23455834 | 416.58 | 50.70% | 98.83% | 95.86% |
| GA53 | 98448 | 97329 | 95202 | 89363 | 37759490 | 422.54 | 50.09% | 98.76% | 95.66% |
| GA54 | 66102 | 65590 | 64378 | 63396 | 26267356 | 414.34 | 52.91% | 98.90% | 96.04% |
| GA55 | 111092 | 109921 | 107786 | 96086 | 40336299 | 419.79 | 51.49% | 98.87% | 95.97% |
| GA56 | 102533 | 101355 | 99286 | 94325 | 39401547 | 417.72 | 50.73% | 98.81% | 95.75% |
| GA57 | 106102 | 105232 | 103313 | 102404 | 42405029 | 414.1 | 52.34% | 98.90% | 96.07% |
| GA58 | 104254 | 103185 | 101725 | 62733 | 26195918 | 417.58 | 52.33% | 98.81% | 95.74% |
| GA59 | 107932 | 106626 | 104473 | 93466 | 39295348 | 420.42 | 49.51% | 98.78% | 95.65% |
| OB1 | 104584 | 103256 | 100924 | 98491 | 41566074 | 422.03 | 51.68% | 98.65% | 95.35% |
| OB2 | 113074 | 111974 | 109970 | 108443 | 45633673 | 420.81 | 51.22% | 98.86% | 95.88% |
| OB3 | 101627 | 100630 | 98552 | 96116 | 40499477 | 421.36 | 50.24% | 98.86% | 95.91% |
| OB4 | 102533 | 101266 | 99060 | 94608 | 40367851 | 426.69 | 52.87% | 98.68% | 95.33% |
| OB5 | 103568 | 102285 | 99661 | 90687 | 38567304 | 425.28 | 52.77% | 98.55% | 95.00% |
| OB6 | 105027 | 103759 | 101427 | 98097 | 41606215 | 424.13 | 51.89% | 98.80% | 95.78% |
| OB7 | 276749 | 271996 | 262961 | 141972 | 59188166 | 416.9 | 50.85% | 97.98% | 93.61% |
| OB8 | 86113 | 85118 | 83278 | 82073 | 34771316 | 423.66 | 51.26% | 98.81% | 95.83% |
| OB9 | 103592 | 102387 | 100230 | 96424 | 40791460 | 423.04 | 51.03% | 98.80% | 95.74% |
| OB10 | 96992 | 95863 | 93605 | 88926 | 37801055 | 425.08 | 52.56% | 98.72% | 95.55% |
| OB11 | 51659 | 51128 | 49998 | 46938 | 20005128 | 426.2 | 52.58% | 98.78% | 95.69% |
| OB12 | 116165 | 114858 | 112428 | 111998 | 47557656 | 424.63 | 52.14% | 98.80% | 95.80% |
| OB13 | 80375 | 79544 | 77653 | 77446 | 33074627 | 427.07 | 52.32% | 98.73% | 95.55% |
| OB14 | 106182 | 105019 | 102767 | 100268 | 41918281 | 418.06 | 52.72% | 98.83% | 95.83% |
| OB15 | 125549 | 123435 | 120228 | 116012 | 48829849 | 420.9 | 50.92% | 98.44% | 94.68% |
| OB16 | 104242 | 103273 | 101915 | 96649 | 40760441 | 421.74 | 53.94% | 98.87% | 95.90% |
| OB17 | 81110 | 80416 | 79429 | 70891 | 29927707 | 422.17 | 52.88% | 98.74% | 95.55% |
| OB18 | 102839 | 101723 | 100361 | 88486 | 37523708 | 424.06 | 52.23% | 98.60% | 95.09% |
| OB19 | 103276 | 102202 | 100723 | 85137 | 35589631 | 418.03 | 53.41% | 98.71% | 95.50% |
| OB20 | 65309 | 64747 | 63852 | 60069 | 25642115 | 426.88 | 52.09% | 98.76% | 95.57% |
| OB21 | 133622 | 131283 | 127601 | 126381 | 54054952 | 427.71 | 52.38% | 98.18% | 94.05% |
| OB22 | 111556 | 110315 | 108933 | 108081 | 45693042 | 422.77 | 52.65% | 98.59% | 95.15% |
| OB23 | 74895 | 74121 | 73168 | 68692 | 29145716 | 424.3 | 52.58% | 98.77% | 95.65% |
| OB24 | 103852 | 103037 | 101791 | 95627 | 40592917 | 424.49 | 53.70% | 98.85% | 95.87% |
| OB25 | 138969 | 137145 | 135429 | 127563 | 53565932 | 419.92 | 57.23% | 98.82% | 95.90% |
| OB26 | 77032 | 76321 | 75251 | 69306 | 29304773 | 422.83 | 52.15% | 98.75% | 95.54% |
| OB27 | 150680 | 149739 | 146950 | 139289 | 59068321 | 424.07 | 53.75% | 98.47% | 94.70% |
| OB28 | 45808 | 45232 | 44658 | 44431 | 18975962 | 427.09 | 52.09% | 98.66% | 95.41% |
| OB30 | 103453 | 102402 | 100955 | 98849 | 41968590 | 424.57 | 51.68% | 98.80% | 95.66% |
| OB31 | 79083 | 78430 | 77525 | 75715 | 31971944 | 422.27 | 53.31% | 98.85% | 95.91% |
| OB32 | 106444 | 105286 | 103925 | 100368 | 42513671 | 423.58 | 53.45% | 98.79% | 95.66% |
| OB33 | 98774 | 97920 | 95977 | 91372 | 38959708 | 426.39 | 51.93% | 98.61% | 95.18% |
| OB34 | 195420 | 193490 | 188957 | 178480 | 75955601 | 425.57 | 52.49% | 98.22% | 93.99% |
| OB35 | 79386 | 78648 | 77681 | 67875 | 28652465 | 422.14 | 52.70% | 98.90% | 95.99% |
| OB36 | 105456 | 104489 | 103182 | 81370 | 34411355 | 422.9 | 52.81% | 98.71% | 95.52% |
| OB37 | 55585 | 55178 | 54513 | 52947 | 22539274 | 425.7 | 52.96% | 98.86% | 95.90% |
| OB38 | 146592 | 144539 | 142319 | 132908 | 55795152 | 419.8 | 54.18% | 98.68% | 95.40% |
| OB39 | 103889 | 103040 | 101702 | 99969 | 42521026 | 425.34 | 52.30% | 98.82% | 95.74% |
| OB40 | 65313 | 64673 | 63755 | 62519 | 26316235 | 420.93 | 50.93% | 98.70% | 95.41% |
| OB41 | 102698 | 101681 | 100228 | 97011 | 41315772 | 425.89 | 52.18% | 98.65% | 95.29% |
| OB42 | 57768 | 57160 | 56450 | 55423 | 23398803 | 422.19 | 52.21% | 98.85% | 95.89% |
| OB43 | 104768 | 103554 | 102092 | 100241 | 42380540 | 422.79 | 53.21% | 98.59% | 95.12% |
| OB44 | 119463 | 118318 | 116727 | 114958 | 48942523 | 425.74 | 51.87% | 98.64% | 95.28% |
| OB45 | 104218 | 103111 | 101681 | 95978 | 40757410 | 424.65 | 51.15% | 98.63% | 95.22% |
| OB46 | 57056 | 56332 | 55585 | 55346 | 23579477 | 426.04 | 52.33% | 98.73% | 95.52% |
| OB47 | 104214 | 103075 | 101666 | 98981 | 42029779 | 424.62 | 52.51% | 98.70% | 95.40% |
| OB48 | 105431 | 104191 | 102674 | 95551 | 40554743 | 424.43 | 52.12% | 98.70% | 95.49% |
| OB49 | 112223 | 110987 | 109465 | 99926 | 42210144 | 422.41 | 51.39% | 98.81% | 95.69% |
| OB50 | 71495 | 70946 | 70187 | 68419 | 29087324 | 425.14 | 52.54% | 98.93% | 96.07% |
| OB51 | 56187 | 55740 | 55027 | 51103 | 21631499 | 423.29 | 53.61% | 98.76% | 95.63% |
| OB52 | 104574 | 103620 | 102341 | 101079 | 42981503 | 425.23 | 52.66% | 98.72% | 95.46% |
| OB53 | 105136 | 103965 | 102555 | 96197 | 40724311 | 423.34 | 52.63% | 98.68% | 95.34% |
| OB54 | 157985 | 156947 | 154042 | 153207 | 65131250 | 425.12 | 53.98% | 98.42% | 94.63% |
| OB55 | 137078 | 135207 | 131864 | 128256 | 54649059 | 426.09 | 51.97% | 98.32% | 94.41% |
| OB56 | 73404 | 72762 | 71814 | 69628 | 29577145 | 424.79 | 52.24% | 98.79% | 95.66% |
| OB57 | 56646 | 56184 | 55374 | 55081 | 23006257 | 417.68 | 52.47% | 98.74% | 95.59% |
| OB58 | 70901 | 70281 | 69428 | 67073 | 28336778 | 422.48 | 51.47% | 98.78% | 95.66% |
| OB59 | 104094 | 102959 | 101547 | 96284 | 40806102 | 423.81 | 51.63% | 98.72% | 95.48% |
| VB1 | 122812 | 120680 | 116921 | 108901 | 46694675 | 428.78 | 50.92% | 98.09% | 93.74% |
| VB2 | 109324 | 108493 | 106953 | 105002 | 44321093 | 422.1 | 53.63% | 98.79% | 95.61% |
| VB3 | 104803 | 103972 | 102274 | 101261 | 43440202 | 428.99 | 51.06% | 98.55% | 94.83% |
| VB4 | 147749 | 146401 | 143655 | 142603 | 61166935 | 428.93 | 50.27% | 98.45% | 94.67% |
| VB5 | 115802 | 114998 | 113482 | 111888 | 47989310 | 428.9 | 50.91% | 98.81% | 95.71% |
| VB6 | 110313 | 109464 | 107790 | 107282 | 46020088 | 428.96 | 51.51% | 98.83% | 95.80% |
| VB7 | 93192 | 91468 | 87959 | 46031 | 19194771 | 417 | 51.39% | 97.91% | 93.46% |
| VB8 | 107181 | 106240 | 104454 | 103038 | 43956414 | 426.6 | 51.35% | 98.68% | 95.31% |
| VB9 | 102073 | 101251 | 100103 | 100021 | 41067237 | 410.59 | 58.27% | 98.97% | 96.41% |
| VB10 | 117184 | 116202 | 114444 | 111510 | 47074628 | 422.16 | 52.91% | 98.67% | 95.37% |
| VB11 | 216152 | 213574 | 207946 | 200332 | 85659902 | 427.59 | 51.92% | 97.90% | 93.25% |
| VB12 | 90120 | 89487 | 88295 | 88127 | 37806218 | 429 | 51.49% | 98.92% | 96.12% |
| VB13 | 103862 | 102971 | 101744 | 101398 | 41851895 | 412.75 | 57.49% | 98.90% | 96.16% |
| VB14 | 111706 | 110415 | 108757 | 107541 | 46096060 | 428.64 | 49.95% | 98.69% | 95.26% |
| VB15 | 103096 | 102034 | 100712 | 99137 | 41312910 | 416.73 | 52.71% | 98.85% | 95.85% |
| VB16 | 103808 | 102917 | 101243 | 97604 | 41859781 | 428.87 | 51.28% | 98.78% | 95.65% |
| VB17 | 97772 | 97054 | 95548 | 95124 | 40796684 | 428.88 | 51.08% | 98.86% | 95.78% |
| VB18 | 134379 | 132627 | 128961 | 128514 | 55121837 | 428.92 | 51.09% | 98.30% | 94.28% |
| VB19 | 114311 | 113180 | 111344 | 111173 | 47686177 | 428.94 | 51.05% | 98.78% | 95.57% |
| VB20 | 86888 | 86262 | 85058 | 85048 | 36484807 | 428.99 | 51.46% | 98.91% | 96.09% |
| VB21 | 81217 | 80012 | 78620 | 78453 | 33642652 | 428.83 | 51.73% | 98.44% | 94.59% |
| VB22 | 86972 | 86337 | 85041 | 84894 | 36406775 | 428.85 | 51.09% | 98.91% | 95.99% |
| VB23 | 103854 | 102669 | 100966 | 98473 | 42227309 | 428.82 | 51.94% | 98.77% | 95.60% |
| VB24 | 143917 | 142895 | 140403 | 139447 | 59821042 | 428.99 | 51.55% | 98.72% | 95.46% |
| VB25 | 115492 | 114615 | 112991 | 111029 | 47630051 | 428.99 | 51.43% | 98.90% | 96.04% |
| VB26 | 75388 | 74789 | 73669 | 73036 | 31331342 | 428.98 | 51.40% | 98.70% | 95.48% |
| VB27 | 63543 | 63037 | 62207 | 62200 | 26681906 | 428.97 | 51.52% | 98.91% | 96.10% |
| VB29 | 125724 | 123866 | 120300 | 120287 | 51599360 | 428.97 | 51.40% | 98.27% | 94.31% |
| VB30 | 110355 | 109449 | 107680 | 107654 | 46182160 | 428.99 | 51.53% | 98.78% | 95.68% |
| VB31 | 148468 | 147387 | 144987 | 144940 | 62176540 | 428.98 | 51.53% | 98.74% | 95.52% |
| VB32 | 105380 | 104607 | 103178 | 103123 | 44235869 | 428.96 | 51.52% | 98.94% | 96.12% |
| VB33 | 104573 | 103583 | 101715 | 101499 | 43540535 | 428.98 | 51.06% | 98.74% | 95.47% |
| VB34 | 102303 | 101539 | 100083 | 99986 | 42891690 | 428.98 | 51.51% | 98.90% | 96.05% |
| VB35 | 153534 | 152102 | 148933 | 127857 | 54848365 | 428.98 | 51.38% | 98.32% | 94.32% |
| VB36 | 88474 | 87824 | 86627 | 86618 | 37155840 | 428.96 | 51.52% | 98.95% | 96.19% |
| VB37 | 52498 | 52074 | 51468 | 51389 | 21132254 | 411.22 | 56.94% | 98.83% | 95.93% |
| VB38 | 106036 | 104932 | 103556 | 101524 | 42827150 | 421.84 | 52.85% | 98.79% | 95.72% |
| VB39 | 74557 | 74055 | 73042 | 73040 | 31333322 | 428.99 | 51.52% | 98.95% | 96.19% |
| VB40 | 102384 | 101563 | 100018 | 99955 | 42875404 | 428.95 | 51.51% | 98.92% | 96.05% |
| VB41 | 65909 | 65385 | 64317 | 64285 | 27572954 | 428.92 | 51.54% | 98.75% | 95.52% |
| VB42 | 106362 | 105512 | 103865 | 103666 | 44471249 | 428.99 | 51.02% | 98.86% | 95.87% |
| VB43 | 54205 | 53839 | 53029 | 52984 | 22719839 | 428.81 | 51.55% | 98.96% | 96.22% |
| VB44 | 61374 | 60904 | 59937 | 59406 | 25483569 | 428.97 | 51.09% | 98.90% | 95.94% |
| VB45 | 63817 | 63352 | 62344 | 61603 | 26425490 | 428.96 | 50.59% | 98.89% | 95.94% |
| VB46 | 142247 | 134924 | 131492 | 130696 | 54604227 | 417.8 | 52.36% | 98.23% | 94.13% |
| VB47 | 50515 | 50012 | 49345 | 49273 | 21132114 | 428.88 | 51.97% | 98.61% | 95.23% |
| VB48 | 103875 | 103129 | 101532 | 101206 | 43330157 | 428.14 | 50.88% | 98.89% | 95.94% |
| VB49 | 112387 | 111382 | 109528 | 108600 | 46397821 | 427.24 | 50.54% | 98.75% | 95.55% |
| VB50 | 63113 | 62558 | 61567 | 61433 | 26349380 | 428.91 | 51.10% | 98.84% | 95.81% |
| VB51 | 105007 | 104170 | 102463 | 97061 | 41638375 | 428.99 | 51.32% | 98.81% | 95.76% |
| VB52 | 104349 | 103420 | 100792 | 96286 | 41306157 | 428.99 | 51.35% | 98.76% | 95.68% |
| VB53 | 99321 | 98482 | 96121 | 95088 | 40789737 | 428.97 | 51.07% | 98.78% | 95.66% |
| VB54 | 103679 | 102701 | 100236 | 99944 | 42873742 | 428.98 | 51.75% | 98.72% | 95.59% |
| VB55 | 105447 | 104098 | 101506 | 99810 | 42363225 | 424.44 | 52.96% | 98.73% | 95.60% |
| VB56 | 89633 | 88866 | 86569 | 86536 | 37122998 | 428.99 | 51.52% | 98.75% | 95.67% |
| VB57 | 103265 | 102473 | 100975 | 98389 | 42151122 | 428.41 | 51.25% | 98.86% | 95.85% |
| VB58 | 103470 | 102592 | 100916 | 97056 | 41634839 | 428.98 | 51.76% | 98.61% | 95.26% |
| VB59 | 106541 | 105735 | 103643 | 103224 | 42784981 | 414.49 | 56.73% | 98.86% | 95.83% |
| GB1 | 105001 | 103609 | 101303 | 97127 | 40728966 | 419.34 | 50.17% | 98.78% | 95.72% |
| GB2 | 113261 | 111808 | 109481 | 97845 | 40784213 | 416.82 | 50.67% | 98.82% | 95.82% |
| GB3 | 105030 | 103538 | 101482 | 88916 | 37011900 | 416.26 | 51.99% | 98.82% | 95.86% |
| GB4 | 103893 | 102338 | 99976 | 88901 | 37602819 | 422.97 | 52.74% | 98.64% | 95.27% |
| GB5 | 103570 | 102298 | 99857 | 93881 | 39620480 | 422.03 | 52.22% | 98.70% | 95.50% |
| GB6 | 102483 | 101765 | 99597 | 80563 | 33404363 | 414.64 | 51.86% | 98.66% | 95.34% |
| GB7 | 98162 | 96487 | 93021 | 45002 | 18730411 | 416.21 | 50.97% | 98.09% | 93.89% |
| GB8 | 104860 | 103596 | 101586 | 79095 | 33099054 | 418.47 | 51.46% | 98.64% | 95.37% |
| GB9 | 102775 | 101665 | 99452 | 89900 | 37712322 | 419.49 | 51.74% | 98.76% | 95.71% |
| GB10 | 101685 | 100645 | 98659 | 84028 | 34869634 | 414.98 | 51.09% | 98.88% | 96.02% |
| GB11 | 115254 | 113542 | 111021 | 107535 | 45070093 | 419.12 | 51.07% | 98.58% | 95.12% |
| GB12 | 97905 | 96902 | 95127 | 92355 | 38326620 | 414.99 | 50.05% | 98.85% | 95.90% |
| GB13 | 104104 | 103040 | 100846 | 97404 | 41023273 | 421.17 | 52.10% | 98.80% | 95.80% |
| GB14 | 96077 | 95176 | 93246 | 85303 | 35793268 | 419.6 | 51.64% | 98.88% | 95.99% |
| GB15 | 55403 | 54806 | 53600 | 45736 | 19058955 | 416.72 | 49.84% | 98.84% | 95.91% |
| GB16 | 68236 | 67635 | 66227 | 59900 | 24650580 | 411.53 | 51.76% | 98.89% | 96.02% |
| GB17 | 154070 | 152654 | 149727 | 131785 | 54726191 | 415.27 | 51.87% | 98.77% | 95.73% |
| GB18 | 84420 | 83598 | 81858 | 67410 | 28023492 | 415.72 | 52.36% | 98.76% | 95.67% |
| GB19 | 106128 | 104872 | 102464 | 99149 | 40821305 | 411.72 | 53.01% | 98.75% | 95.62% |
| GB20 | 82285 | 81512 | 79809 | 76180 | 31950365 | 419.41 | 50.85% | 98.86% | 95.91% |
| GB21 | 135571 | 133586 | 130487 | 121276 | 51027672 | 420.76 | 51.68% | 98.55% | 95.07% |
| GB22 | 136740 | 134907 | 131714 | 127030 | 52878497 | 416.27 | 52.61% | 98.50% | 94.89% |
| GB23 | 108888 | 107286 | 104263 | 100730 | 43061791 | 427.5 | 51.62% | 98.28% | 94.18% |
| GB24 | 135490 | 133105 | 129749 | 127162 | 53179259 | 418.2 | 51.88% | 98.39% | 94.71% |
| GB25 | 115715 | 114700 | 112752 | 99999 | 41247440 | 412.48 | 51.74% | 98.82% | 95.72% |
| GB26 | 75535 | 74791 | 73235 | 64876 | 27169378 | 418.79 | 50.70% | 98.86% | 95.97% |
| GB27 | 103984 | 102978 | 101022 | 93030 | 38313801 | 411.84 | 51.42% | 98.87% | 96.01% |
| GB28 | 95700 | 94668 | 92542 | 85215 | 35559678 | 417.29 | 50.72% | 98.83% | 95.86% |
| GB29 | 56168 | 55626 | 54459 | 53455 | 22218987 | 415.66 | 52.29% | 98.89% | 96.07% |
| GB30 | 126296 | 124086 | 120866 | 111707 | 46140549 | 413.05 | 50.72% | 98.37% | 94.50% |
| GB31 | 56910 | 56266 | 55092 | 47937 | 20054263 | 418.35 | 51.18% | 98.82% | 95.84% |
| GB32 | 81396 | 80682 | 79157 | 74527 | 31538989 | 423.19 | 51.24% | 98.92% | 96.10% |
| GB33 | 103129 | 101741 | 99493 | 88761 | 37172975 | 418.8 | 51.29% | 98.70% | 95.43% |
| GB34 | 106618 | 105391 | 103206 | 100082 | 41748715 | 417.15 | 49.78% | 98.78% | 95.71% |
| GB35 | 103888 | 102783 | 100837 | 96220 | 39910170 | 414.78 | 49.95% | 98.90% | 96.00% |
| GB36 | 102621 | 101539 | 99315 | 95664 | 39612971 | 414.08 | 50.92% | 98.78% | 95.67% |
| GB37 | 103494 | 102378 | 100256 | 89428 | 37401847 | 418.23 | 51.16% | 98.80% | 95.74% |
| GB38 | 105435 | 104115 | 101666 | 86401 | 36442204 | 421.78 | 51.72% | 98.75% | 95.63% |
| GB39 | 103468 | 102317 | 99995 | 90985 | 38086647 | 418.6 | 50.57% | 98.80% | 95.70% |
| GB40 | 117666 | 116533 | 114291 | 99749 | 41966214 | 420.72 | 51.92% | 98.80% | 95.79% |
| GB41 | 103553 | 102396 | 100159 | 84740 | 35192978 | 415.31 | 50.88% | 98.81% | 95.80% |
| GB42 | 102018 | 100990 | 99082 | 93416 | 38687544 | 414.14 | 51.71% | 98.89% | 96.04% |
| GB43 | 102810 | 101562 | 99391 | 86890 | 36355604 | 418.41 | 50.55% | 98.75% | 95.57% |
| GB44 | 105998 | 104523 | 102256 | 96676 | 40760005 | 421.61 | 51.16% | 98.72% | 95.50% |
| GB45 | 77723 | 76860 | 75208 | 69932 | 29068658 | 415.67 | 49.90% | 98.82% | 95.86% |
| GB46 | 106308 | 105121 | 102738 | 79426 | 32958624 | 414.96 | 51.82% | 98.81% | 95.81% |
| GB47 | 114134 | 112941 | 110455 | 108716 | 45648833 | 419.89 | 50.99% | 98.80% | 95.79% |
| GB48 | 103044 | 101759 | 99657 | 91835 | 38355938 | 417.66 | 51.21% | 98.78% | 95.73% |
| GB49 | 102269 | 100982 | 98733 | 94714 | 39794397 | 420.15 | 50.39% | 98.76% | 95.67% |
| GB50 | 104738 | 103540 | 101376 | 88777 | 37233345 | 419.4 | 50.72% | 98.85% | 95.92% |
| GB51 | 102695 | 101369 | 99009 | 93334 | 39247446 | 420.51 | 51.59% | 98.71% | 95.48% |
| GB52 | 102846 | 101603 | 99030 | 93341 | 39665346 | 424.95 | 51.15% | 98.60% | 95.09% |
| GB53 | 113028 | 111572 | 109086 | 97006 | 40831586 | 420.92 | 50.03% | 98.73% | 95.56% |
| GB54 | 113600 | 112542 | 110369 | 103189 | 42705838 | 413.86 | 51.85% | 98.85% | 95.90% |
| GB55 | 119084 | 117628 | 115031 | 96057 | 40176040 | 418.25 | 51.32% | 98.80% | 95.78% |
| GB56 | 104725 | 103367 | 101075 | 97685 | 40870293 | 418.39 | 50.96% | 98.72% | 95.51% |
| GB57 | 104686 | 103539 | 101484 | 98400 | 40972607 | 416.39 | 50.64% | 98.83% | 95.85% |
| GB58 | 104724 | 103538 | 101357 | 94168 | 39194628 | 416.22 | 51.81% | 98.78% | 95.73% |
| GB59 | 90664 | 89608 | 87755 | 78975 | 33272520 | 421.3 | 50.01% | 98.81% | 95.72% |
| OC1 | 104898 | 103709 | 101366 | 100353 | 42590738 | 424.41 | 52.08% | 98.72% | 95.51% |
| OC2 | 104091 | 102677 | 100169 | 99207 | 42389683 | 427.29 | 51.55% | 98.50% | 94.91% |
| OC3 | 114215 | 112814 | 110199 | 107065 | 44874675 | 419.13 | 49.92% | 98.74% | 95.53% |
| OC4 | 102510 | 101426 | 99121 | 90455 | 38581653 | 426.53 | 52.80% | 98.64% | 95.29% |
| OC5 | 103141 | 101771 | 99618 | 90049 | 38144523 | 423.6 | 51.41% | 98.72% | 95.45% |
| OC6 | 103213 | 101696 | 99234 | 91976 | 39137190 | 425.52 | 52.77% | 98.64% | 95.25% |
| OC7 | 107312 | 106145 | 103776 | 101972 | 43539222 | 426.97 | 51.88% | 98.77% | 95.61% |
| OC8 | 105571 | 103972 | 101588 | 93102 | 39407478 | 423.27 | 52.08% | 98.63% | 95.29% |
| OC9 | 102327 | 101150 | 99162 | 95020 | 39804168 | 418.9 | 51.32% | 98.78% | 95.64% |
| OC10 | 95334 | 94109 | 91760 | 84239 | 35660878 | 423.33 | 51.75% | 98.57% | 95.10% |
| OC11 | 105793 | 104452 | 102063 | 99169 | 42291117 | 426.46 | 51.90% | 98.69% | 95.41% |
| OC12 | 106425 | 104744 | 102408 | 93225 | 39503860 | 423.75 | 52.13% | 98.71% | 95.52% |
| OC13 | 92935 | 91510 | 88805 | 42387 | 17637001 | 416.09 | 51.08% | 98.22% | 94.19% |
| OC14 | 103202 | 101944 | 99471 | 93587 | 39862304 | 425.94 | 52.04% | 98.69% | 95.45% |
| OC15 | 102213 | 100359 | 98251 | 95569 | 40435844 | 423.11 | 51.84% | 98.72% | 95.53% |
| OC16 | 103127 | 101500 | 98875 | 98687 | 42205764 | 427.67 | 51.73% | 98.36% | 94.55% |
| OC17 | 103392 | 102227 | 100038 | 92134 | 39131670 | 424.73 | 51.73% | 98.76% | 95.70% |
| OC18 | 106298 | 105034 | 102888 | 98053 | 41728014 | 425.57 | 51.74% | 98.78% | 95.67% |
| OC19 | 115128 | 113524 | 110887 | 77849 | 32659336 | 419.52 | 52.55% | 98.70% | 95.50% |
| OC20 | 102219 | 100892 | 98537 | 95083 | 40217149 | 422.97 | 51.25% | 98.71% | 95.48% |
| OC21 | 104942 | 103734 | 101524 | 96942 | 41286471 | 425.89 | 52.22% | 98.74% | 95.58% |
| OC22 | 105985 | 104576 | 102319 | 98154 | 41625322 | 424.08 | 52.78% | 98.67% | 95.37% |
| OC23 | 89415 | 88016 | 85216 | 42839 | 17869959 | 417.14 | 50.95% | 98.19% | 94.15% |
| OC24 | 118014 | 116527 | 113650 | 112205 | 47805711 | 426.06 | 51.76% | 98.57% | 95.09% |
| OC25 | 102079 | 100822 | 98607 | 95413 | 40578135 | 425.29 | 52.75% | 98.60% | 95.23% |
| OC26 | 112484 | 110960 | 108489 | 96597 | 40659695 | 420.92 | 56.79% | 98.73% | 95.57% |
| OC27 | 103076 | 102147 | 100104 | 98765 | 42100628 | 426.27 | 53.49% | 98.87% | 95.95% |
| OC29 | 102693 | 101523 | 99646 | 98545 | 41519661 | 421.33 | 53.30% | 98.81% | 95.74% |
| OC30 | 116828 | 115712 | 113235 | 112728 | 48053410 | 426.28 | 52.63% | 98.70% | 95.46% |
| OC31 | 106456 | 104833 | 102533 | 98953 | 41732504 | 421.74 | 52.91% | 98.67% | 95.34% |
| OC32 | 102427 | 100857 | 98578 | 86009 | 36419459 | 423.44 | 51.62% | 98.58% | 95.06% |
| OC33 | 105317 | 104068 | 101875 | 97662 | 41388711 | 423.8 | 53.02% | 98.78% | 95.67% |
| OC34 | 113788 | 112623 | 110215 | 108687 | 46116260 | 424.3 | 51.71% | 98.74% | 95.55% |
| OC35 | 103655 | 102233 | 100100 | 89532 | 37773574 | 421.9 | 52.44% | 98.72% | 95.51% |
| OC36 | 104711 | 103424 | 101062 | 72137 | 30580414 | 423.92 | 52.11% | 98.59% | 95.20% |
| OC37 | 102055 | 100913 | 98701 | 93619 | 39727982 | 424.36 | 52.07% | 98.67% | 95.40% |
| OC38 | 106614 | 105370 | 103222 | 96776 | 40906672 | 422.69 | 54.81% | 98.71% | 95.52% |
| OC39 | 105287 | 104166 | 102010 | 96823 | 41184236 | 425.36 | 51.87% | 98.78% | 95.65% |
| OC40 | 107703 | 106402 | 104114 | 97283 | 41278837 | 424.32 | 51.31% | 98.68% | 95.39% |
| OC41 | 75845 | 75212 | 73609 | 67052 | 28420747 | 423.86 | 52.13% | 98.77% | 95.64% |
| OC42 | 102506 | 101170 | 98989 | 92299 | 39287207 | 425.65 | 52.71% | 98.64% | 95.33% |
| OC43 | 102688 | 101457 | 99748 | 71374 | 30313999 | 424.72 | 52.81% | 98.41% | 94.70% |
| OC44 | 76855 | 76124 | 75017 | 58464 | 24797126 | 424.14 | 51.89% | 98.56% | 95.00% |
| OC45 | 105797 | 104776 | 103288 | 76295 | 31695773 | 415.44 | 54.17% | 98.72% | 95.46% |
| OC46 | 135046 | 134069 | 131315 | 128635 | 54251113 | 421.74 | 54.45% | 98.79% | 95.69% |
| OC47 | 102328 | 101349 | 98732 | 77117 | 32826775 | 425.67 | 52.13% | 98.45% | 94.65% |
| OC48 | 103657 | 102508 | 100881 | 55530 | 23535352 | 423.83 | 52.45% | 98.50% | 94.73% |
| OC49 | 103768 | 102788 | 101246 | 65437 | 27742750 | 423.96 | 51.63% | 98.72% | 95.40% |
| OC50 | 56126 | 55644 | 54799 | 38779 | 16596461 | 427.98 | 52.28% | 98.64% | 95.17% |
| OC51 | 106239 | 105033 | 103139 | 68838 | 29210174 | 424.33 | 54.21% | 98.34% | 94.39% |
| OC52 | 66927 | 66076 | 64993 | 60290 | 25506462 | 423.06 | 53.18% | 98.36% | 94.38% |
| OC53 | 86496 | 85355 | 83483 | 57350 | 24225701 | 422.42 | 52.77% | 98.30% | 94.36% |
| OC54 | 102702 | 101801 | 100378 | 95149 | 40339177 | 423.96 | 54.80% | 98.68% | 95.23% |
| OC55 | 253040 | 248861 | 240394 | 149962 | 62516493 | 416.88 | 50.98% | 97.98% | 93.57% |
| OC56 | 119118 | 117999 | 116360 | 91385 | 38724387 | 423.75 | 51.84% | 98.72% | 95.44% |
| OC57 | 102697 | 101832 | 100627 | 99967 | 42445951 | 424.6 | 52.81% | 98.80% | 95.73% |
| OC58 | 102962 | 102102 | 100958 | 75472 | 32082953 | 425.1 | 52.49% | 98.80% | 95.70% |
| OC59 | 104935 | 103841 | 102586 | 89293 | 37623691 | 421.35 | 53.42% | 98.71% | 95.48% |
| VC1 | 52742 | 52315 | 51552 | 43068 | 18471161 | 428.88 | 51.12% | 98.81% | 95.63% |
| VC2 | 84562 | 84042 | 83162 | 80529 | 33492943 | 415.91 | 55.53% | 98.91% | 96.02% |
| VC3 | 105233 | 104526 | 102980 | 102778 | 44078900 | 428.87 | 51.03% | 98.75% | 95.49% |
| VC4 | 72408 | 71939 | 71091 | 63756 | 26985406 | 423.26 | 52.61% | 98.91% | 96.07% |
| VC5 | 69084 | 68622 | 67785 | 64813 | 27397711 | 422.72 | 53.03% | 98.88% | 95.91% |
| VC6 | 112383 | 111385 | 109892 | 106395 | 45565816 | 428.27 | 51.61% | 98.86% | 95.89% |
| VC7 | 116608 | 115467 | 112493 | 108513 | 46383251 | 427.44 | 51.55% | 98.73% | 95.52% |
| VC8 | 80594 | 79916 | 78037 | 74895 | 31706343 | 423.34 | 52.59% | 98.75% | 95.53% |
| VC9 | 103619 | 102480 | 100374 | 96332 | 39610602 | 411.19 | 57.75% | 98.83% | 96.02% |
| VC10 | 54196 | 53757 | 52598 | 50404 | 21163575 | 419.88 | 53.56% | 98.73% | 95.55% |
| VC11 | 95299 | 93788 | 91199 | 90355 | 38753979 | 428.91 | 51.77% | 98.14% | 93.77% |
| VC12 | 102840 | 101823 | 99248 | 91224 | 39132364 | 428.97 | 51.37% | 98.72% | 95.51% |
| VC13 | 98299 | 96764 | 94521 | 89708 | 37470796 | 417.7 | 55.22% | 98.71% | 95.59% |
| VC14 | 103214 | 101931 | 100409 | 88297 | 37795973 | 428.06 | 51.33% | 98.65% | 95.15% |
| VC15 | 106548 | 105454 | 104135 | 92217 | 38965700 | 422.54 | 55.08% | 98.78% | 95.71% |
| VC16 | 136828 | 134486 | 130040 | 110638 | 47454934 | 428.92 | 51.31% | 98.07% | 93.79% |
| VC17 | 103225 | 102319 | 100682 | 96462 | 41380959 | 428.99 | 51.06% | 98.57% | 94.99% |
| VC18 | 106338 | 105456 | 103768 | 102650 | 44029886 | 428.93 | 50.90% | 98.65% | 95.24% |
| VC19 | 113411 | 112577 | 111004 | 109082 | 46775572 | 428.81 | 51.05% | 98.82% | 95.73% |
| VC20 | 103693 | 102995 | 101523 | 101476 | 43531138 | 428.98 | 51.45% | 98.93% | 96.11% |
| VC21 | 99748 | 98758 | 97317 | 95334 | 40845574 | 428.45 | 52.13% | 98.77% | 95.53% |
| VC22 | 99929 | 99355 | 98122 | 95758 | 40909965 | 427.22 | 51.56% | 98.94% | 96.06% |
| VC23 | 102141 | 101262 | 99779 | 92120 | 39506316 | 428.86 | 51.61% | 98.60% | 95.10% |
| VC24 | 113296 | 112461 | 110816 | 107070 | 45928606 | 428.96 | 51.53% | 98.89% | 95.93% |
| VC25 | 105670 | 104881 | 103300 | 89462 | 38372968 | 428.93 | 51.28% | 98.84% | 95.82% |
| VC26 | 100304 | 99705 | 98398 | 97785 | 41936473 | 428.86 | 51.45% | 98.97% | 96.21% |
| VC27 | 117918 | 117239 | 115733 | 115551 | 49561851 | 428.92 | 51.52% | 98.91% | 96.00% |
| VC29 | 79323 | 78761 | 77637 | 77448 | 33214030 | 428.86 | 51.37% | 98.91% | 96.04% |
| VC30 | 106363 | 105709 | 104423 | 99343 | 42378521 | 426.59 | 52.22% | 98.96% | 96.18% |
| VC31 | 104657 | 103799 | 102305 | 102232 | 43854378 | 428.97 | 51.52% | 98.81% | 95.71% |
| VC32 | 106717 | 105907 | 104494 | 103825 | 44528487 | 428.88 | 51.54% | 98.82% | 95.78% |
| VC33 | 105255 | 104257 | 102666 | 97829 | 41824262 | 427.52 | 51.23% | 98.74% | 95.47% |
| VC34 | 104336 | 103599 | 102089 | 101758 | 43643327 | 428.89 | 51.48% | 98.85% | 95.87% |
| VC35 | 89111 | 88386 | 86477 | 77592 | 33284900 | 428.97 | 51.35% | 98.48% | 94.82% |
| VC36 | 55475 | 55109 | 54307 | 54228 | 23261200 | 428.95 | 51.52% | 98.96% | 96.14% |
| VC37 | 104148 | 103361 | 102180 | 94665 | 39205719 | 414.15 | 56.53% | 98.74% | 95.63% |
| VC38 | 113024 | 111729 | 109414 | 101285 | 42939017 | 423.94 | 52.87% | 98.45% | 94.63% |
| VC39 | 105547 | 104294 | 101695 | 100754 | 43209903 | 428.87 | 51.55% | 98.67% | 95.36% |
| VC40 | 105707 | 104565 | 102315 | 101883 | 43700776 | 428.93 | 51.52% | 98.80% | 95.74% |
| VC41 | 102830 | 101867 | 99402 | 97634 | 41859049 | 428.73 | 51.58% | 98.80% | 95.77% |
| VC42 | 104306 | 103228 | 100304 | 95372 | 40870188 | 428.53 | 51.13% | 98.64% | 95.14% |
| VC43 | 105361 | 104296 | 101686 | 94275 | 40428786 | 428.84 | 51.85% | 98.78% | 95.66% |
| VC44 | 105265 | 104057 | 101266 | 90011 | 38607324 | 428.92 | 51.20% | 98.73% | 95.52% |
| VC45 | 105386 | 104388 | 101988 | 100032 | 42911473 | 428.98 | 50.58% | 98.83% | 95.75% |
| VC46 | 102722 | 101283 | 98855 | 80553 | 34556023 | 428.98 | 51.58% | 98.58% | 95.08% |
| VC47 | 105261 | 103290 | 100417 | 94550 | 40558342 | 428.96 | 52.12% | 98.29% | 94.32% |
| VC48 | 105330 | 104487 | 102101 | 94756 | 39925599 | 421.35 | 53.25% | 98.80% | 95.71% |
| VC49 | 104075 | 103137 | 100831 | 87717 | 37507251 | 427.59 | 50.73% | 98.72% | 95.51% |
| VC50 | 102743 | 101789 | 99324 | 91443 | 39215586 | 428.85 | 51.14% | 98.77% | 95.62% |
| VC51 | 102219 | 101144 | 98724 | 84452 | 36220033 | 428.88 | 51.17% | 98.69% | 95.37% |
| VC52 | 72986 | 72402 | 70543 | 64482 | 27661219 | 428.98 | 51.36% | 98.78% | 95.74% |
| VC53 | 109481 | 108375 | 105500 | 103971 | 44603202 | 429 | 51.05% | 98.75% | 95.52% |
| VC54 | 104689 | 103809 | 102337 | 102295 | 43881061 | 428.97 | 51.76% | 98.76% | 95.62% |
| VC55 | 294060 | 290291 | 279622 | 256808 | 105442650 | 410.59 | 57.92% | 98.11% | 94.11% |
| VC56 | 104599 | 103757 | 102021 | 101979 | 43747053 | 428.98 | 51.52% | 98.78% | 95.64% |
| VC57 | 104619 | 103850 | 102295 | 102115 | 43805740 | 428.98 | 51.16% | 98.82% | 95.70% |
| VC58 | 104474 | 103068 | 99573 | 97209 | 41701256 | 428.99 | 51.73% | 98.09% | 93.66% |
| VC59 | 111643 | 110699 | 108434 | 93663 | 38484354 | 410.88 | 57.15% | 98.79% | 95.68% |
| GC1 | 103374 | 102119 | 100708 | 75170 | 31628226 | 420.76 | 51.11% | 98.67% | 95.31% |
| GC2 | 104774 | 103768 | 102532 | 60038 | 25019191 | 416.72 | 52.20% | 98.85% | 95.90% |
| GC3 | 103118 | 101978 | 100676 | 64886 | 27359764 | 421.66 | 52.06% | 98.78% | 95.70% |
| GC4 | 102608 | 101651 | 100370 | 62951 | 26514724 | 421.2 | 53.11% | 98.84% | 95.85% |
| GC5 | 102576 | 101503 | 100296 | 72029 | 29957717 | 415.91 | 51.49% | 98.86% | 95.90% |
| GC6 | 104155 | 103182 | 101873 | 100776 | 41873322 | 415.51 | 50.78% | 98.87% | 95.92% |
| GC7 | 105756 | 104598 | 103302 | 73951 | 30751794 | 415.84 | 50.97% | 98.74% | 95.50% |
| GC8 | 106351 | 104948 | 103554 | 67478 | 28338224 | 419.96 | 50.96% | 98.44% | 94.87% |
| GC9 | 103218 | 101898 | 100417 | 70783 | 29912972 | 422.6 | 52.16% | 98.51% | 94.80% |
| GC10 | 106155 | 105179 | 103971 | 80572 | 33105470 | 410.88 | 49.60% | 98.87% | 95.88% |
| GC11 | 103611 | 102688 | 101547 | 61610 | 25548806 | 414.69 | 50.45% | 98.92% | 96.02% |
| GC12 | 104003 | 102906 | 101464 | 69133 | 29269051 | 423.37 | 52.06% | 98.78% | 95.64% |
| GC13 | 106079 | 104764 | 103352 | 69614 | 29379024 | 422.03 | 52.09% | 98.60% | 95.20% |
| GC14 | 102810 | 101734 | 100450 | 63981 | 26926301 | 420.85 | 50.80% | 98.81% | 95.75% |
| GC15 | 102330 | 101133 | 99855 | 82806 | 34841637 | 420.76 | 51.47% | 98.69% | 95.43% |
| GC16 | 100264 | 99184 | 97838 | 67069 | 28214599 | 420.68 | 50.16% | 98.72% | 95.46% |
| GC17 | 111917 | 110772 | 109458 | 51174 | 21040352 | 411.15 | 52.45% | 98.81% | 95.80% |
| GC18 | 105802 | 104757 | 103527 | 75558 | 31578391 | 417.94 | 52.12% | 98.72% | 95.46% |
| GC19 | 102142 | 101183 | 99941 | 98730 | 41394915 | 419.27 | 53.71% | 98.82% | 95.76% |
| GC20 | 103057 | 101100 | 98466 | 92017 | 39205622 | 426.07 | 51.92% | 98.47% | 94.64% |
| GC21 | 104442 | 102052 | 99339 | 94195 | 39669576 | 421.14 | 51.76% | 98.35% | 94.43% |
| GC22 | 114587 | 112763 | 109713 | 106543 | 44261523 | 415.43 | 52.99% | 98.57% | 94.98% |
| GC23 | 104862 | 103120 | 100225 | 97604 | 41384250 | 424 | 51.77% | 98.34% | 94.32% |
| GC24 | 105997 | 103964 | 101339 | 98194 | 41118431 | 418.75 | 52.38% | 98.40% | 94.64% |
| GC25 | 103164 | 101150 | 98628 | 89585 | 37206103 | 415.32 | 50.68% | 98.35% | 94.44% |
| GC26 | 105133 | 103146 | 100589 | 89405 | 37091381 | 414.87 | 51.05% | 98.49% | 94.76% |
| GC27 | 118173 | 116329 | 113453 | 102223 | 43109062 | 421.72 | 50.72% | 98.51% | 94.79% |
| GC28 | 112535 | 111028 | 107895 | 103663 | 44081094 | 425.23 | 52.75% | 98.51% | 94.69% |
| GC29 | 110020 | 108222 | 105326 | 84047 | 34685519 | 412.69 | 51.60% | 98.54% | 94.91% |
| GC30 | 105604 | 104025 | 101475 | 80314 | 33179809 | 413.13 | 50.88% | 98.58% | 95.04% |
| GC31 | 116259 | 114186 | 111349 | 96950 | 40523098 | 417.98 | 51.39% | 98.51% | 94.86% |
| GC32 | 108231 | 106249 | 103039 | 98708 | 42072823 | 426.24 | 52.24% | 98.53% | 94.86% |
| GC33 | 105199 | 103648 | 101170 | 93017 | 39033362 | 419.64 | 51.55% | 98.54% | 95.03% |
| GC34 | 94946 | 93811 | 91761 | 80988 | 33501249 | 413.66 | 50.70% | 98.81% | 95.76% |
| GC35 | 104185 | 102769 | 100557 | 98817 | 41105313 | 415.97 | 50.39% | 98.79% | 95.63% |
| GC36 | 108989 | 107397 | 104604 | 100742 | 42836447 | 425.21 | 52.21% | 98.41% | 94.62% |
| GC37 | 101677 | 100203 | 97592 | 91872 | 38483413 | 418.88 | 51.52% | 98.55% | 95.05% |
| GC38 | 105530 | 103814 | 101248 | 93166 | 39064548 | 419.3 | 49.99% | 98.58% | 95.06% |
| GC39 | 103471 | 101791 | 99142 | 95052 | 39937774 | 420.17 | 50.86% | 98.60% | 95.19% |
| GC40 | 104847 | 102923 | 100599 | 84525 | 35351102 | 418.23 | 51.22% | 98.57% | 95.05% |
| GC41 | 106434 | 104538 | 101971 | 90476 | 37815370 | 417.96 | 51.18% | 98.52% | 94.86% |
| GC42 | 114827 | 113067 | 110385 | 89929 | 37012639 | 411.58 | 51.65% | 98.59% | 95.13% |
| GC43 | 106351 | 104593 | 102177 | 82773 | 34575544 | 417.72 | 50.70% | 98.51% | 94.91% |
| GC44 | 102539 | 100637 | 98239 | 93305 | 39139195 | 419.48 | 51.13% | 98.48% | 94.77% |
| GC45 | 103693 | 101962 | 99422 | 93678 | 39200376 | 418.46 | 50.51% | 98.52% | 94.88% |
| GC46 | 107757 | 106023 | 103354 | 100029 | 41362150 | 413.5 | 50.85% | 98.52% | 94.86% |
| GC47 | 115299 | 113150 | 109403 | 103388 | 43542480 | 421.16 | 51.56% | 97.99% | 93.60% |
| GC48 | 117179 | 114920 | 110635 | 97647 | 40484910 | 414.6 | 51.48% | 97.88% | 93.31% |
| GC49 | 123663 | 120901 | 116431 | 112553 | 47669043 | 423.53 | 50.05% | 97.86% | 93.24% |
| GC50 | 87213 | 85664 | 82775 | 70415 | 29573822 | 419.99 | 50.73% | 97.96% | 93.63% |
| GC51 | 186347 | 183065 | 176171 | 162748 | 67966140 | 417.62 | 51.07% | 97.97% | 93.52% |
| GC52 | 119436 | 116883 | 112299 | 105627 | 44789395 | 424.03 | 51.37% | 97.95% | 93.43% |
| GC53 | 132691 | 130045 | 125735 | 102126 | 43170651 | 422.72 | 50.38% | 97.94% | 93.49% |
| GC54 | 93384 | 91918 | 88683 | 76534 | 31630561 | 413.29 | 51.35% | 98.12% | 93.97% |
| GC55 | 286509 | 281674 | 272140 | 150003 | 62404281 | 416.02 | 51.43% | 98.06% | 93.88% |
| GC56 | 74938 | 73628 | 71284 | 67547 | 28357699 | 419.82 | 50.46% | 98.10% | 93.93% |
| GC57 | 78511 | 77148 | 74518 | 71895 | 29830134 | 414.91 | 51.07% | 98.03% | 93.69% |
| GC58 | 169767 | 166857 | 161070 | 147452 | 61478928 | 416.94 | 51.31% | 98.11% | 93.97% |
| GC59 | 107510 | 105864 | 102467 | 100999 | 41529491 | 411.19 | 49.23% | 98.12% | 93.94% |

**Supplemental material 2.** The statistical results for each step of the data processing are shown in the table. **rawPE** represents the original PE reads obtained from sequencing. **Combined** refers to the Tags sequences obtained after merging. **Qualified** refers to the sequences after filtering low-quality and short-length Raw Tags. **Nochime** refers to the Tags sequences after chimeric filtering, which are the final Effective Tags used for subsequent analysis. **Base** refers to the total base count of the final Effective Tags. **AvgLen** is the average length of the Effective Tags; **Q20** and **Q30** represent the percentage of bases in the Effective Tags with a quality score greater than 20 (sequencing error rate <1%) and 30 (sequencing error rate <0.1%), respectively. **GC (%)** represents the GC content in the Effective Tags.
