# Supplementary material for: Will gut, oral, and vaginal microbiota influence the outcome of FET or be influenced by FET? A pilot study
Source: mBio. 2025 Jun 17;16(7):e00509-25. doi: 10.1128/mbio.00509-25 (PMC12239586; doi:10.1128/mbio.00509-25)
Supplement: Supplemental material — Files S3 to S10. [file mbio.00509-25-s0003.pdf]

Supplimental material 3-10

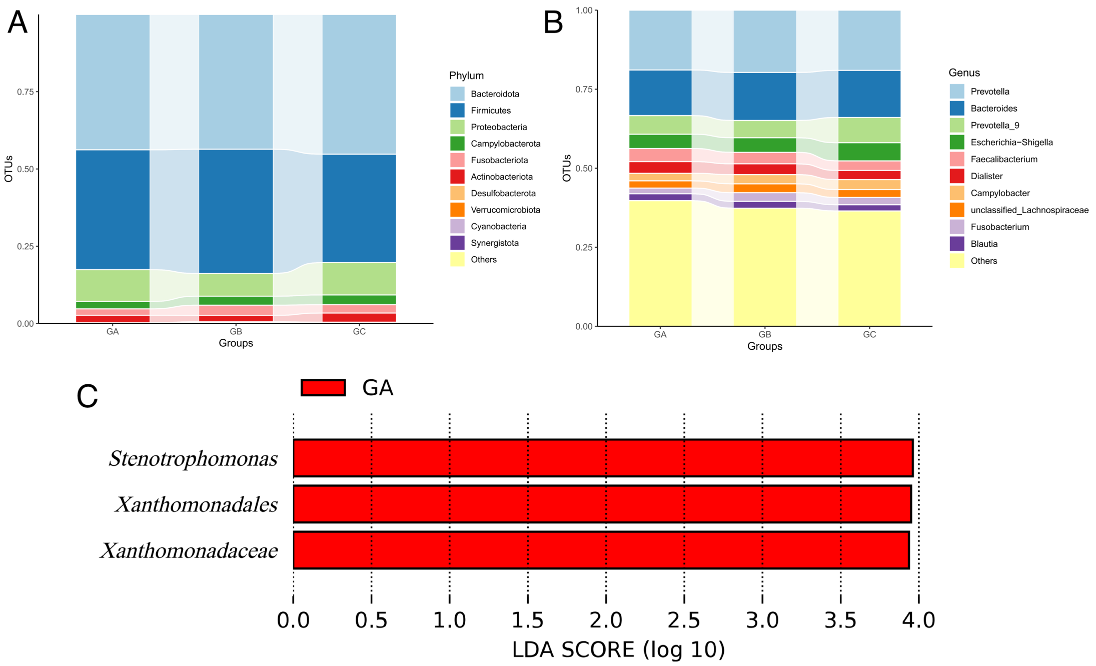

Supplemental material 3. A. Bar chart of gut microbiota in each group during FET process at the phylum level. B. Bar chart of gut microbiota in each group during FET process at the genus level. C. Bar plot showing the different taxa with an LDA score > 3 and $p < 0.05$. The distance between each point represents the degree of difference in the microbiome of each sample. The length of the bars represents the magnitude of the impact of differential species.

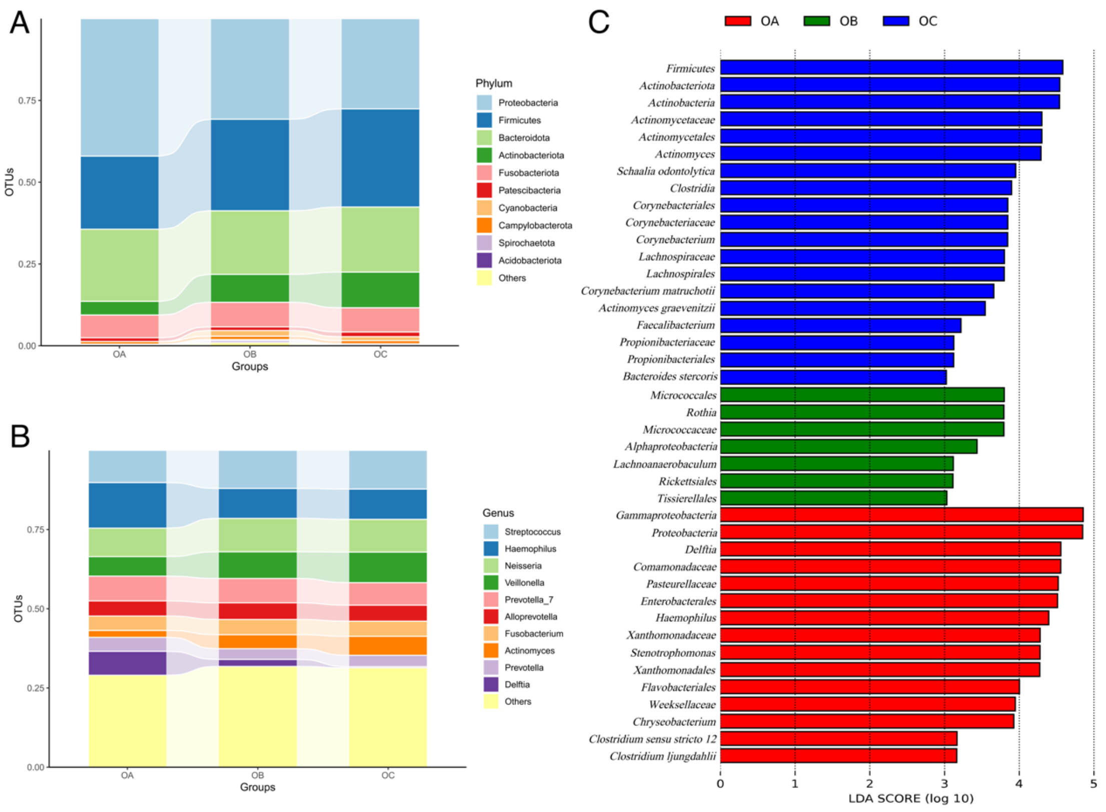

Supplemental material 4. A. Bar chart of oral microbiota in each group during FET process at the phylum level. B. Bar chart of oral microbiota in each group during FET process at the genus level. C. Bar plot showing the different taxa with an LDA score > 3 and $p < 0.05$. The distance between each point represents the degree of difference in the microbiome of each sample. The length of the bars represents the magnitude of the impact of differential species.

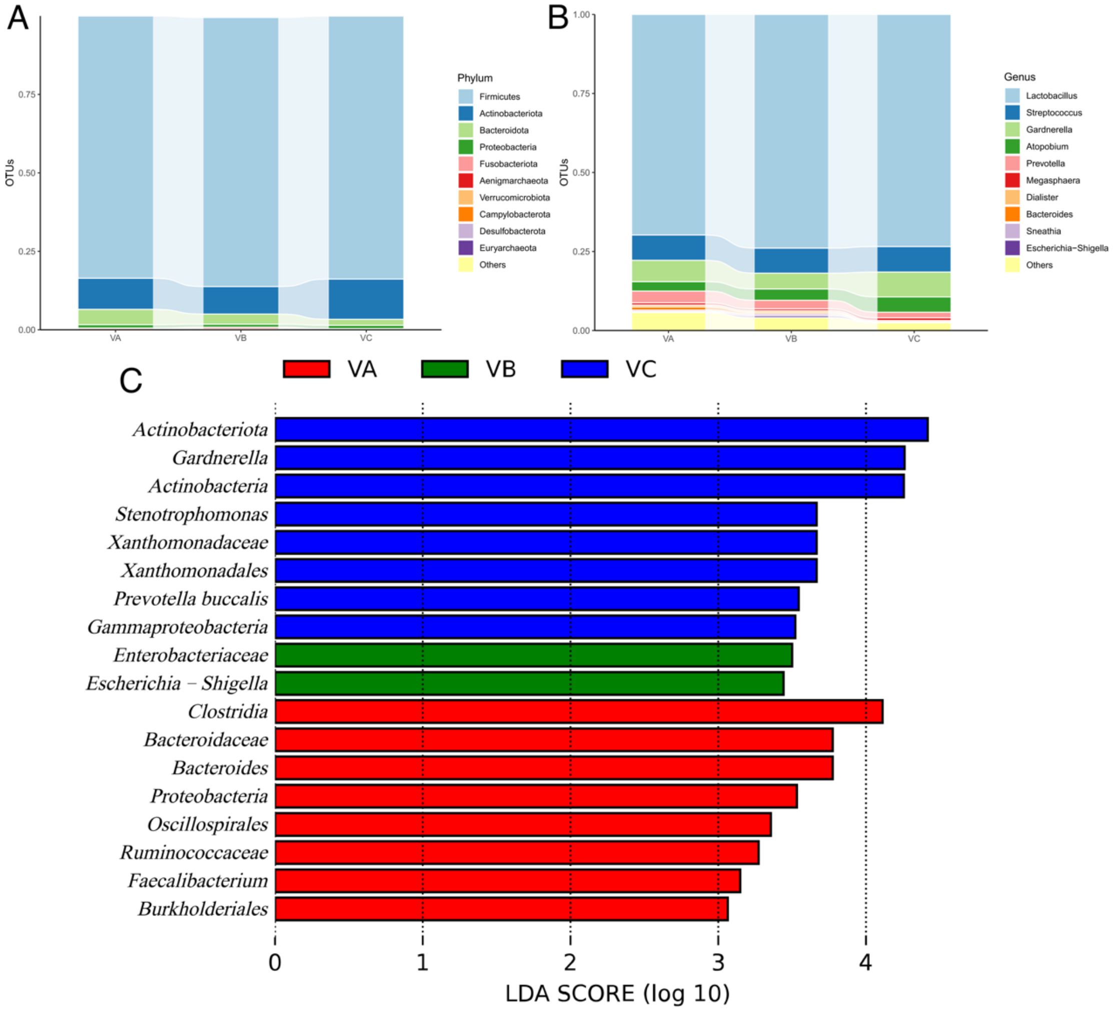

Supplemental material 5. A. Bar chart of vaginal microbiota in each group during FET process at the phylum level. B. Bar chart of vaginal microbiota in each group during FET process at the genus level. C. Bar plot showing the different taxa with an LDA score > 3 and $p < 0.05$. The distance between each point represents the degree of difference in the microbiome of each sample. The length of the bars represents the magnitude of the impact of differential species.

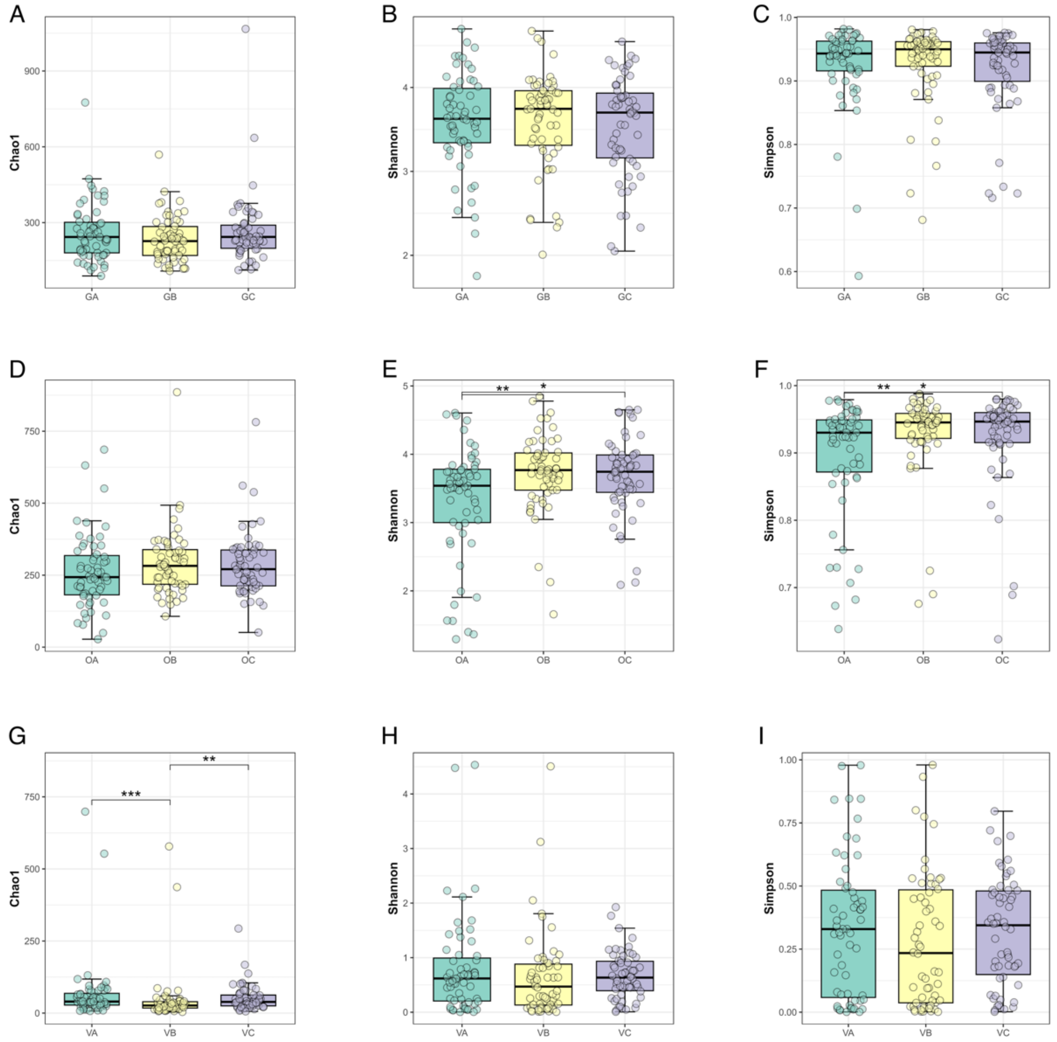

Supplemental material 6. Alpha-diversity of each microbiota during FET-process were showed as boxplot (Mean±SD). A. Chao1 index of gut microbiota in each group. B. Shannon index of gut microbiota in each group. C. Simpson index of gut microbiota in each group. D. Chao1 index of oral microbiota in each group. E. Shannon index of oral microbiota in each group. F. Simpson index of oral microbiota in each group. G. Chao1 index of vaginal microbiota in each group. H. Shannon index of vaginal microbiota in each group. I. Simpson index of vaginal microbiota in each group. *:<0.05; **:<0.01; **:<0.001.

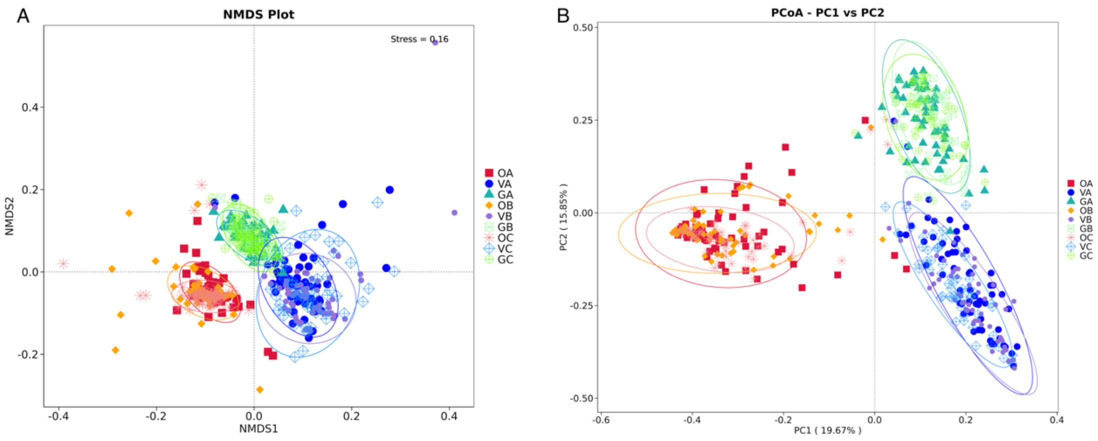

Supplemental material 7. Beta diversity (as assessed by the unWeighted UniFrac NMDS and PCoA) of sample in each group. The distance between each point represents the degree of difference in the microbiome of each sample. A. Nonmetric Multidimensional Scaling (NMDS). Stress value (0-1) is a measure of the error between the original distance and the low-dimensional spatial distance obtained by NMDS. The lower Stress value (usually <0.05) indicates a very good fit. B. Principal co-ordinates analysis method (PCoA).

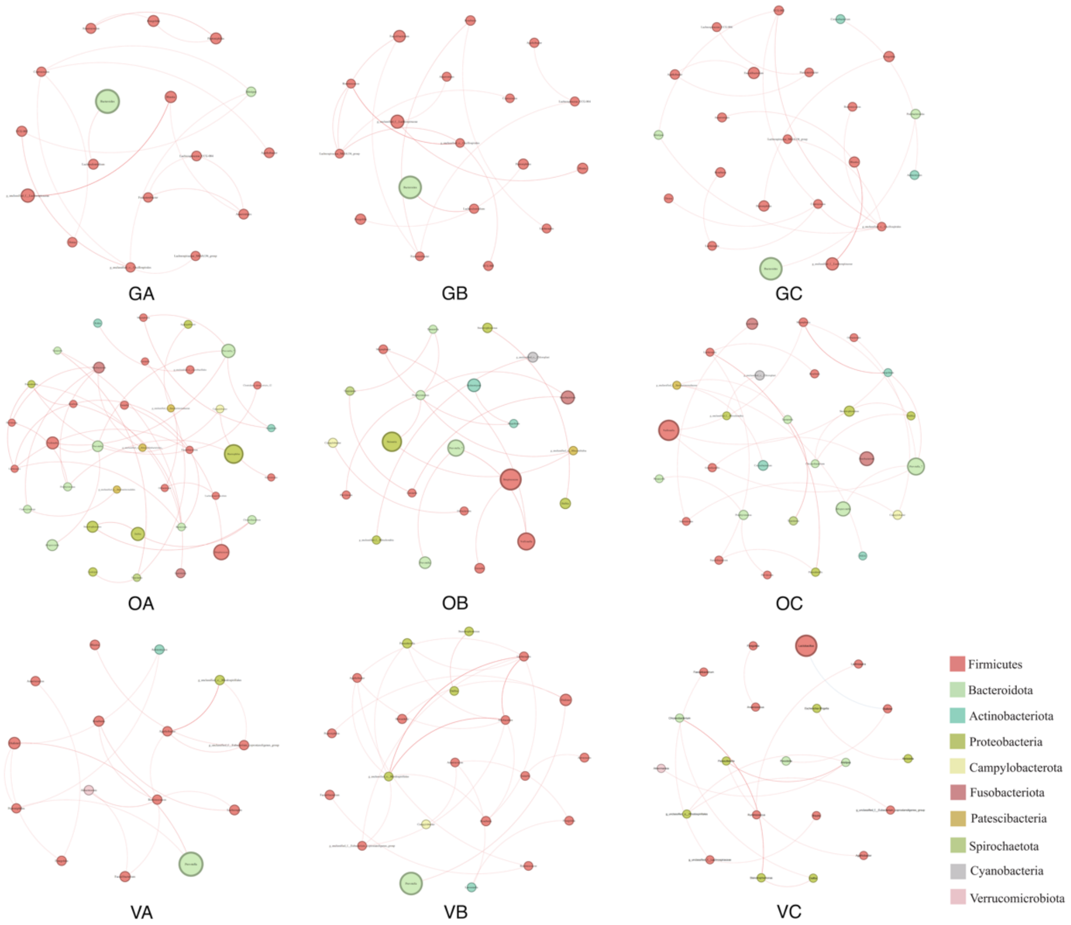

Supplemental material 8. Co-occurrence networks of the core genera (top 50) in each

group. The distance between points represents the degree of difference in the microbiome of each sample. Co-occurrence network analysis was based on the core genera (average relative abundance ＞0.005%). Each node represents a species, the node color represents the phylum, and the node size represents the relative number of OTUs. The connection line represents the presence of a significant correlation between two nodes, a Spearman correlation coefficient value below 0 (negative correlation) indicates a blue line, and a Spearman correlation coefficient value greater than 0 (positive correlation) indicates a red line. The thicker the line is, the greater the Spearman correlation coefficient is between the two nodes.

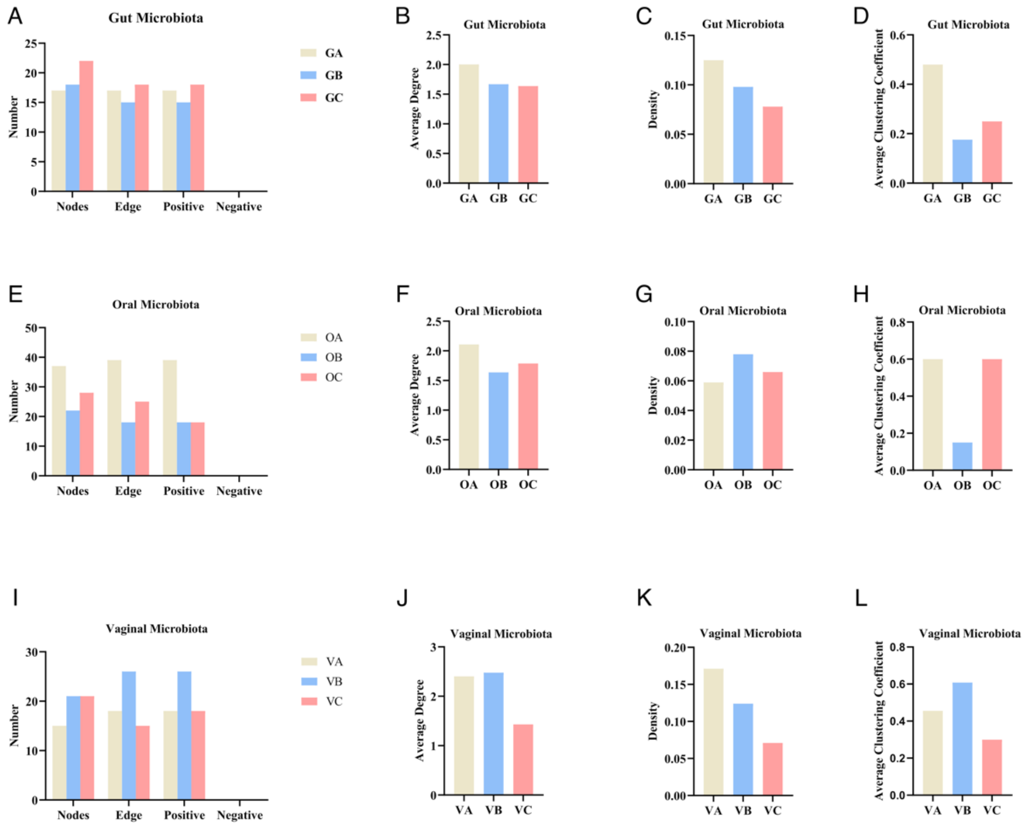

Supplemental material 9. Topological properties of microbial networks. A. Nodes, Edges, Positive and Negative correlation of gut microbiota networks. B. Average degree of gut microbiota networks. C. Density of gut microbiota networks. D. Average clustering coefficient of gut microbiota networks. E. Nodes, Edges, Positive and Negative correlation of oral microbiota networks. F. Average degree of oral microbiota networks. G. Density of oral microbiota networks. H. Average clustering coefficient of oral microbiota networks. I. Nodes, Edges, Positive and Negative correlation of vaginal

microbiota networks. J. Average degree of vaginal microbiota networks. K. Density of vaginal microbiota networks. L. Average clustering coefficient of vaginal microbiota networks.

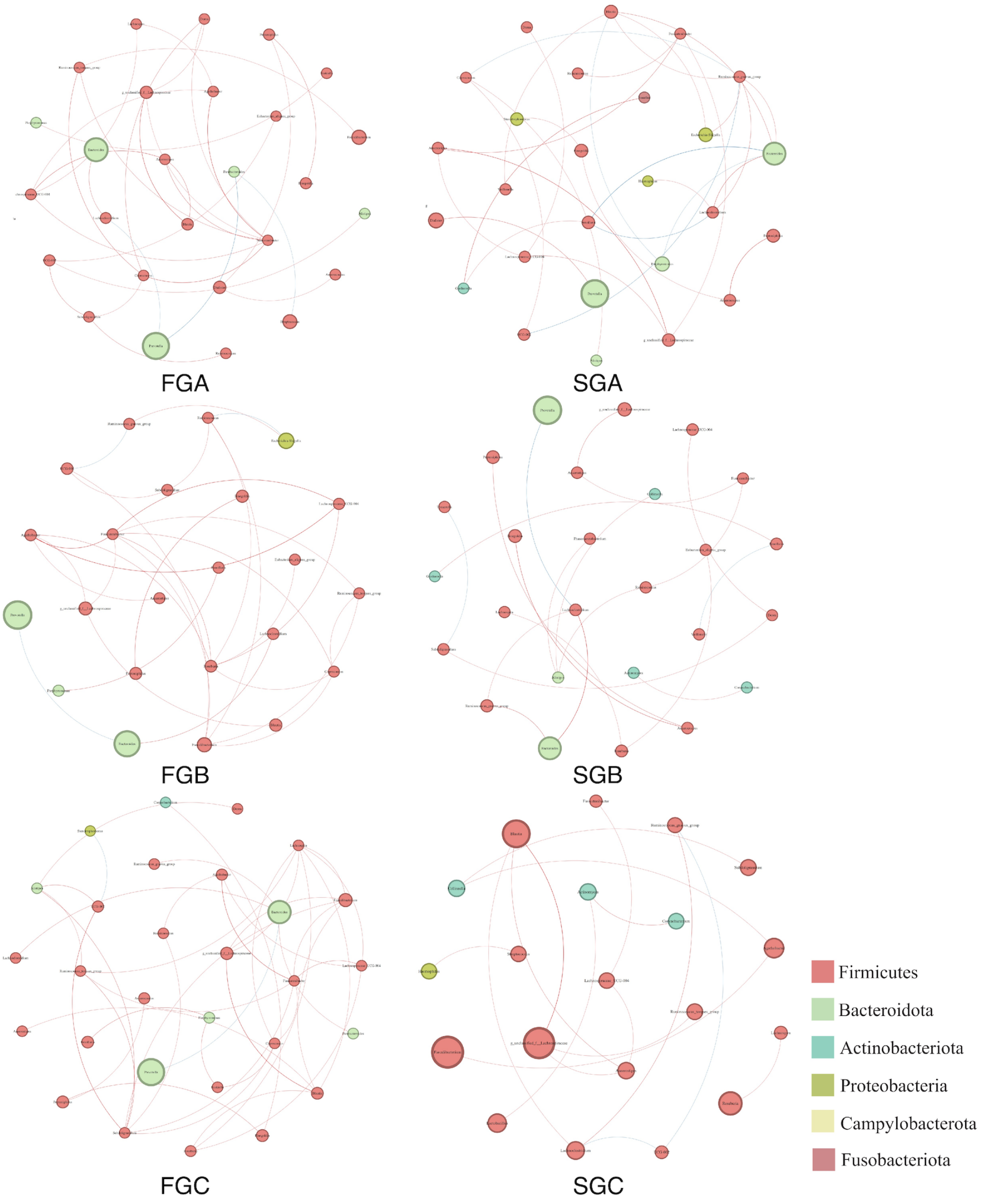

Supplemental material 10A. Co-occurrence networks of the core gut genera (top 50) in each group. The distance between points represents the degree of difference in the microbiome of each sample. Co-occurrence network analysis was based on the core genera (average relative abundance > 0.005%). Each node represents a species, the node color represents the phylum, and the node size represents the relative number of OTUs. The connection line represents the presence of a significant correlation between two nodes, a Spearman correlation coefficient value below 0

(negative correlation) indicates a blue line, and a Spearman correlation coefficient value greater than 0 (positive correlation) indicates a red line. The thicker the line is, the greater the Spearman correlation coefficient is between the two nodes.

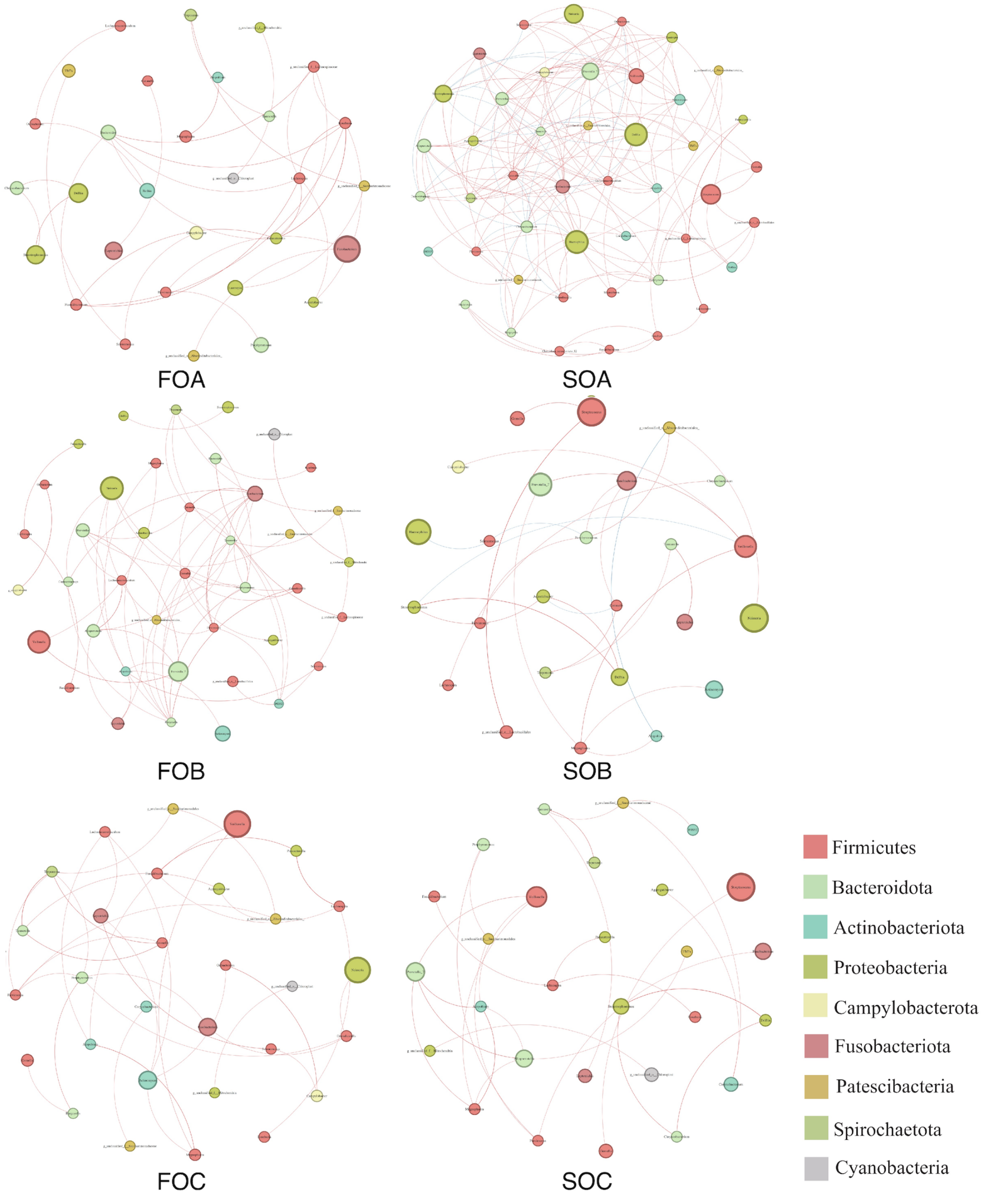

Supplemental material 10B. Co-occurrence networks of the core oral genera (top 50) in each group. The distance between points represents the degree of difference in the microbiome of each sample. Co-occurrence network analysis was based on the core genera (average relative abundance > 0.005%). Each node represents a species, the node color represents the phylum, and the node size represents the relative number of OTUs. The connection line represents the presence of a significant correlation between

two nodes, a Spearman correlation coefficient value below 0 (negative correlation) indicates a blue line, and a Spearman correlation coefficient value greater than 0 (positive correlation) indicates a red line. The thicker the line is, the greater the Spearman correlation coefficient is between the two nodes.

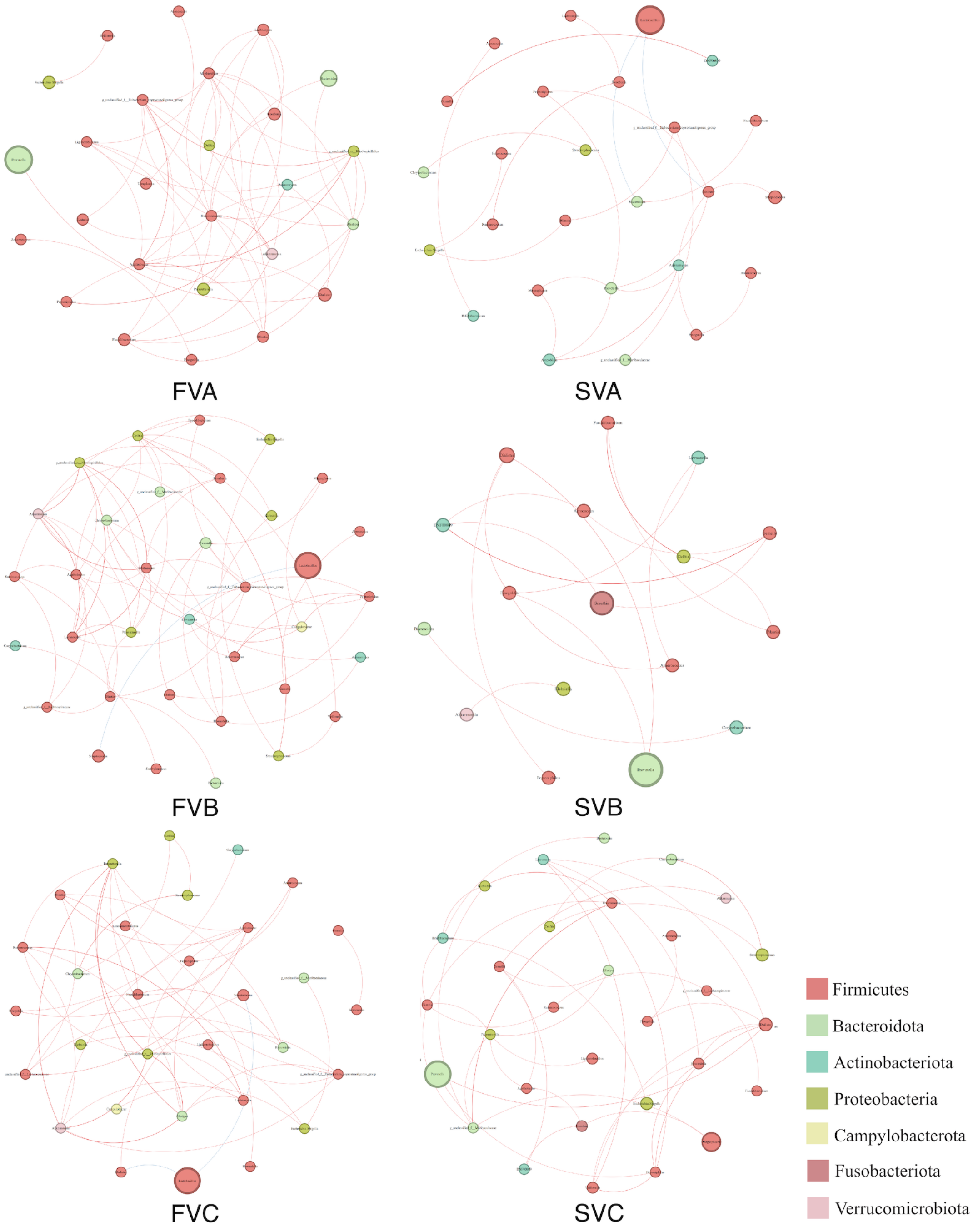

Supplemental material 10C. Co-occurrence networks of the core vaginal genera (top 50) in each group. The distance between points represents the degree of difference in the microbiome of each sample. Co-occurrence network analysis was based on the core genera (average relative abundance > 0.005%). Each node represents a species, the node color represents the phylum, and the node size represents the relative number of OTUs. The connection line represents the presence of a significant correlation between two nodes, a Spearman correlation coefficient value below 0 (negative correlation) indicates a blue line, and a Spearman correlation coefficient value greater than 0 (positive correlation) indicates a red line. The thicker the line is, the greater the Spearman correlation coefficient is between the two nodes.
